# Curcumin Inhibits ERK/c-Jun Expressions and Phosphorylation against Endometrial Carcinoma

**DOI:** 10.1155/2019/8912961

**Published:** 2019-10-31

**Authors:** Zhenxue Zhang, Pengfei Yi, Changchun Tu, Jiejie Zhan, Liping Jiang, Fanglin Zhang

**Affiliations:** ^1^School of Pharmacy, Nanchang University, Nanchang 330006, China; ^2^Pharmacy Department, Jiangxi Provincial Children's Hospital, Nanchang 330000, China

## Abstract

Curcumin has been shown to have anticancer effects in a variety of tumors. However, there are fewer studies on the role of curcumin in endometrial carcinoma (EC). The purpose of this experiment was to examine the inhibitory effect of curcumin on endometrial carcinoma cells and ERK/c-Jun signaling pathway. We first predicted the mechanism of action of curcumin on endometrial carcinoma by network pharmacology. Then, we found that curcumin can decrease the cell viability of Ishikawa cells, inhibit the migration of cancer cells, induce apoptosis, and cause cell cycle arrest in the S phase. For molecular mechanism, curcumin reduced the mRNA expression levels of ERK2 and JUN genes and inhibited the phosphorylation of ERK and c-Jun. This suggests that curcumin inhibits the proliferation of endometrial carcinoma cells by downregulating ERK/c-Jun signaling pathway activity.

## 1. Introduction

Endometrial carcinoma is a type of uterine cancer. It belongs to epithelial malignant tumors that occur in the endometrium. The most common uterine cancer is adenocarcinoma derived from the endometrial glands, accounting for 75%–80% [1]. It is one of the three major malignant tumors of the female reproductive tract, accounting for 7% of female systemic malignancies and 20%–30% of female reproductive tract malignancies. About 67% of women diagnosed with endometrial carcinoma are in the early stage, about 21% locally spread to pelvic lymph nodes and surrounding organs, and about 8% have distant metastases [2].

Curcumin is a natural phenolic compound that has been shown to be effective in cancer treatment [3, 4]. In addition, it has been reported to have antioxidant [5], anti-inflammatory [6, 7], liver protection [8], analgesia and antiarthritis [9], lipid modification [10, 11], immune regulation [12, 13], and antidiabetic [14, 15] properties. The above pharmacological properties are attributed to the reactive functional groups in the curcumin chemical structure. A large amount of research work has revealed the structural activity relationship of curcumin [16]. The main antitumor mechanisms of curcumin include inducing apoptosis and reducing tumor proliferation and invasion by inhibiting multiple cellular signaling pathways [17]. Several studies have reported the antitumor activity of curcumin on breast cancer, lung cancer, head and neck squamous cell carcinoma, prostate cancer, and brain tumors [18].

Network pharmacology, first proposed by Hopkins in 2007 [19], is a drug-designing approach that encompasses systems biology, network analysis, connectivity, redundancy, and pleiotropy [20]. It is capable of describing complex interactions among biological systems, drugs, and diseases from the perspective of an interconnected network and therefore is a fitting approach to analyze the mechanisms underlying the action of TCM formulations [21–23]. *In silico* prediction of drug targets has become popular in recent years [24, 25]. Target prediction in network pharmacology can accelerate the progress of drug design and development and address limitations [26]. Therefore, we used this approach to predict the targets of curcumin against endometrial carcinoma, in order to elucidate the possible mechanism of drug action comprehensively.

Activation of the ERK pathway is associated with the development of numerous tumors [27]. MAPK/ERK pathway activation and subsequent interactions are highly regulated but may be out of regulation in cancer cells. c-Jun is one of the downstream regulatory targets of ERK, found as the first oncogenic transcription factor [28]. The study reports that c-Jun is an important regulator of a wide range of biological processes such as cell proliferation, differentiation, invasion, migration, and apoptosis [29, 30]. And its expression and activation in cancer are highly induced, providing feedback on environmental stimuli, such as DNA damage [31].

Currently, there are few sufficient studies on curcumin inhibiting endometrial carcinoma. Curcumin has been confirmed to suppress the expression of matrix metalloproteinase to inhibit migration [32] and downregulate apoptosis-related proteins, Wnt pathway, and ROS production to induce apoptosis in endometrial carcinoma. In this study, we manage to explore the mechanism of curcumin on ERK/c-Jun pathway in EC.

## 2. Materials and Methods

### 2.1. Target Genes Prediction of Curcumin on EC

CTD is a powerful tool to analyze compound-gene, compound-protein, compound-disease, and gene-disease relationships. These data can be combined with gene function and signaling pathways to predict the mechanism of action of the drugs in various diseases [33]. The abovementioned interactions for curcumin were searched in the CTD, which returned 889 interacting genes. The top 10%, i.e., the most interactive genes, were selected as candidate target genes.

The gene expression profiles of 91 samples of pathologically reviewed stage I EC (79 endometrioid and 12 serous, with a heterogeneous distribution of grade and depth of myometrial invasion) were compared to those of 12 samples of atrophic endometrium from postmenopausal women (Supplementary [Supplementary-material supplementary-material-1], Supplemental digital content 1, which includes information of endometrial carcinoma samples and nonendometrial carcinoma samples). The EC dataset (accession no. GDS4589) [34] based on GPL570: [HG-U133_Plus_2] Affymetrix Human Genome U133 Plus 2.0 Array platform was downloaded from the Gene Expression Omnibus (GEO) database [35] (http://www.ncbi.nlm.nih.gov/geo/) (Supplementary [Supplementary-material supplementary-material-1], Supplemental digital content 2, which is about information of microarray data). The DEGs were screened using adjusted *P* value <0.01 and |logFC| >1. Target genes of curcumin on EC are overlapped between candidate target genes and DEGs.

### 2.2. GO and Pathway Analysis and PIN Construction of Overlapping Genes

Gene Set Analysis Toolkit (WebGestalt), a versatile online enrichment tool, includes overrepresentation analysis (ORA), gene set enrichment analysis (GSEA), and network topology-based analysis (NTA) which can be customized by the user. Overlapping gene list was uploaded to WebGestalt for GO analysis. Pathway enrichment analysis was performed using the Kobas 3.0 platform, which includes 9 enrichment methods: the set-based methods (GSEA, GSA, PADOG, PLAGE, GAGE, and GLOBALTEST) and net-based methods (GANPA, GGEA, and CEPA). The overlapping gene list was uploaded to the database, and the enrichment scores were calculated [36, 37]. The PPIs of the overlapping target genes were obtained from the String database with confidence 0.7 and visualized by Cytoscape version 3.6.1. The Molecular Complex Detection (MCODE) was used to screen modules of PPI network (PIN) with degree cutoff = 2, node score cutoff = 0.2, k-core = 2, and max depth = 100 [38].

In the topological analysis of PIN, a vertex or node is the fundamental unit of which graphs are formed. The degree (or valency) of a vertex of a graph is the number of edges incident to the vertex, with loops counted twice. Nodes with high values of degree over the threshold values are called “hubs.” In scale-free PINs, some proteins connect to a great number of partners compared to others, which is also named “hubs” [39, 40]. The lack of hubs may be the collapse of the entire PINs. But nonhubs are usually unnecessary for PINs [41]. Taken together, hubs are more likely to be key nodes and play a leading role in the regulation of networks [42].

### 2.3. Cell Line and Culture

Ishikawa (IK) cell line was obtained from the Chinese Academy of Science (Shanghai, China). Cells were cultured in DMEM (Gibco, MA, USA) and incubated in a professional incubator of 5% CO_2_ at 37°C with proper humidity.

### 2.4. Cell Viability Assay

The cell concentration was diluted to 3 × 10^4^ cells/ml and seeded in 96-well plates. On the next day, the concentration of dissolved curcumin (Solarbio, Beijing, China) was diluted to 80 *μ*M, 40 *μ*M, 20 *μ*M, and 10 *μ*M, respectively. The medium of the 96-well plate was replaced to the fresh medium containing the above drug concentrations. Another negative control (NC) group was set up. The 96-well plates were placed in a CO_2_ incubator for 24 h, 48 h, and 72 h. The cells were then added with 5 mg/ml MTT solution (Solarbio, Beijing, China) for 4 h and then dissolved in DMSO to detect the A490 value.

### 2.5. Transwell Assay

When cells were cultured to logarithmic growth phase and starved for 12–24 hours in serum-free medium, they were collected and counted. The concentration was adjusted to 5 × 10^5^ cells/ml. Then, 600 *μ*l of the medium containing 10% serum was added to the lower chamber, and 100 *μ*l of the diluted cell suspension was added to the upper chamber. The mixture was further cultured for 24 hours in a CO_2_ incubator. After taking out the chamber, cells were washed, fixed, dyed, and sealed and pictures were taken.

### 2.6. Flow Cytometry Was Used to Detect Cell Cycle and Apoptosis

Curcumin was diluted to 40 *μ*Μ and 20 *μ*Μ and added to medium of Ishikawa cells. NC group was changed into a drug-free medium. They were all cultured for 48 hours. After the cells were fixed and PI stained, flow cytometry was used to detect the cell cycle. Following treatment with curcumin in the same way, the cells were resuspended in PBS, stained with Annexin V and PI, and detected.

### 2.7. RT-qPCR

After treating the cells with curcumin, we extracted the RNA by Transzol regents (TransGen Biotech, Beijing, China). Absorbance analysis was used to detect RNA concentration and purity. Gel electrophoresis was used to detect RNA integrity. Then, RNA was reverse-transcribed into cDNA (Takara, Japan). PCR was performed using iTaq™ Universal SYBR Green Supermix (BIO RAD, CA, USA) with ABI 7500 Fast (Thermo Fisher, MA, USA). The relative expression of RNA of each target gene was analyzed by the 2^−△△Ct^ method. The primers are listed in Table 1.

### 2.8. Western Blot

Protease and phosphatase inhibitors were added to RIPA lysate within 2–3 min before use. Following treatment with various concentrations of curcumin, Ishikawa cells were added to the prepared lysate. The cell suspension was transferred, incubated on ice, and centrifuged to obtain a protein solution. The protein concentration was detected to calculate loading volume. We used a 5% concentrated gel and 10% separating gel to run the electrophoresis, and then, proteins were transferred to PVDF membranes. After blocking, every band of target protein was incubated in respective primary antibody solution overnight at 4°C. The membranes were washed three times with PBS and put together with appropriate secondary antibody for 1 h at room temperature. The bands were washed again and detected. Image *J* software (National Institutes of Health, MD, USA) was used for densitometric analysis.

## 3. Results

### 3.1. Identification of Potential Targets

Potential targets were predicted using CTD, and the top 10% of the 889 initial hits (89) were selected as the candidate target genes (Supplementary [Supplementary-material supplementary-material-1], Supplemental digital content 1, which presents candidate target genes of curcumin).

A total of DEGs were identified by comparing the EC and Non-EC microarray datasets, using *P* value <0.01, logFC >1, or logFC <−1 as the threshold (Supplementary [Supplementary-material supplementary-material-1], Supplemental digital content 4, which gives details for differentially expressed genes between endometrial carcinoma and nonendometrial carcinoma samples). Thirty-one DEGs overlapped with the drug target genes and were identified as the putative target genes of curcumin in EC. The overlapping gene list is shown in Table 2.

### 3.2. GO and Pathway Enrichment, PIN Construction, and MCODE Analysis

**Figure 1:**
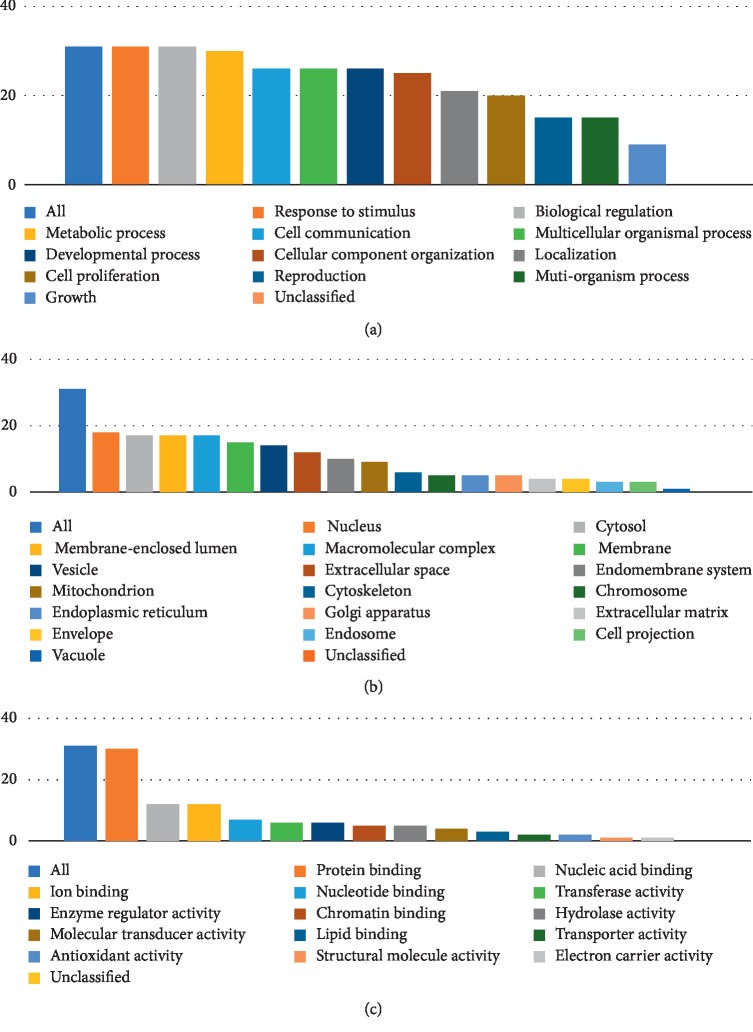
Go analysis of target genes. The *y*-axis shows the number of targeted genes enriched, and the *x*-axis shows the GO terms. Bar chart of (a) biological process categories, (b) cellular component categories, and (c) molecular function categories.

GO and pathway enrichment analyses were performed using the Gene Set Analysis Toolkit and KOBAS 3.0, respectively. As shown in Figure 1, putative overlapping genes mostly participated in metabolic processes, response to stimuli, biological regulation, cell communication, and other biological process (BP) categories. The main cellular component (CC) categories of these targets were the nucleus, cytosol, macromolecular complex, and membrane-enclosed lumen, and the primary molecular function (MF) was protein binding.

The 31 targets were enriched in 154 entries of KEGG pathways, and the top 10% are shown in Table 3. The corrected *P* value of the EC signaling pathways was 5.40*e* − 11.

**Figure 2:**
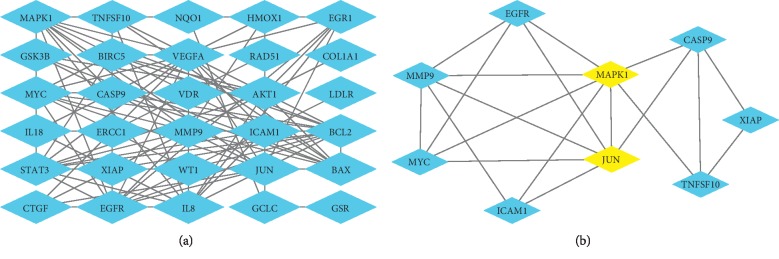
(a) Predicted targets by PIN. (b) Clusters predicted by MCODE. MAPK1 and JUN are regarded as key regulatory genes.

The overlapping gene list was analyzed by String database (version 9.1, available online: https://string-db.org/) [43] with confidence >0.7 which is a higher confidence value than the default. The results were imported to Cytoscape to construct PIN (Figure 2(a)). According to MCODE analysis (Figure 2(b)), MAPK1 and JUN were the highest interconnected hubs in the PIN, indicating more important functions compared to other genes.

### 3.3. Curcumin Inhibits the Cell Viability of Ishikawa Cells

**Figure 3:**
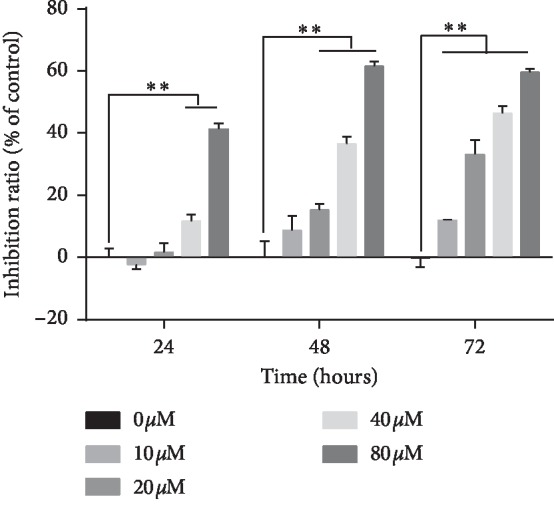
Curcumin inhibits the cellular viability of Ishikawa. Ishikawa cells were treated with 0 to 80 *μ*Μ curcumin for 24, 48, and 72 h and detected by 3-(4,5-dimethyl-2-thiazolyl)-2,5-diphenyl-2-H-tetrazolium bromide assay. The data indicated are the relative inhibition rates of the drug-treated group versus the NC group, expressed as mean ± standard deviation, from three independent replicates. ^*∗∗*^*p* < 0.01.

MTT assay was used to detect the inhibitory effect of curcumin on Ishikawa cells. The cells were treated with 0 *μ*Μ, 10 *μ*Μ, 20 *μ*Μ, 40 *μ*Μ, and 80 *μ*Μ of curcumin for 24 h, 48 h, and 72 h, respectively. High concentration groups showed inhibition after 24 h (^*∗∗*^*p* < 0.01). Following 72 h of treatment, significant inhibition was observed in all treatment groups (^*∗∗*^*p* < 0.01), as shown in Figure 3. This result indicates that curcumin can effectively reduce the proliferation of Ishikawa cells.

### 3.4. Curcumin Suppresses Motility of Ishikawa Cells

**Figure 4:**
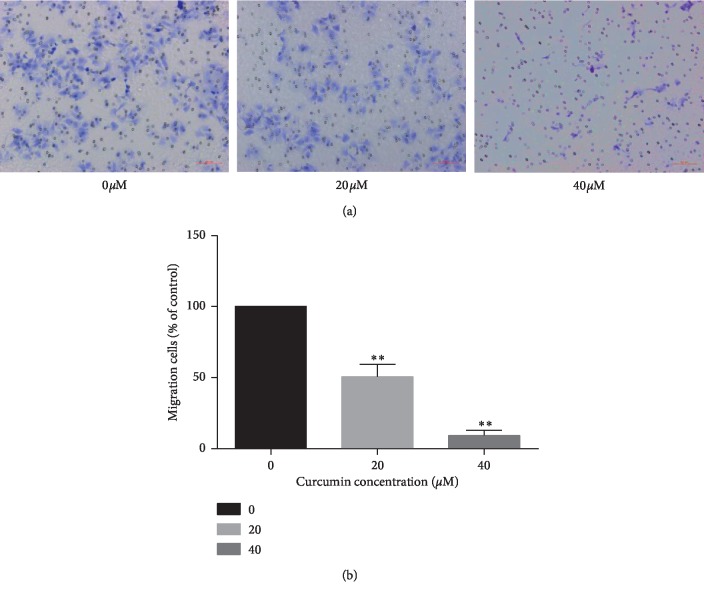
(a) Effects of curcumin on the motility of Ishikawa cells in vitro. Ishikawa cells were pretreated with 0, 20, and 40 *μ*M curcumin for 48 h. Transwell assays were used to measure the migration of cancer cells with 0.1% crystal violet staining (magnification: ×200). (b) The mobility is compared to the NC group. The values of the histograms were calculated from the data of three independent replicates and expressed as mean ± standard deviation. ^*∗∗*^*p* < 0.01.

Transwell assay detects the effects of migration on Ishikawa cells by curcumin. After treatment with 0 *μ*Μ, 20 *μ*Μ, and 40 *μ*Μ of curcumin, the migration rate of cancer cells was gradually inhibited with increasing concentration (Figure 4(a)). As shown in Figure 4(b), after 48 hours of curcumin addition, Ishikawa cells showed motility inhibition (^*∗∗*^*p* < 0.01).

### 3.5. Curcumin Induces Apoptosis of Ishikawa Cells

**Figure 5:**
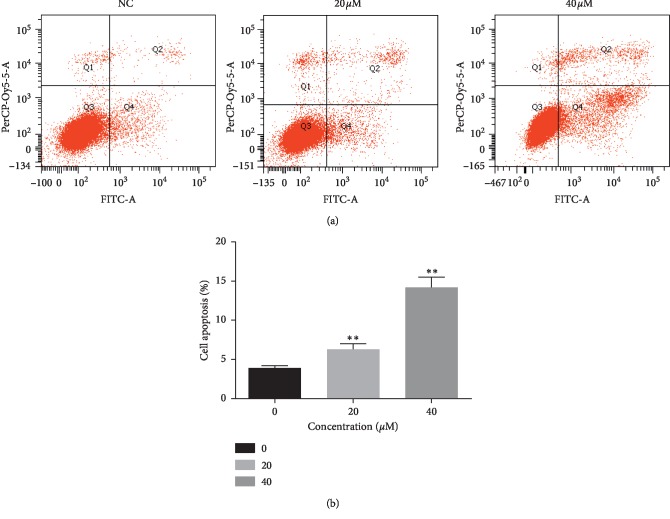
(a) Curcumin induces apoptosis in Ishikawa cells. The apoptosis of Ishikawa cells increased significantly after treatment with different concentrations of curcumin for 48 hours. (b) Compared with NC group, the values were calculated from three independent experiments and presented as mean ± SD. ^*∗*^*p* < 0.05, ^*∗∗*^*p* < 0.01.

Flow cytometry was used to detect the effect of apoptosis. Annexin V combined with PI can distinguish cells at different stages of apoptosis, namely, living cells (Annexin V−/PI−), early apoptotic cells (Annexin V+/PI−), late apoptotic cells, and necrotic cells (Annexin V+/PI+), as shown in Figure 5(a). After treatment with 0 *μ*Μ, 20 *μ*Μ, and 40 *μ*Μ of curcumin for 48 h, the number of apoptotic cells increased (Figure 5(b)), suggesting curcumin could increase the number of apoptosis in endometrial carcinoma cells (^*∗*^*p* < 0.05, ^*∗∗*^*p* < 0.01).

### 3.6. Curcumin Causes Cell Cycle Arrest in Endometrial Carcinoma Cells

**Figure 6:**
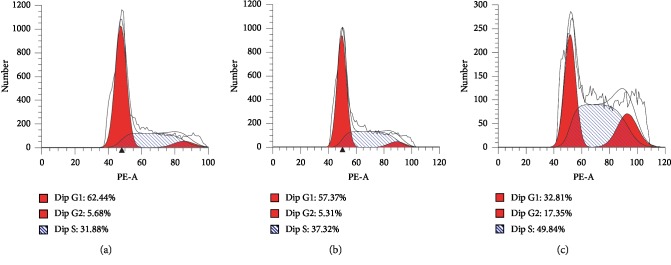
Curcumin can cause S-phase cell arrest in Ishikawa cells. After treatment with curcumin for 48 h, Ishikawa cells were subjected to PI staining and flow cytometry to detect the cell cycle. About half of Ishikawa cells appeared in the S phase. At the same time, the number of cells in the G2/M phase also increased.

Cell cycle was detected by flow cytometry by PI staining. After treatment with curcumin for 48 hours, the number of cells in the G2/M phase and S phase increased. At 40 *μ*Μ, nearly half of Ishikawa cells showed S-phase cell cycle arrest (Figure 6).

### 3.7. Curcumin Downregulates mRNA Expression Levels of ERK2 and JUN in Ishikawa Cells

**Figure 7:**
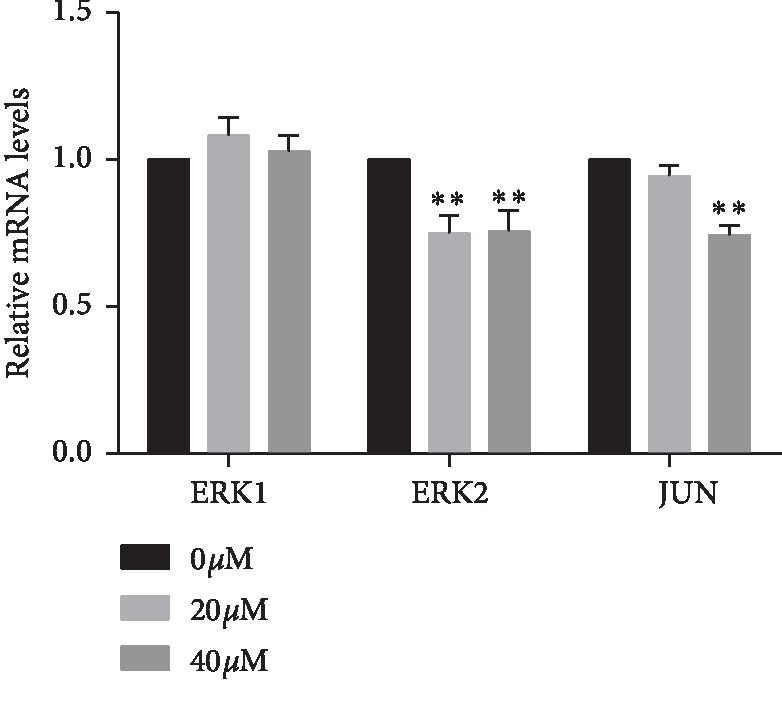
Effect of curcumin on the mRNA expression of ERK1/2 and JUN in endometrial carcinoma was detected by RT-qPCR. Curcumin reduces mRNA expression of ERK2 and JUN. The values indicated were calculated from data of three independent replicates and expressed as mean ± SD. ^*∗∗*^*p* < 0.01 was compared with the control group.

The effect of curcumin on the mRNA expression of ERK1, ERK2, and JUN was detected by RT-qPCR. After 48 hours of treatment with curcumin, the ERK2 showed inhibition (^*∗∗*^*p* < 0.01). In the 40 *μ*Μ group, the mRNA expression of JUN was apparently suppressed (^*∗∗*^*p* < 0.01). No significant inhibition was observed about ERK1 after curcumin treatment (Figure 7).

### 3.8. Curcumin Inhibits Phosphorylation of ERK1/2 and c-Jun in Ishikawa Cells

**Figure 8:**
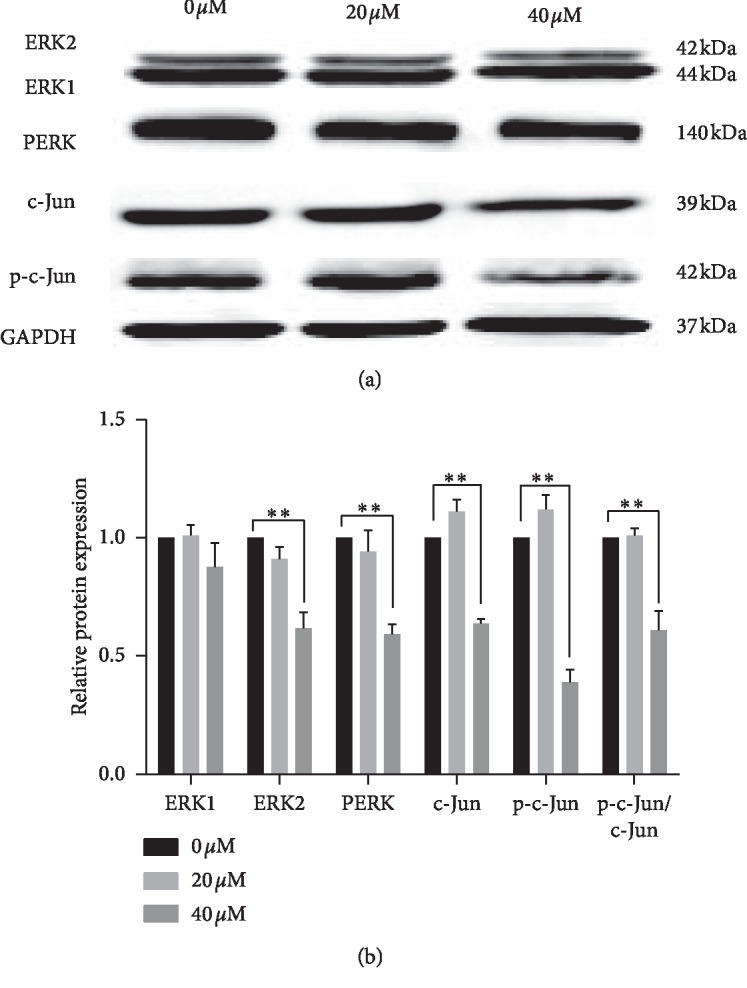
(a) Western blot was used to detect protein expression levels of ERK1/2, c-Jun, PERK, and p-c-Jun. At 40 *μ*M, the protein expression of PERK and p-c-Jun was reduced after curcumin treatment. (b) Gray value of each band was obtained from grayscale analysis. Relative protein expressions were calculated with 0 *μ*Μ group. Each experiment was repeated three times independently, and the statistical results were expressed as mean ± standard deviation, compared with expression of GAPDH. ^*∗*^*p* < 0.05 and ^*∗∗*^*p* < 0.01.

Western blot was used to detect the total protein expression of ERK1/2, c-Jun, and their phosphorylated forms (Figure 8(a)). After 48 hours of curcumin treatment, the phosphorylation levels of ERK and c-Jun were significantly downregulated in the 40 *μ*Μ group. The total protein level of c-Jun was decreased, but ERK1/2 was not statistically changed (Figure 8(b)) (^*∗*^*p* < 0.05, ^*∗∗*^*p* < 0.01).

## 4. Discussion

The first step in *in silico* drug development is the identification of target genes. Several drugs and active compounds have multiple targets [26, 44], which have been identified by *in silico* target fishing methods [45]. Eighty-nine predicted target genes (Supplementary [Supplementary-material supplementary-material-1], Supplemental digital content 3, which presents candidate target genes) of curcumin were identified of which 31, including CASP9, AKT1, GSK3B, EGFR, MYC, and MAPK1, were also differentially expressed in EC and were thus potential targets of curcumin. The overlapping genes have been listed in Table 2. Enrichment analysis of 31 target genes was found to be closely related to endometrial carcinoma signaling pathway. It also supports the result of 31 target genes in EC from another approach. Then, we constructed the PPI network of these genes to identify clusters of interacting genes. As shown in Figure 2(b), MAPK1 and JUN were both hubs. MAPK1 is also known as ERK2 and is phosphorylated by MEK.

Nearly half of the endometrial carcinoma cells were arrested in the S phase, suggesting that curcumin may cause damage to the DNA of endometrial carcinoma cells. In melanoma, curcumin induces DNA damage, apoptosis, and cytotoxicity in cancer and normal cells in a dose-dependent manner through its pro-oxidative activity. These activities are higher in cancer cells than in normal cells [46]. In lymphoma cells, curcumin induces DNA breaks. In order to resist antitumor DNA damage reagents, cancer cells usually rely on Rad51-dependent homologous recombination to repair DNA. The result showed that curcumin induced DNA damage and triggered caspase-3-dependent apoptosis by regulating Rad51-dependent homologous recombination, making lymphoma cells sensitive to various DNA damage reagents [47]. It also suppressed the cervical cancer by inducing DNA damage and chromatin condensation in vitro [48]. Stable expression of phosphorylation-deficient inactivated c-Jun in glioma cells significantly inhibits AP-1-driven transcriptional activation and greatly increases cytotoxic effect of DNA damage agents [49]. Transcriptional activation of c-Jun could be induced to give rise to apoptosis when DNA is damaged [31]. The DNA damage of endometrial carcinoma cells by curcumin needs further experimental verification.

c-Jun initiates mRNA transcription of the Bcl-X through a conserved AP-1 binding site located in its proximal promoter. There are two AP-1 binding sites in the Bcl-X gene, which are proved to be recognized by the heterodimer c-Jun·ATF2. The knockdown experiments have revealed that c-Jun and ATF2, but not c-Fos, are essential for Bcl-XL expression and induce apoptosis [50]. The ERK pathway is known to be involved in apoptosis of tumor cells. Inhibition of phosphorylation of ERK promotes apoptosis in lung cancer and pancreatic cancer cell lines [51]. In prostate cancer, inhibition of ERK-related signaling pathways also induces apoptosis [52]. Studies have found that Bcl-2 initiates antiapoptotic responses via ERK1/2-mediated pathways [53, 54]. Curcumin has been shown to promote cell apoptosis by downregulating Ets-1 and Bcl-2 in endometrial carcinoma [55]. This suggests that curcumin may regulate the expression of apoptosis-related proteins by downregulating the ERK/c-Jun signaling pathway.

Curcumin has also been reported to reduce the invasion of endometrial carcinoma cells by inhibiting the phosphorylation of ERK1/2 and downregulating the expression of MMP-2/-9. After treatment with the ERK signaling pathway inhibitor U0126, invasion of endometrial carcinoma cells and expression of MMP-2/-9 were also inhibited [56]. In glioma cells, curcumin has a broad-spectrum MMP family inhibitory effect, mainly by inhibiting the binding of AP-1 to MMP gene promoters and transcriptional activity. It suggested that curcumin inhibits MMP transcription partly mediated by AP-1 and MAPK pathways [32]. Immunohistochemical analysis of endometrial carcinoma tissues revealed that expression of c-Jun may be relevant to migration potential of EC. Its expression in EC may be helpful as a prognostic indicator [57]. Combined with the findings of this study, curcumin may downregulate the phosphorylation level of ERK/c-Jun and reduce the synthesis of AP-1, thereby reducing the transcription level of MMP-2/-9 and the invasion of endometrial carcinoma cells.

c-Jun usually plays an important role in estrogen-induced proliferation and differentiation. Stimulation of estrogen causes an increase in the expression of c-Jun [58, 59]. In human uterine tissue, the mRNA expression level of c-Jun in the endometrium changes with the menstrual cycle. The expression of c-Jun increases significantly when estrogen is ingested [60, 61]. The sustained strong expression of c-Jun prevents endometrial stromal cells from entering apoptosis [62]. Compared with the secretory phase, the protein expression of c-Jun is higher in the proliferative phase, which may be related to estrogen stimulation [62, 63]. However, some experiments have shown that the expression level of c-Jun does not change with the menstrual cycle [64]. AP-1, which is formed by c-Jun and c-Fos, is involved in the estrogen signal transduction pathway. Promoter transfection with the AP-1 site, instead of estrogen, initiates estrogen receptor (ER) transcription [62]. But the expression of c-Jun in EC is not affected by ER, suggesting a lack of association between c-Jun and ER in tumors [65]. The association of ER and c-Jun expression with hormone-regulated signaling pathways appears to be different from normal endometrium in malignant endometrial carcinoma. The mRNA expression of c-Jun in endometrial carcinoma is higher than that in normal tissues [60]. Tumor tissues with positive expression of c-Jun have a worse prognosis [57].

In the study of endometrial carcinoma tissues, ERK1, ERK2, estrogen receptor, and progesterone receptor (PR) expressions were significantly higher than in the normal control group. And ERK1 and ERK2 were positively correlated with the expression of ER and PR (*p* < 0.05). This reveals that EC patients show higher ER and PR expressions, which is associated with higher levels of ERK1 and ERK2, suggesting that the ERK pathway may be involved in the pathogenesis of EC [66].

The regulation of curcumin on ERK/c-Jun in different tumors is not completely consistent. In colon cancer, curcumin increases p-c-Jun but hardly affects PERK expression [67]. However, in breast cancer, curcumin reduces the expression of PERK and p-c-Jun [68]. In monocytic leukemia, curcumin increases the expression of PERK and p-c-Jun [69]. In this study, RT-qPCR confirmed that the expression of ERK2 and c-Jun was inhibited at the mRNA level. Phosphorylation of ERK1/2 and c-Jun was found to be suppressed by western blot. This study is the first to prove that curcumin inhibits the activity of endometrial carcinoma cells by acting on the ERK/c-Jun pathway.

Curcumin can inhibit the proliferation and migration of endometrial carcinoma cells, induce apoptosis, and result in cell cycle arrest in the S phase. In addition, curcumin reduces mRNA expression of ERK2 and JUN genes and phosphorylation of ERK/c-Jun pathway.

## 5. Conclusions

Curcumin can suppress the growth and migration of endometrial carcinoma cells and induce apoptosis and S-phase cell cycle arrest. The mechanism of action is to downregulate the phosphorylation level of ERK/c-Jun signaling pathway in EC.

## Figures and Tables

**Table 1:** Sequences of primers used for RT-qPCR analysis.

Gene name	Primer name	Primer sequences
*β*-Actin	Actin-F	F: TGGACTTCGAGCAAGAGATG
	Actin-R	R: GAAGGAAGGCTGGAAGAGTG
ERK1	ERK1-F	CATCGGCATCCGAGACATTC
	ERK1-R	TCCATCAGGTCCTGCACAAT
ERK2	ERK2-F	ACCAACCTCTCGTACATCGG
	ERK2-R	TAGGTCTGGTGCTCAAAGGG
JUN	JUN-F	CAGGTGGCACAGCTTAAACA
	JUN-R	AACTGCTGCGTTAGCATGAG

**Table 2:** Predictive target gene list of curcumin on EC.

Gene symbol	Gene name	Entrez gene
COL1A1	Collagen type I alpha 1 chain	1277
CTGF	Connective tissue growth factor	1490
NQO1	NAD(P)H quinone dehydrogenase 1	1728
EGFR	Epidermal growth factor receptor	1956
EGR1	Early growth response 1	1958
ERCC1	ERCC excision repair 1, endonuclease noncatalytic subunit	2067
AKT1	AKT serine/threonine kinase 1	207
GCLC	Glutamate-cysteine ligase catalytic subunit	2729
GSK3B	Glycogen synthase kinase 3 beta	2932
GSR	Glutathione-disulfide reductase	2936
HMOX1	Heme oxygenase 1	3162
XIAP	X-linked inhibitor of apoptosis	331
BIRC5	Baculoviral IAP repeat containing 5	332
ICAM1	Intercellular adhesion molecule 1	3383
CXCL8	C-X-C motif chemokine ligand 8	3576
IL18	Interleukin 18	3606
JUN	Jun proto-oncogene, AP-1 transcription factor subunit	3725
LDLR	Low-density lipoprotein receptor	3949
MMP9	Matrix metallopeptidase 9	4318
MYC	v-Myc avian myelocytomatosis viral oncogene homolog	4609
PECAM1	Platelet and endothelial cell adhesion molecule 1	5175
MAPK1	Mitogen-activated protein kinase 1	5594
BAX	BCL2-associated X, apoptosis regulator	581
RAD51	RAD51 recombinase	5888
BCL2	BCL2, apoptosis regulator	596
STAT3	Signal transducer and activator of transcription 3	6774
VDR	Vitamin D (1,25-dihydroxyvitamin D3) receptor	7421
VEGFA	Vascular endothelial growth factor A	7422
WT1	Wilms tumor 1	7490
CASP9	Caspase 9	842
TNFSF10	Tumor necrosis factor superfamily member 10	8743

**Table 3:** Enriched pathways of curcumin in the treatment on EC.

Term	ID	Input number	Background number	Corrected *P* value
Pathways in cancer	hsa05200	16	397	8.78*E* − 22
AGE-RAGE signaling pathway in diabetic complications	hsa04933	11	101	5.71*E* − 19
Hepatitis B	hsa05161	11	146	1.69*E* − 17
Colorectal cancer	hsa05210	9	62	1.44*E* − 16
Platinum drug resistance	hsa01524	8	75	6.38*E* − 14
Apoptosis	hsa04210	9	140	6.97*E* − 14
EGFR tyrosine kinase inhibitor resistance	hsa01521	8	81	1.07*E* − 13
Influenza A	hsa05164	9	176	4.20*E* − 13
Focal adhesion	hsa04510	9	203	1.22*E* − 12
Pancreatic cancer	hsa05212	7	66	2.78*E* − 12
Bladder cancer	hsa05219	6	41	2.52*E* − 11
Endocrine resistance	hsa01522	7	97	3.02*E* − 11
HIF-1 signaling pathway	hsa04066	7	103	4.35*E* − 11
PI3K-akt signaling pathway	hsa04151	9	342	8.19*E* − 11
Endometrial cancer	hsa05213	6	52	8.61*E* − 11

## Data Availability

The data used to support the findings of this study are available from the corresponding author upon request.

## Supplementary Materials

Supplementary Materials Supplementary Table 1 (SDC1): information of endometrial cancer samples and nonendometrial cancer samples. Supplementary Table 2 (SDC2): information of microarray data. Supplementary Table 3 (SDC3): target gene list of curcumin. Supplementary Table 4 (SDC4): differentially expressed genes between endometrial cancer and nonendometrial cancer samples.
