# Supplementary material for: Curcumin Inhibits ERK/c-Jun Expressions and Phosphorylation against Endometrial Carcinoma
Source: Biomed Res Int. 2019 Oct 31;2019:8912961. doi: 10.1155/2019/8912961 (PMC7012278; doi:10.1155/2019/8912961)
Supplement: Supplementary Materials — Supplementary Table 1 (SDC1): information of endometrial cancer samples and nonendometrial cancer samples. Supplementary Table 2 (SDC2): information of microarray data. Supplementary Table 3 (SDC3): target gene list of curcumin. Supplementary Table 4 (SDC4): differentially expressed genes between endometrial cancer and nonendometrial cancer samples. [file 8912961.f1.pdf]

Supplemental Digital Content

SDC1. Supplementary Table 1 Information of endometrial cancer samples and non-endometrial cancer samples

SDC2. Supplementary Table 2 Information of microarray data

SDC3. Supplementary Table 3 Target gene list of curcumin

SDC4. Supplementary Table 4 Differentially expressed genes between endometrial cancer and non-endometrial cancer samples

Supplementary Table 1 Information of endometrial cancer samples and non-endometrial cancer samples

Accession	Title	Source name	Race	Characteristics	Stage	Grade	Histology
GSM425837	2908-lcm	EE_IC_3_cauc_lcm	caucasian	age (years): 58	IC	3	endometrioid
GSM425838	3210-lcm	EE_IC_3_cauc_lcm	caucasian	age (years): 63	IC	3	endometrioid
GSM425839	3507-scrape	EE_IC_3_cauc_lcm	caucasian	age (years): 53	IC	3	endometrioid
GSM425840	3492-ls	EE_IC_3_cauc_lcm	caucasian	age (years): 63	IC	3	endometrioid
GSM425841	3415-scrape	EE_IC_3_cauc_lcm	caucasian	age (years): 88	IC	3	endometrioid
GSM425842	3285-lcm	EE_IC_3_cauc_lcm	caucasian	age (years): 49	IC	3	endometrioid
GSM425843	3264-lcm	EE_IB_3_cauc_lcm	caucasian	age (years): 85	IB	3	endometrioid
GSM425844	3433-lcm	EE_IB_3_cauc_lcm	caucasian	age (years): 83	IB	3	endometrioid
GSM425845	3458-scrape	EE_IB_3_cauc_lcm	caucasian	age (years): 43	IB	3	endometrioid
GSM425846	5008-scrape	EE_IB_3_cauc_lcm	caucasian	age (years): 60	IB	3	endometrioid
GSM425847	5407-lcm	EE_IB_3_aa_lcm	african american	age (years): 73	IB	3	endometrioid
GSM425848	2944-lcm	EE_IA_3_cauc_lcm	caucasian	age (years): 60	IA	3	endometrioid
GSM425849	2946-scrape	EE_IA_3_cauc_lcm	caucasian	age (years): 68	IA	3	endometrioid
GSM425850	3025-lcm	EE_IA_3_cauc_lcm	caucasian	age (years): 63	IA	3	endometrioid
GSM425851	G630	EE_IA_3_cauc_lcm	caucasian	age (years): unknown	IA	3	endometrioid

GSM425852	G501	EE_IA_3_cauc_lcm	caucasian	age (years): unknown	IA	3	endometrioid
GSM425853	3509-scrape	EE_IC_2_cauc_lcm	caucasian	age (years): 57	IC	2	endometrioid
GSM425854	3068-scrape	EE_IC_2_aa_lcm	african american	age (years): 65	IC	2	endometrioid
GSM425855	2933-ls	EE_IC_2_aa_lcm	african american	age (years): 72	IC	2	endometrioid
GSM425856	2795-lcm	EE_IC_2_cauc_lcm	caucasian	age (years): 80	IC	2	endometrioid
GSM425857	2861-scrape	EE_IC_2_cauc_lcm	caucasian	age (years): 47	IC	2	endometrioid
GSM425858	5021-lcm	EE_IC_2_cauc_lcm	caucasian	age (years): 61	IC	2	endometrioid
GSM425859	5025-scrape	EE_IC_2_aa_lcm	african american	age (years): 59	IC	2	endometrioid
GSM425860	5192-scrape	EE_IC_2_amind_lcm	american indian	age (years): 60	IC	2	endometrioid
GSM425861	5320-scrape	EE_IC_2_cauc_lcm	caucasian	age (years): 57	IC	2	endometrioid
GSM425862	G007	EE_IC_2_cauc_lcm	caucasian	age (years): unknown	IC	2	endometrioid
GSM425863	3729-lcm	EE_IB_2_cauc_lcm	caucasian	age (years): 66	IB	2	endometrioid
GSM425864	3783-lcm	EE_IB_2_cauc_lcm	caucasian	age (years): 57	IB	2	endometrioid
GSM425865	3587-ls	EE_IB_2_cauc_lcm	caucasian	age (years): 48	IB	2	endometrioid
GSM425866	3583-lcm	EE_IB_2_cauc_lcm	caucasian	age (years): 60	IB	2	endometrioid
GSM425867	3116-scrape	EE_IB_2_cauc_lcm	caucasian	age (years): 55	IB	2	endometrioid

GSM425868	3080-lcm	EE_IB_2_cauc_lcm	caucasian	age (years): 48	IB	2	endometrioid
GSM425869	3024-lcm	EE_IB_2_unkwn_lcm	unknown	age (years): 64	IB	2	endometrioid
GSM425870	3461	EE_IB_2_cauc_lcm	caucasian	age (years): 62	IB	2	endometrioid
GSM425871	3379-scrape	EE_IB_2_aa_lcm	african american	age (years): 69	IB	2	endometrioid
GSM425872	3406-lcm	EE_IB_2_cauc_lcm	caucasian	age (years): 75	IB	2	endometrioid
GSM425873	5258-lcm	EE_IB_2_cauc_lcm	caucasian	age (years): 59	IB	2	endometrioid
GSM425874	3125-lcm	EE_IA_2_cauc_lcm	caucasian	age (years): 56	IA	2	endometrioid
GSM425875	3339-lcm	EE_IA_2_cauc_lcm	caucasian	age (years): 64	IA	2	endometrioid
GSM425876	3769	EE_IA_2_aa_lcm	african american	age (years): 47	IA	2	endometrioid
GSM425877	3269-lcm	EE_IA_2_aa_lcm	african american	age (years): 65	IA	2	endometrioid
GSM425878	3496-lcm	EE_IA_2_aa_lcm	african american	age (years): 74	IA	2	endometrioid
GSM425879	3172-scrape	EE_IA_2_aa_lcm	african american	age (years): 37	IA	2	endometrioid
GSM425880	3217-lcm	EE_IA_2_cauc_lcm	caucasian	age (years): 65	IA	2	endometrioid
GSM425881	3409-lcm	EE_IA_2_cauc_lcm	caucasian	age (years): 49	IA	2	endometrioid
GSM425882	3986-lcm	EE_IA_2_cauc_lcm	caucasian	age (years): 56	IA	2	endometrioid
GSM425883	5410-lcm	EE_IA_2_cauc_lcm	caucasian	age (years): 77	IA	2	endometrioid

GSM425884	5178-scrape	EE_IA_2_aa_lcm	african american	age (years): 79	IA	2	endometrioid
GSM425885	G518	EE_IA_2_cauc_lcm	caucasian	age (years): unknown	IA	2	endometrioid
GSM425886	3716-lcm	EE_IC_1_cauc_lcm	caucasian	age (years): 67	IC	1	endometrioid
GSM425887	3638-lcm	EE_IC_1_cauc_lcm	caucasian	age (years): 64	IC	1	endometrioid
GSM425888	5062-scrape	EE_IC_1_cauc_lcm	caucasian	age (years): 63	IC	1	endometrioid
GSM425889	G039-lcm	EE_IC_1_cauc_lcm	caucasian	age (years): unknown	IC	1	endometrioid
GSM425890	G643-lcm	EE_IC_1_cauc_lcm	caucasian	age (years): unknown	IC	1	endometrioid
GSM425891	G016	EE_IC_1_cauc_lcm	caucasian	age (years): unknown	IC	1	endometrioid
GSM425892	G205	EE_IC_1_cauc_lcm	caucasian	age (years): unknown	IC	1	endometrioid
GSM425893	3799-lcm	EE_IB_1_cauc_lcm	caucasian	age (years): 60	IB	1	endometrioid
GSM425894	3789-scrape	EE_IB_1_his_lcm	hispanic	age (years): 47	IB	1	endometrioid
GSM425895	3749-lcm	EE_IB_1_cauc_lcm	caucasian	age (years): 52	IB	1	endometrioid
GSM425896	3557-ls	EE_IB_1_cauc_lcm	caucasian	age (years): 54	IB	1	endometrioid
GSM425897	3514-lcm	EE_IB_1_cauc_lcm	caucasian	age (years): 66	IB	1	endometrioid
GSM425898	3527-lcm	EE_IB_1_cauc_lcm	caucasian	age (years): 60	IB	1	endometrioid
GSM425899	3348-scrape	EE_IB_1_cauc_lcm	caucasian	age (years): 67	IB	1	endometrioid
GSM425900	3510-lcm	EE_IB_1_cauc_lcm	caucasian	age (years): 55	IB	1	endometrioid

GSM425901	3748-lcm	EE_IB_1_cauc_lcm	caucasian	age (years): 46	IB	1	endometrioid
GSM425902	3735-lcm	EE_IB_1_cauc_lcm	caucasian	age (years): 62	IB	1	endometrioid
GSM425903	3112-lcm	EE_IB_1_cauc_lcm	caucasian	age (years): 57	IB	1	endometrioid
GSM425904	3094-lcm	EE_IB_1_cauc_lcm	caucasian	age (years): 58	IB	1	endometrioid
GSM425905	3010-lcm	EE_IB_1_cauc_lcm	caucasian	age (years): 58	IB	1	endometrioid
GSM425906	3598	EE_IB_1_cauc_lcm	caucasian	age (years): 61	IB	1	endometrioid
GSM425907	3765-ls	EE_IA_1_cauc_lcm	caucasian	age (years): 57	IA	1	endometrioid
GSM425908	3386-scrape	EE_IA_1_aa_lcm	african american	age (years): 71	IA	1	endometrioid
GSM425909	3278-scrape	EE_IA_1_cauc_lcm	caucasian	age (years): 49	IA	1	endometrioid
GSM425910	3526-lcm	EE_IA_1_cauc_lcm	caucasian	age (years): 51	IA	1	endometrioid
GSM425911	2907-lcm	EE_IA_1_cauc_lcm	caucasian	age (years): 49	IA	1	endometrioid
GSM425912	5046-lcm	EE_IA_1_aa_lcm	african american	age (years): 50	IA	1	endometrioid
GSM425913	5072-scrape	EE_IA_1_cauc_lcm	caucasian	age (years): 58	IA	1	endometrioid
GSM425914	5113-scrape	EE_IA_1_cauc_lcm	caucasian	age (years): 58	IA	1	endometrioid
GSM425915	5278-lcm	EE_IA_1_cauc_lcm	caucasian	age (years): 45	IA	1	endometrioid
GSM425916	G026-lcm	PS_IB_3_aa_lcm	african american	age (years): unknown	IB	3	papillary serous

GSM425917	G029-lcm	PS_IA_2_aa_lcm	african american	age (years): unknown	IA	2	papillary serous
GSM425918	G206-lcm	PS_IB_3_asian_lcm	asian	age (years): unknown	IB	3	papillary serous
GSM425919	G425	PS_IA_3_cauc_lcm	caucasian	age (years): unknown	IA	3	papillary serous
GSM425920	G519-lcm	PS_IA_3_aa_lcm	african american	age (years): unknown	IA	3	papillary serous
GSM425921	G609-lcm	PS_IB_3_cauc_lcm	caucasian	age (years): unknown	IB	3	papillary serous
GSM425922	G668-lcm	PS_IA_2_cauc_lcm	caucasian	age (years): unknown	IA	2	papillary serous
GSM425923	G710-lcm	PS_IB_2_cauc_lcm	caucasian	age (years): unknown	IB	2	papillary serous
GSM425924	G929-lcm	PS_I_3_amind_lcm	american indian	age (years): unknown	I	3	papillary serous
GSM425925	3416-lcm	PS_IB_3_aa_lcm	african american	age (years): 51	IB	3	papillary serous
GSM425926	3381-scrape	PS_IB_3_aa_lcm	african american	age (years): 91	IB	3	papillary serous
GSM425927	3007-ls	PS_IB_3_aa_lcm	african american	age (years): 73	IB	3	papillary serous
GSM425928	TP02-402	NL_cauc_lcm	caucasian	age (years): 50-55	n/a	n/a	atrophic endometrium
GSM425929	TP02-478	NL_cauc_lcm	caucasian	age (years): 70-75	n/a	n/a	inactive endometrium
GSM425930	TP06-612	NL_aa_lcm	african america	age (years): 50-55	n/a	n/a	inactive endometrium
GSM425931	TP06-708	NL_cauc_lcm	caucasian	age (years): 50-55	n/a	n/a	inactive endometrium

GSM425932	TP06-727-993	NL_cauc_lcm	caucasian	age (years): 55-60	n/a	n/a	inactive endometrium
GSM425933	TP06-855	NL_cauc_lcm	caucasian	age (years): 60-65	n/a	n/a	atrophic endometrium
GSM425934	TP06-872-lcm	NL_cauc_lcm	caucasian	age (years): 75-80	n/a	n/a	weakly proliferative w/cystic changes
GSM425935	TP06-963	NL_cauc_lcm	caucasian	age (years): 85-90	n/a	n/a	cystic atrophy with sessile polyp
GSM425936	TP06-987	NL_cauc_lcm	caucasian	age (years): 50-55	n/a	n/a	secretory endometrium
GSM425937	TP06-998	NL_cauc_lcm	caucasian	age (years): 75-80	n/a	n/a	atrophic endometrium
GSM425938	TP07-152	NL_cauc_lcm	caucasian	age (years): 50-55	n/a	n/a	secretory endometrium
GSM425939	TP07-726B	NL_aa_lcm	african american	age (years): 55-60	n/a	n/a	inactive endometrium

Supplementary Table 2 Information of microarray data

Item	Description
Title	Gene Expression Analysis of Stage I Endometrial Cancers
Organism	Homo sapiens
Experiment type	Expression profiling by array
Overall design	Ninety-one samples of pathologically reviewed stage I endometrial cancers (79 endometrioid and 12 serous) with a heterogeneous distribution of grade and depth of myometrial invasion (i.e. 9 IAG1, 14 IAG2, 7 IAG3, 14 IBG1, 12 IBG2, 13 IBG3, 7 ICG1, 10 ICG2, and 6 ICG3) were examined in relation to 12 samples of atrophic endometrium from postmenopausal women. Specimens were analyzed using oligonucleotide array analysis.
Contributor(s)	Risinger JH, Chandran U, Allard J, Miller C, Sherman M, Zahn C, Oliver J, Banerjee S, Litzi T, Carlson J, Farley J, Rose S, Berchuck A, Kohn E, Day R, Conrads T, Herberman R, Maxwell GL
Citation(s)	Day RS, McDade KK, Chandran UR, Lisovich A et al. Identifier mapping performance for integrating transcriptomics and proteomics experimental results. *BMC Bioinformatics* 2011 May 27; 12:213. PMID: 21619611
Submission date	9-Jul-09
Last update date	9-Aug-18
Contact name	Uma Chandran
E-mail	chandran@pitt.edu
Phone	412-648-9326

Organization name	University of Pittsburgh
Department	BioMedical Informatics
Street address	5150 Center Ave
City	Pittsburgh
State/province	PA
ZIP/Postal code	15232
Country	USA
Platforms	GPL570 [HG-U133_Plus_2] Affymetrix Human Genome U133 Plus 2.0 Array

Supplementary Table 3 target gene list of curcumin

Gene Symbol	Gene ID	Interaction Count
TNF	7124	158
HMOX1	3162	129
NFE2L2	4780	77
PTGS2	5743	77
RELA	5970	73
BCL2	596	66
CASP3	836	66
IL1B	3553	66
IL6	3569	53
NOS2	4843	50
BAX	581	45
CCND1	595	42
NQO1	1728	42
AKT1	207	41
MMP9	4318	41
MAPK1	5594	37
VEGFA	7422	37
CAT	847	36
MAPK3	5595	36
PPARG	5468	34
TP53	7157	32
PARP1	142	31
BCL2L1	598	30
CDKN1A	1026	30

AR	367	27
NFKBIA	4792	27
GCLM	2730	25
CYP1A1	1543	24
ICAM1	3383	24
NFKB1	4790	24
DDIT3	1649	23
CXCL8	3576	22
EGFR	1956	22
ERBB2	2064	22
APP	351	20
CASP9	842	20
STAT3	6774	20
KLK3	354	19
TNFSF10	8743	19
XIAP	331	19
BIRC5	332	18
TGFB1	7040	18
GCLC	2729	17
AKR1B1	231	16
CTNNB1	1499	16
HIF1A	3091	16
SOD2	6648	16
COL1A1	1277	15
ABCB1	5243	14
AGT	183	14
GSTP1	2950	14

MMP2	4313	14
MPO	4353	14
PRKCD	5580	14
AHR	196	13
CCL2	6347	13
CDKN1B	1027	13
GSK3B	2932	13
INS	3630	13
JUN	3725	13
CD36	948	12
EGF	1950	12
MAPK8	5599	12
EP300	2033	11
IFNG	3458	11
RAD51	5888	11
ACHE	43	10
BIRC2	329	10
BIRC3	330	10
CASP8	841	10
EGR1	1958	10
HDAC1	3065	10
ERCC1	2067	9
PECAM1	5175	9
TLR4	7099	9
VDR	7421	9
ARNT	405	8
CFTR	1080	8

CTGF	1490	8
FN1	2335	8
GSR	2936	8
IL18	3606	8
LDLR	3949	8
MYC	4609	8
TNFRSF10B	8795	8
WT1	7490	8
ABCC1	4363	7
ALB	213	7
CYP3A4	1576	7

Supplementary Table 4 Differentially expressed genes between endometrial cancer and non-endometrial cancer samples

Gene.symbol	ID	adj.P.Val	logFC
SRRM4	237828_at	1.66E-17	4.5077497
TRH	206622_at	1.28E-14	5.0327823
IKZF5	226680_at	2.02E-14	1.8201264
MIR4738	211998_at	2.05E-14	3.068935
ARMCX5-GPRASP2	228027_at	6.54E-13	2.3468483
HK2	202934_at	6.54E-13	-3.2045957
GLUL	200648_s_at	1.64E-12	-2.1674298
PEG3	209242_at	1.64E-12	4.3857136
PDS5B	204742_s_at	1.69E-12	2.2102581
SDR39U1	213398_s_at	3.23E-12	1.3191013
MIR503	227488_at	3.71E-12	4.093846
HAND2-AS1	236141_at	5.59E-12	4.3891633
HSPA4	208815_x_at	6.47E-12	-1.3512293
KPNA5	229317_at	6.48E-12	1.5953228
SMIM11A	228239_at	7.28E-12	1.6259954
MAP4K4	206571_s_at	7.28E-12	-1.7996607
SNHG4	228012_at	8.7E-12	1.2436975
BCL2L11	1558143_a_at	9.13E-12	-2.0297297
COA5	225409_at	9.13E-12	1.0830811
KPNA2	201088_at	9.38E-12	-1.5056451
SOX15	206122_at	1.11E-11	2.9977777
TACC3	218308_at	1.54E-11	-2.7342267
PCDH10	1556329_a_at	1.58E-11	5.7785827

Gene.symbol	ID	adj.P.Val	logFC
EIF4G1	208624_s_at	0.00024	-1.1092037
LOC728743	225909_at	0.00024	1.1928677
MICAL2	236475_at	0.000243	-1.8485981
PCDH18	225975_at	0.000244	1.4525726
OTUD7A	232562_at	0.000244	2.1062752
PER3	1569701_at	0.000245	2.1889559
XIAP	206536_s_at	0.000246	-1.2906932
OR51E1	229768_at	0.000251	-1.2902219
HTR2B	206638_at	0.000251	2.3313949
DDX52	212834_at	0.000252	-1.0223017
RIMS1	231986_at	0.000253	1.4495367
TIMP3	201150_s_at	0.000255	1.8663161
COX7B	239252_at	0.000256	1.2672813
XPNPEP3	220020_at	0.000256	-1.1141576
CXCL6	206336_at	0.000258	-2.6451556
ALG13	205583_s_at	0.000259	1.3975609
ITPR2	202660_at	0.00026	1.7939393
CENPI	207590_s_at	0.00026	-1.7614041
B4GALT5	221485_at	0.000261	-1.1312803
PTCH1	209816_at	0.000261	1.6438512
CD163	215049_x_at	0.000261	-1.8031279
PCDH17	205656_at	0.000261	-1.4828266
LOC101927811	230446_at	0.000263	1.6183988

OVCA2	222041_at	1.7E-11	1.7354933	TIMM23B	218118_s_at	0.000264	-1.2155236
IDH2	210046_s_at	2.25E-11	-2.3900956	LOC257396	236076_at	0.000265	1.2195377
TOP2A	201291_s_at	2.79E-11	-3.5263918	RANGRF	223554_s_at	0.000265	1.2002761
MGC70870	242136_x_at	2.79E-11	2.21796	TSR1	218156_s_at	0.000267	-1.0875757
GTSE1	204315_s_at	3.63E-11	-2.7546691	SLC13A5	228844_at	0.000267	1.8537757
GFPT1	202721_s_at	3.78E-11	-2.2372692	EGFR	224999_at	0.000269	1.3313662
TSC22D1	239123_at	4.52E-11	2.9008823	IGF1R	225330_at	0.000269	1.3112411
USP30	227572_at	4.52E-11	1.1949762	ZNF506	238493_at	0.00027	1.9521892
CFL1	200021_at	4.54E-11	-1.3961096	C9orf3	212848_s_at	0.00027	1.3867671
RNFT2	221909_at	4.55E-11	-3.3798591	LINC00865	232239_at	0.00027	2.0079661
N4BP2L2	202259_s_at	4.55E-11	1.7495881	PRRT2	227192_at	0.000271	1.1629471
APH1B	226358_at	4.55E-11	1.7645448	TYMS	1554696_s_at	0.000272	-1.5193037
LEPROT	209894_at	5.15E-11	3.1288242	LRRC70	238488_at	0.000273	1.3320938
HCFC2	235264_at	6.06E-11	1.6211135	LINC01354	239822_at	0.000273	2.5248452
PROSER1	218420_s_at	6.45E-11	-1.5425706	HEMK1	218621_at	0.000273	1.881231
DUSP1	201041_s_at	6.58E-11	3.1072446	ACD	204617_s_at	0.000274	1.042556
KLHDC1	1552733_at	6.82E-11	2.4177182	TMEM33	222642_s_at	0.000275	-1.0708914
CWF19L2	228916_at	6.82E-11	1.26512	FCGR1CP	216950_s_at	0.000275	-1.6332094
ADHFE1	227113_at	7.66E-11	2.7313251	LEMD2	224980_at	0.000276	-1.0776756
ALG10B	228941_at	7.66E-11	1.4983147	MAP7	202889_x_at	0.000276	-1.6501292
ZNF25	228185_at	8.16E-11	1.4578724	SLC25A27	231787_at	0.000277	2.2415857
GPALPP1	229891_x_at	9.22E-11	1.6750874	TMEM100	219230_at	0.000277	2.2519323
PCM1	228905_at	9.61E-11	2.0896605	ATL3	223452_s_at	0.000278	-1.0807188
FAM63B	226873_at	9.61E-11	1.5864782	HSPA6	117_at	0.000278	-1.3473615
ZNF436-AS1	229086_at	1.08E-10	1.8756075	ECHDC2	235305_s_at	0.000279	1.2537485
UBXN4	212008_at	1.22E-10	-1.3138781	DENND5B	228551_at	0.00028	1.3006771
ANLN	222608_s_at	1.3E-10	-3.2642378	BTRC	216091_s_at	0.000281	-1.1235467

SPAG9	225339_at	1.39E-10	1.2987209	TSC22D1-AS1	237745_at	0.000281	1.2854754
G3BP1	201514_s_at	1.58E-10	-1.4967452	ABCG1	204567_s_at	0.000283	-1.4042646
WBP4	203599_s_at	1.73E-10	1.2715974	AP1M2	65517_at	0.000283	-1.0434027
HN1	217755_at	1.99E-10	-2.7773985	ZC3H8	239644_at	0.000283	1.1928199
ZNF302	218490_s_at	1.99E-10	2.041215	SCD	211708_s_at	0.000284	-2.1093552
MELK	204825_at	1.99E-10	-3.4724608	PDGFRA	203131_at	0.000284	1.9776074
VSIG2	229369_at	1.99E-10	3.7528414	TRAPPC2L	238628_s_at	0.000285	1.3692161
NIPBL	213918_s_at	2.09E-10	-1.5843116	MMP26	220541_at	0.000286	2.8061749
UGGT1	218257_s_at	2.11E-10	-1.6862183	GSPT2	205541_s_at	0.00029	2.6546351
DERL1	219402_s_at	2.17E-10	-1.5519907	CDC5L	209055_s_at	0.000291	-1.1829462
VCP	208649_s_at	2.27E-10	-2.0035345	HGF	209960_at	0.000291	2.1806549
PCDH10	228635_at	2.61E-10	4.6208916	SLC30A5	243166_at	0.000292	1.1954817
GSTM5	205752_s_at	2.72E-10	1.9128569	STON1	213413_at	0.000293	1.5791858
SCRN2	228730_s_at	3.04E-10	1.5964973	ERAP1	214012_at	0.000293	1.9198651
ARGLU1	227448_at	3.4E-10	1.7082008	CACNA1D	1555993_at	0.000294	1.3208314
AMT	204294_at	3.41E-10	1.9456881	COX7A1	204570_at	0.000294	1.8191766
SRSF7	201129_at	3.56E-10	1.2642366	RUFY2	233191_at	0.000294	1.0945295
CLK4	228751_at	3.93E-10	1.6425037	ESYT2	1558511_s_at	0.000294	-1.0059999
FAM110A	226584_s_at	3.93E-10	-1.8626661	GPC6	227059_at	0.000294	2.7018533
CDC20	202870_s_at	3.94E-10	-2.4632757	CERS6	235463_s_at	0.000294	-1.0162786
METAP1D	227805_at	4.22E-10	2.0314106	COL27A1	225288_at	0.000294	1.939399
PAK3	236277_at	4.39E-10	3.7706911	FABP5	202345_s_at	0.000294	-1.9136378
ST3GAL5	203217_s_at	4.82E-10	2.561826	AKT3	212609_s_at	0.000294	1.4474471
GPALPP1	223606_x_at	4.82E-10	1.2804523	SSFA2	236207_at	0.000295	-2.1802177
TPX2	210052_s_at	4.95E-10	-2.9917523	TNFRSF21	218856_at	0.000297	-1.2406519
MIGA1	235125_x_at	4.95E-10	1.651361	RAD21	200607_s_at	0.0003	-1.0924787
NEK2	204641_at	5.07E-10	-2.9828846	INHBA	210511_s_at	0.000304	-2.7858678

CELF5	230497_at	5.1E-10	3.284772	SFRP5	207468_s_at	0.000308	1.6955584
ARID4A	230141_at	5.24E-10	2.0704842	CCDC168	1561846_s_at	0.000309	-2.096374
F11R	224097_s_at	5.41E-10	-1.7215027	KPNA4	209653_at	0.00031	-1.5109079
SPSB3	46256_at	5.52E-10	1.448147	EVC2	229974_at	0.000311	1.7278986
CDCA2	226661_at	6.67E-10	-2.6660771	RBM11	232549_at	0.000311	1.9895172
CDC25A	204695_at	7.25E-10	-2.7850119	ESRP1	219121_s_at	0.000311	-1.1077172
GOPC	225022_at	7.25E-10	1.0239025	VCAN	215646_s_at	0.000312	-2.3185812
TSPAN4	209263_x_at	7.26E-10	1.372817	PPP1R3E	227412_at	0.000312	1.1412936
E2F2	228361_at	8.13E-10	-2.2909608	LINC00665	242462_at	0.000312	1.8189739
SEL1L3	212311_at	8.64E-10	-1.9887361	CHEK1	205393_s_at	0.000313	-1.6184854
ITCH	217094_s_at	8.83E-10	-1.7670602	CCDC157	1568796_at	0.000313	-1.5360401
CRBN	222533_at	9.84E-10	1.0537644	LOC101928403	236990_at	0.000313	1.1584961
CPSF2	233208_x_at	9.84E-10	-2.1445682	NR4A2	216248_s_at	0.000314	1.7609499
NTN4	223315_at	1.01E-09	2.4852705	SDS	205695_at	0.000314	-1.5857936
CENPF	207828_s_at	1.04E-09	-2.3270785	PGAP1	239725_at	0.000315	1.688215
TBX3	225544_at	1.07E-09	2.217044	MCM10	222962_s_at	0.000315	-2.2288527
MASP1	232224_at	1.22E-09	2.6864129	PRPF38A	235612_at	0.000315	1.071095
GAS2L3	235709_at	1.22E-09	-3.2153886	RAB11FIP1	219681_s_at	0.000318	-1.5935388
SRSF6	208804_s_at	1.36E-09	1.3868938	C3orf52	219474_at	0.000318	-1.582506
METTL3	209265_s_at	1.36E-09	1.3661975	SNX2	232049_at	0.000319	2.3187241
DMRTC1B	1553998_at	1.37E-09	2.3700968	TTLL7	219882_at	0.000319	-2.3018823
MORF4L2-AS1	231088_at	1.56E-09	2.189281	LOC728705	239343_at	0.00032	1.6972371
MICAL2	212473_s_at	1.59E-09	-2.8366934	SOX4	213668_s_at	0.000322	-1.1697028
FOSB	202768_at	1.61E-09	4.8369065	GCH1	204224_s_at	0.000323	-1.1431934
SF3B1	229303_at	1.67E-09	1.1166621	OTUD1	231035_s_at	0.000324	1.7025988
MCM4	212141_at	1.67E-09	-2.943458	AQP9	205568_at	0.000325	-1.7654925
LOC105377832	1556148_s_at	1.67E-09	1.701538	SGO1	231938_at	0.000326	-1.7249157

AGBL3	232395_x_at	1.71E-09	1.9617763	ANKRD22	239196_at	0.000326	-1.5979001
SEPSECS	227982_at	1.71E-09	1.2911196	GPR84	223767_at	0.000326	-1.7275897
GADD45B	207574_s_at	1.73E-09	2.449092	CDCP1	234932_s_at	0.000329	-1.2315114
WWTR1	202134_s_at	1.93E-09	-2.5760653	NIM1K	230864_at	0.000329	1.7495337
RRM2	209773_s_at	1.94E-09	-3.166423	TTC34	1565728_at	0.000329	1.7804155
ASPM	219918_s_at	1.94E-09	-3.7609575	MUM1	221290_s_at	0.00033	1.158286
MARCKS	201668_x_at	2.05E-09	-2.6147829	ETS2	241193_at	0.000331	1.7905865
EPT1	1555274_a_at	2.12E-09	-1.9894318	ADAM10	202604_x_at	0.000331	-1.0229993
CBX7	212914_at	2.12E-09	1.8818875	BOK	223349_s_at	0.000333	1.5738956
MKI67	212022_s_at	2.32E-09	-3.3559321	CSNK2A1	212075_s_at	0.000333	-1.2783874
CYR61	201289_at	2.36E-09	2.3900296	TMC5	219580_s_at	0.000334	-2.3673636
OSR2	213568_at	2.37E-09	3.0085622	ADAT3	230634_x_at	0.000335	-1.0573135
KIAA1107	214098_at	2.46E-09	1.3936794	CUX2	213920_at	0.000335	2.934003
KIF14	236641_at	2.46E-09	-2.8418302	BUB1B	203755_at	0.000335	-1.4527118
NR4A1	202340_x_at	2.46E-09	2.577264	LHX2	206140_at	0.000335	-2.7740851
KAT2A	202182_at	2.46E-09	1.8770378	BZW1	200776_s_at	0.000336	-1.2003786
SLC52A2	218151_x_at	2.72E-09	-1.4491492	ADH1B	209613_s_at	0.000336	2.5901321
LOC101928963	235607_at	2.8E-09	1.6957658	MIR612	225239_at	0.000337	-2.1986899
DUSP2	204794_at	2.97E-09	2.9634703	DCUN1D1	222678_s_at	0.000339	-1.8599919
CHD9	212615_at	3.08E-09	1.3547823	EAF1	226952_at	0.000339	-1.0652027
KATNBL1	218791_s_at	3.19E-09	1.1717527	ACP1	215227_x_at	0.00034	-1.0366536
AFDN-AS1	210409_at	3.19E-09	2.0448116	HOOK3	224359_s_at	0.000342	-1.2937829
TRPC1	205802_at	3.3E-09	2.7402381	RNASE1	201785_at	0.000343	-1.6051202
DLGAP5	203764_at	3.35E-09	-3.1976166	DNAJC12	218976_at	0.000343	1.9120186
SRSF11	200685_at	3.47E-09	1.7090234	DCN	211813_x_at	0.000345	1.7695145
SEC62	225352_at	3.49E-09	1.3721946	LOC101930112	214494_s_at	0.000345	1.0196899
LOXL4	227145_at	3.57E-09	1.947588	PLA2G6	215938_s_at	0.000346	1.2447879

SCAI	228174_at	3.6E-09	1.3789489	AHSA2	230148_at	0.000348	1.365801
SLC6A2	216611_s_at	3.7E-09	4.9045347	LINC00323	240068_at	0.000348	2.5961071
R3HDM4	55705_at	3.71E-09	-1.1919532	LOC102725108	1559952_x_at	0.000349	1.5589246
HSPA9	200692_s_at	3.79E-09	-1.6749073	TCIRG1	204158_s_at	0.000349	1.4188263
SFXN3	1559993_at	3.83E-09	1.5695441	PRPF40A	233080_s_at	0.000351	-1.0703582
ZNF571	206648_at	3.83E-09	1.3784199	MICA	205905_s_at	0.000354	1.3341326
MIR3671	239130_at	4.33E-09	3.1335945	LINC01355	244490_at	0.000355	1.6153853
NFYB	244704_at	4.45E-09	1.5443855	FZD10-AS1	243351_at	0.000356	1.144997
TBC1D15	230072_at	4.81E-09	1.3914779	MAGI2-AS3	227554_at	0.000356	1.0150055
SFN	33322_i_at	4.81E-09	-2.0279525	SLC7A11	209921_at	0.000356	-1.754543
CEP55	218542_at	5.51E-09	-3.3446563	CCDC18-AS1	227074_at	0.000356	1.3389117
RIOK2	222663_at	5.58E-09	1.5702739	NUDT7	228855_at	0.000358	1.2366005
DNAJC5	224611_s_at	5.76E-09	-1.0285839	FOXRED2	231846_at	0.000358	-1.3148019
UNC13C	1556096_s_at	5.88E-09	3.8917864	CCDC50	228693_at	0.00036	1.0678522
DPY19L4	226721_at	6.27E-09	1.4102238	CEP68	212677_s_at	0.000361	1.1912405
CPEB1	219578_s_at	6.53E-09	2.8436753	ZNF214	220497_at	0.000362	1.9395184
ADAMTS9-AS2	1556364_at	6.54E-09	3.3926989	BBS4	212745_s_at	0.000362	1.36902
TIGAR	219099_at	6.54E-09	-1.7494383	MTMR11	1556034_s_at	0.000362	2.0421238
GAS6	1598_g_at	6.54E-09	2.5193856	OMD	205908_s_at	0.000362	2.0902608
KPNA2	211762_s_at	6.72E-09	-2.2987102	PRICKLE1	226069_at	0.000362	1.7406378
MAP4	243_g_at	6.78E-09	-1.111707	ERG	213541_s_at	0.000362	1.4757464
ADAMTS9-AS2	1559296_at	7.1E-09	3.2894312	MIR3652	200598_s_at	0.000364	-1.2046931
TCEAL7	227705_at	7.16E-09	3.8030119	LPCAT1	201818_at	0.000365	-1.6577188
NADK	213607_x_at	7.16E-09	-2.1342038	RERGL	220276_at	0.000367	2.3121704
RRM2	201890_at	7.16E-09	-3.4045592	EZH2	203358_s_at	0.000367	-1.1704238
FOS	209189_at	7.36E-09	3.9406006	LOC100507311	244655_at	0.000369	2.1680905
YWHAZ	200641_s_at	7.56E-09	-1.4347215	RPS21	200834_s_at	0.000369	1.0015475

FLJ37453	227593_at	7.69E-09	1.6555696	FSTL3	203592_s_at	0.00037	1.4160453
NDC80	204162_at	7.74E-09	-2.5318281	PDLIM5	216804_s_at	0.00037	-1.3424766
HNMT	228772_at	8.26E-09	2.7476868	CBX5	209715_at	0.000371	-1.1972282
C21orf2	203996_s_at	8.28E-09	1.989574	RAB35	205461_at	0.000371	-1.9148463
ING5	228287_at	8.28E-09	1.6158536	FLJ10038	236164_at	0.000372	1.0950731
RNFT2	221908_at	8.28E-09	-2.2948028	DDX17	230180_at	0.000372	1.4757103
RRN3P1	216902_s_at	8.58E-09	2.2410045	GMDS	214106_s_at	0.000375	-1.3476305
FAM169A	235048_at	8.63E-09	2.5775647	CCL15-CCL14	205392_s_at	0.000377	1.678603
RANBP9	216125_s_at	8.93E-09	-1.8555829	ARIH2	216008_s_at	0.000378	-1.3322571
R3HDM4	221764_at	8.97E-09	-1.9212982	UBQLNL	236965_at	0.000378	2.0031546
DCN	209335_at	9.35E-09	3.2411481	TWIST2	229404_at	0.000378	1.792534
KIF20A	218755_at	9.35E-09	-2.4101672	CHEK2	210416_s_at	0.000378	-1.2217635
GPRASP1	204793_at	1.05E-08	1.9421366	HEY1	218839_at	0.000378	-2.4976646
PTTG1	203554_x_at	1.05E-08	-2.0503935	SLC31A1	203971_at	0.000379	-1.2143919
TUB	228882_at	1.05E-08	2.823809	LDHA	200650_s_at	0.000379	-1.1905113
FOXM1	202580_x_at	1.06E-08	-2.8985831	PRTG	229178_at	0.00038	2.5112988
TPST1	204140_at	1.08E-08	1.374946	SPARCL1	200795_at	0.00038	1.6692961
SLC7A6	203578_s_at	1.11E-08	-1.8105432	ANKHD1-EIF4EBP3	233292_s_at	0.000381	-1.5674903
N4BP2L1	213375_s_at	1.13E-08	2.0950262	NR3C1	201865_x_at	0.000381	1.2284483
LINC00899	232088_x_at	1.17E-08	2.9271468	ZNF175	243128_at	0.000383	1.5838551
PNP	201695_s_at	1.22E-08	-1.8822964	PIGL	232262_at	0.000385	1.4105652
SLC35F6	204962_s_at	1.22E-08	-2.7298005	CDK6	243000_at	0.000388	1.4356512
MIR424	229784_at	1.23E-08	2.2501497	LOC728392	218380_at	0.000389	1.5054817
FAM72A	225834_at	1.23E-08	-2.5634442	IVNS1ABP	206245_s_at	0.000392	-1.1779342
JUN	201466_s_at	1.25E-08	2.124324	TOP1	208900_s_at	0.000393	-1.3941444
CYBRD1	222453_at	1.33E-08	2.5518544	BCL2	203685_at	0.000395	1.8588227

ZNF662	228538_at	1.34E-08	3.1660818	COX20	224820_at	0.000395	1.0550485
ACACA	212186_at	1.43E-08	-1.2192059	LIN7B	219760_at	0.000396	1.1451752
ANKRA2	218769_s_at	1.45E-08	1.1454788	SLC4A7	207604_s_at	0.000399	-1.1297566
HN1	222396_at	1.48E-08	-1.4732814	TCEAL2	211276_at	0.0004	2.7422139
EIF3J-AS1	235124_at	1.48E-08	1.8202525	FIGN	222956_at	0.0004	-1.8509962
TUBE1	226181_at	1.5E-08	1.4446621	WDR1	200611_s_at	0.000401	-1.0353741
UBE2C	202954_at	1.58E-08	-1.996391	TMEM192	1555790_a_at	0.000401	1.0734282
KIF15	219306_at	1.65E-08	-2.75554	APOE	203382_s_at	0.000402	-1.6604534
PTTG3P	208511_at	1.85E-08	-2.2367715	CENPN	222118_at	0.000402	-1.5174804
HSPH1	208744_x_at	1.92E-08	-2.3648565	POLI	219317_at	0.000402	1.087729
CCDC50	226713_at	2.07E-08	1.3767565	HECW2	236089_at	0.000404	-1.99173
BPTF	230056_at	2.08E-08	1.5874423	SDPR	222717_at	0.000404	2.5569358
SLC38A1	224580_at	2.08E-08	-2.5629204	HIPK1	212291_at	0.000404	-1.6769125
PIK3C3	229705_at	2.12E-08	1.4339888	DNAJC3	235341_at	0.000407	-1.2089167
ATG5	202512_s_at	2.12E-08	1.3197353	THY1	213869_x_at	0.000411	-1.302952
ZCCHC7	226496_at	2.15E-08	1.1912651	SEC61A2	219499_at	0.000411	-1.0340588
SMAD4	235725_at	2.15E-08	1.4138557	SLC7A6	203579_s_at	0.000414	-1.2663652
DGKE	238694_at	2.19E-08	1.8294827	MSN	200600_at	0.000414	-1.357398
STAT5B	212549_at	2.25E-08	1.1138509	RNF157-AS1	230776_at	0.000419	1.6210531
SUGT1P3	1554143_a_at	2.28E-08	2.7279325	NME3	204862_s_at	0.00042	1.2751346
THUMPD1	213025_at	2.3E-08	1.1229325	CDK7	211297_s_at	0.00042	-1.0235082
PNISR	212179_at	2.36E-08	1.4691511	GLIDR	228040_at	0.000422	1.1520699
VDAC1	212038_s_at	2.39E-08	-2.3144359	RBPMS	231961_at	0.000422	1.7885154
HAND2-AS1	219791_s_at	2.4E-08	4.7904165	ACE	227463_at	0.000422	1.7701189
MTERF4	214364_at	2.4E-08	1.6986849	LILRB2	210146_x_at	0.000423	-1.8139726
SLC6A2	217621_at	2.42E-08	5.6074171	ERMP1	218342_s_at	0.000424	-1.1449473
C14orf28	235369_at	2.57E-08	1.2965668	YPEL4	235916_at	0.000424	1.942004

LOC100270804	229722_at	2.57E-08	1.9614121	HIST1H4H	208180_s_at	0.000425	-1.7635145
LINC00893	1556026_at	2.62E-08	2.7331643	SNRPE	231112_at	0.000426	1.8088627
BAIAP2L1	227371_at	2.62E-08	-1.7942652	LINC00957	1564207_at	0.000427	1.2409874
KLHDC2	217906_at	2.66E-08	1.2049874	NSUN6	230200_at	0.000427	1.4651108
ACTR3	200996_at	2.66E-08	-1.5550792	CXCL17	226960_at	0.000429	-2.0987245
NCAPG	218663_at	2.68E-08	-2.9903186	HAVCR2	235458_at	0.00043	-2.023861
PEX3	203970_s_at	2.74E-08	1.3651277	THEMIS2	210785_s_at	0.000431	-1.2199554
IL13RA1	211612_s_at	2.74E-08	-1.4862868	MATN1-AS1	1557558_s_at	0.000435	1.3355828
TNXB	213451_x_at	2.75E-08	2.4856819	COLCA2	228338_at	0.000436	1.3785822
ID4	209292_at	2.83E-08	3.3618979	LOC102725451	1569348_at	0.000436	1.5987358
LOC100505851	1568647_at	2.84E-08	2.5962007	WHSC1	209052_s_at	0.000437	-1.8749873
LOC100996419	1559535_s_at	3.01E-08	1.8636815	LOC101927137	239297_at	0.000437	2.2004033
HACE1	227471_at	3.01E-08	1.1914586	ECHDC1	219974_x_at	0.000438	-1.0802913
SFN	33323_r_at	3.04E-08	-2.5241223	ZNF506	221626_at	0.000438	1.175738
SURF4	222977_at	3.07E-08	-1.0784894	PKD1L2	244444_at	0.000438	2.6526981
IFI30	201422_at	3.13E-08	-2.3283512	EFR3B	227283_at	0.000442	-1.4826227
SKA3	227165_at	3.15E-08	-1.6669652	KIF12	229258_at	0.000445	1.9273482
RBBP6	232044_at	3.26E-08	1.0988385	HSP90AA1	211968_s_at	0.000445	-1.0057964
ANKRD49	219069_at	3.27E-08	1.0953417	KYAT1	206037_at	0.000445	1.2285929
ADGRD1	232267_at	3.34E-08	3.9024869	TMX1	208097_s_at	0.000446	-1.2428327
ECM2	206101_at	3.55E-08	2.6617568	PLA2G7	206214_at	0.000446	1.5641414
SLC38A1	218237_s_at	3.69E-08	-2.4915589	NT5E	1553994_at	0.000447	2.0870812
VSIG2	228232_s_at	3.75E-08	2.9465607	CDKN3	1555758_a_at	0.000448	-1.9046962
SDC1	201287_s_at	3.75E-08	-2.2184597	PSMG3-AS1	1561666_a_at	0.000448	1.6064878
FAM13C	1554547_at	3.75E-08	2.6534262	SNU13	230443_at	0.000449	1.5331112
ZNF45	222028_at	3.82E-08	1.1575962	LOC100507520	236553_at	0.00045	1.6366237
CDO1	204154_at	3.87E-08	2.9084191	CFLAR	211317_s_at	0.00045	-1.3114974

ZNF302	228393_s_at	3.87E-08	2.0928759	AK2	208967_s_at	0.00045	-1.0987469
SYNCRIP	217834_s_at	3.9E-08	-1.4241423	LINC01122	1557472_a_at	0.000451	2.072767
ZNF706	227132_at	3.97E-08	1.4638711	CYP4V2	228391_at	0.000453	1.4628103
SEC62	1552790_a_at	3.98E-08	1.5220715	FUT3	214088_s_at	0.000453	-1.6424819
AGO2	225827_at	4.14E-08	-1.4406469	NUP88	214192_at	0.000455	2.0215017
TMEM261	224860_at	4.15E-08	1.4948625	TMED4	1558487_a_at	0.000456	1.0052177
N4BP2L1	243843_at	4.15E-08	1.6733513	CA11	209726_at	0.000459	1.3933744
SMAD5	225223_at	4.15E-08	1.1505766	RPS6KA5	204633_s_at	0.000462	1.5292484
SORT1	224818_at	4.28E-08	-1.7799441	ECHDC2	218552_at	0.000462	1.0705268
DBNDD1	222234_s_at	4.28E-08	-2.0880292	HNRNPR	208765_s_at	0.000463	-1.2633238
TRPC1	205803_s_at	4.36E-08	2.72232	PCNT	233387_s_at	0.000466	-1.9659452
GLT8D2	227070_at	4.4E-08	2.7584961	SNCA	236081_at	0.000466	1.8721362
BUB1	209642_at	4.4E-08	-2.435243	RNF213	225929_s_at	0.000469	-1.2735653
ATF2	212984_at	4.45E-08	1.108895	CHDH	1559591_s_at	0.00047	1.0346114
ABRACL	223361_at	4.47E-08	-2.0481112	LOC400622	1556435_at	0.000472	1.9014696
SHPRH	226366_at	4.55E-08	1.3510989	HCG27	1559050_at	0.000473	1.5711301
MGAT4B	220189_s_at	4.55E-08	-1.2178586	PFDN5	207132_x_at	0.000476	1.1910547
PRICKLE1	232811_x_at	4.63E-08	1.5980615	GAPDH	AFFX-HUMGAPDH/M33197_5_at	0.000479	-2.0775094
DDX3X	212514_x_at	4.86E-08	-1.3712513	HEY1	44783_s_at	0.000479	-1.9672539
CUL3	201372_s_at	4.94E-08	2.1456792	PCAT6	231233_at	0.000481	-1.2551533
TM9SF3	224755_at	4.94E-08	1.3828358	ITFG1	227191_at	0.000482	1.0806071
GPSM2	205240_at	0.00000005	-2.7658587	MYO10	216222_s_at	0.000482	-1.3133596
SENP6	202319_at	5.04E-08	1.2719041	ZSCAN12	206507_at	0.000485	1.2751708
TMEM165	226825_s_at	5.06E-08	-1.8257138	CHRD	211248_s_at	0.000486	1.2807376
SORT1	212807_s_at	5.06E-08	-1.6568927	ADAM33	233868_x_at	0.000489	1.2283195

ZNF334	220022_at	5.15E-08	3.3840286	CKAP2	218252_at	0.000489	-1.1308952
BIVM	222761_at	5.16E-08	1.1546993	BBIP1	228993_s_at	0.000489	1.102662
FAM169A	213954_at	5.24E-08	2.5912712	TMEM98	223170_at	0.000489	1.6111958
IDE	203328_x_at	5.36E-08	-1.04388	GINS1	206102_at	0.000489	-1.3881104
WNK1	211994_at	5.36E-08	-1.4079199	KIAA1683	223600_s_at	0.000489	2.2881579
PPM1M	226074_at	5.6E-08	1.6798141	SLC25A27	1554161_at	0.000489	1.802867
TSTA3	36936_at	5.76E-08	-1.1545809	PLXDC1	219700_at	0.00049	1.2792057
ENO1	201231_s_at	5.95E-08	-2.2653089	ANAPC10	241959_at	0.000491	1.1241296
LOC100506718	204359_at	6.23E-08	2.8766428	CIRBP	225191_at	0.000492	1.3830675
RNASEH1	218497_s_at	6.4E-08	-1.1969957	NONO	208698_s_at	0.000495	-1.0318012
ACAP2	212476_at	6.42E-08	1.2943962	ABI3BP	220518_at	0.0005	1.9187809
TMEM189-UBE2V1	201001_s_at	6.42E-08	-1.5004755	RAPGEF4	205651_x_at	0.000501	1.1228196
LY75-CD302	203799_at	6.51E-08	1.9647144	CD44	209835_x_at	0.000505	-1.2149522
CENPF	209172_s_at	6.51E-08	-2.4691285	GGTA1P	228376_at	0.000505	1.7148972
RORB	242385_at	6.82E-08	3.3374976	FLJ46875	230203_at	0.000506	1.1008833
VDAC1	217140_s_at	7.14E-08	-1.7946538	STK24-AS1	232839_at	0.000507	1.7731761
CTGF	209101_at	7.19E-08	2.5899092	RGS5	218353_at	0.000507	-1.512931
C2orf68	65472_at	7.33E-08	1.2906333	LOC101929340	233527_at	0.000508	2.027184
EIF4G3	201935_s_at	7.45E-08	-1.2983835	UBA6	222600_s_at	0.00051	-1.088514
PHKB	238601_at	7.51E-08	1.1635578	FOXP1	235444_at	0.000512	1.1235494
OXR1	222553_x_at	7.51E-08	1.4577722	IGHA2	217022_s_at	0.000512	-4.7032567
KIF14	206364_at	7.51E-08	-2.1117551	PRKCA	213093_at	0.000512	1.5745583
SLC6A2	215715_at	7.51E-08	5.1959062	BNC2	235723_at	0.000513	1.9087883
PHTF1	210191_s_at	7.51E-08	-1.3288121	FCHSD1	226699_at	0.000513	1.5121924
NMT2	215743_at	7.56E-08	1.9886433	PARP8	244008_at	0.000513	1.2103979
GLUL	217202_s_at	7.6E-08	-1.6229203	WFDC5	242204_at	0.000513	-1.7618671

ARF3	211622_s_at	7.69E-08	-2.6157373	SLC6A2	239394_at	0.000514	1.6569777
ANGPTL1	239183_at	7.8E-08	4.3133463	NBL1	201621_at	0.000514	1.8719185
TM2D1	213883_s_at	7.8E-08	1.1048225	ERCC6L	219650_at	0.000514	-1.9268863
C11orf58	225811_at	7.8E-08	1.095085	ETV5	230102_at	0.000515	2.5785345
NTN5	1553890_s_at	7.85E-08	2.5899009	ADGRF1	235988_at	0.000515	-2.6085147
ZC2HC1A	241808_at	7.87E-08	1.5251901	RAB35	225620_at	0.000515	-1.0328163
AGPS	205401_at	7.89E-08	-1.387835	FREM1	236031_x_at	0.000516	1.7022309
MFSD8	228282_at	8.16E-08	1.317	HOXA11	213823_at	0.000516	1.1172259
SULF1	212353_at	8.41E-08	-2.4905636	DPY19L2P2	215143_at	0.000519	1.625622
ANO4	236420_s_at	8.43E-08	3.1427206	EWSR1	210012_s_at	0.000521	-2.2058922
ARMCX4	1552327_at	8.67E-08	4.1221395	MPP6	205429_s_at	0.000522	-1.812983
ID2B	213931_at	8.88E-08	2.421356	GAD1	206669_at	0.000522	-2.6713601
RAD54L	204558_at	8.93E-08	-2.1441536	KLF15	231015_at	0.000523	1.2497495
HSPB11	214163_at	8.94E-08	1.7404568	IQCA1	238584_at	0.000527	3.7741439
NCAPG	218662_s_at	8.94E-08	-2.497846	ZSCAN30	1557105_a_at	0.000527	1.5169716
SIK1	208078_s_at	8.94E-08	2.2293946	ABI2	209856_x_at	0.000527	-1.44321
ZFPM2	219778_at	9.2E-08	3.8618577	RADIL	223693_s_at	0.000529	1.3319193
OGN	222722_at	9.28E-08	5.3421623	LCAT	204428_s_at	0.000529	2.328854
PHLDA2	209803_s_at	9.75E-08	-2.9446989	TRIM74	1554182_at	0.000529	1.6915245
E2F7	228033_at	9.82E-08	-2.9243209	C1GALT1C1L	241710_at	0.00053	2.1910833
H2AFY	214501_s_at	9.83E-08	-1.0877781	IRAK1BP1	1557174_a_at	0.000533	1.0365461
PDE7A	224046_s_at	9.83E-08	-1.7904882	CFC1B	223753_s_at	0.000533	2.0637897
DTL	222680_s_at	9.83E-08	-1.8944308	CHKB	204193_at	0.000536	1.0375375
ZNF658	231950_at	9.91E-08	1.3343434	RGS5	209070_s_at	0.000539	-1.8129192
LOC101060391	241535_at	1.02E-07	3.9888057	ANP32E	208103_s_at	0.000539	-1.1470964
ZNF582-AS1	231260_at	1.02E-07	3.4579431	DLX6	242940_x_at	0.000541	2.2007178
CEP68	212675_s_at	1.03E-07	1.6280438	PDE8A	1552931_a_at	0.000543	-1.225222

GSK3B	209945_s_at	1.04E-07	-1.0265529	PLAUR	214866_at	0.000545	-1.4079972
PDIA3	208612_at	1.05E-07	-1.6339159	PRORSD1P	237291_at	0.000545	1.2123075
DFFA	223518_at	1.08E-07	-1.1561609	F3	204363_at	0.000545	1.940533
SCARNA13	244786_at	1.09E-07	2.0260806	ZNF337	37860_at	0.000545	1.0109896
PIAS2	214442_s_at	0.00000011	-1.3169179	LOC105377371	1559103_s_at	0.000546	1.2291581
ACVR2A	228416_at	1.11E-07	1.4900835	SPAG1	210117_at	0.000547	-1.2490933
SFXN2	237334_at	1.12E-07	1.8101693	GANAB	211934_x_at	0.000547	-1.0735583
GTF2H2B	230177_at	1.14E-07	1.563253	NUDT16P1	1568593_a_at	0.000548	2.1596413
STAT3	208992_s_at	1.14E-07	-2.2802235	ME2	210153_s_at	0.000549	-1.674012
LOC101927391	244116_at	1.16E-07	2.3353833	ZDHHC15	1552557_a_at	0.00055	1.9599787
GADD45B	209304_x_at	0.00000012	2.0339867	DIAPH3	232596_at	0.000552	-2.3951854
CECR2	239752_at	0.00000012	4.8000434	BRCA2	208368_s_at	0.000552	-1.4597469
FLRT1	210414_at	1.23E-07	2.1956372	SCARA3	223843_at	0.000553	2.3880702
YTHDC1	240459_at	1.23E-07	1.2797276	LXN	218729_at	0.000555	1.2877215
NAALAD2	222161_at	1.26E-07	3.7140078	RORC	228806_at	0.000555	2.2952048
HSPA4	211015_s_at	1.26E-07	-1.5564017	DPH6	238744_at	0.000555	1.1611017
ZWILCH	218349_s_at	1.29E-07	-2.3269635	LOC100129518	215078_at	0.000559	-2.11387
ACTR2	200728_at	1.31E-07	-1.5621152	BNC2	229942_at	0.000561	1.8232944
MIR6741	231715_s_at	1.32E-07	-1.5689976	LDLR	202067_s_at	0.000563	-1.8249334
FLT1	226497_s_at	1.36E-07	-2.0382103	TNFAIP3	202643_s_at	0.000564	-1.1883584
LOC100507547	230054_at	1.38E-07	1.7815611	TTC12	244571_s_at	0.000564	1.2638119
EIF5B	201027_s_at	1.39E-07	-1.3262655	TBX2-AS1	228978_at	0.000564	1.9761373
AKT1	207163_s_at	1.39E-07	-1.3012129	CEP152	238535_at	0.000564	-1.4834069
HEXIM2	243796_at	1.41E-07	1.5099879	RPL34	200026_at	0.000565	1.1707983
LOC100505851	1568648_a_at	1.43E-07	3.68373	BICDL1	228320_x_at	0.000566	-1.3619635
CCBE1	229641_at	0.00000015	3.4480673	MTFR1	203207_s_at	0.000571	-1.2147412
CALM3	209563_x_at	1.51E-07	-1.198842	THBS1	215775_at	0.000572	1.1091982

GPSM2	221922_at	1.51E-07	-1.7218897	EVC	219432_at	0.000573	2.086216
SMARCA4	215714_s_at	1.54E-07	-1.6471815	GSAP	222150_s_at	0.000574	1.4533722
OGN	218730_s_at	1.58E-07	4.3407836	CALB1	205625_s_at	0.000574	-2.4243094
P2RY1	231925_at	1.62E-07	2.3331717	HIST1H4H	232035_at	0.000578	-1.6880769
POLI	238992_at	1.63E-07	1.4830246	WDR19	232163_at	0.000581	1.3299222
ARHGAP20	228368_at	1.71E-07	3.1961628	ANTXR1	220092_s_at	0.000581	-1.7845868
POLR3H	225682_s_at	1.72E-07	-1.4902994	LOC101928725	1560827_at	0.000583	1.0440442
TCTE3	1557945_at	1.72E-07	1.1619554	SUGT1	244368_x_at	0.000583	1.2179915
VDR	204255_s_at	1.75E-07	-1.8963002	HMOX1	203665_at	0.000584	-1.1340912
LTBP4	204442_x_at	1.76E-07	2.9901457	CXCL11	210163_at	0.000586	-2.0578324
E2F3	203693_s_at	1.82E-07	-1.597371	WDR92	235071_at	0.000587	-1.2667869
TRMT13	219130_at	1.84E-07	1.0615029	FAT4	219427_at	0.000592	1.2785453
RTCA	203594_at	1.85E-07	-1.0057048	ATP10D	213238_at	0.000597	1.3755748
ATP8B3	239457_at	1.88E-07	3.5592955	USP7	230967_s_at	0.000597	1.5807974
SSBP2	238484_s_at	1.88E-07	1.9924907	PCDH7	210273_at	0.000597	1.3965843
CHD2	228999_at	1.93E-07	1.3014088	GSTM1	215333_x_at	0.000597	1.2275744
MIR6883	244677_at	1.95E-07	2.6273639	THRA	204100_at	0.000597	1.6186184
TMEM147-AS1	222280_at	1.98E-07	1.253375	ARMCX4	227444_at	0.0006	2.5028271
RAB10	222981_s_at	1.98E-07	-1.2309187	PRRX1	238852_at	0.0006	2.1783079
ICE2	228960_at	2.03E-07	1.1797242	ARSJ	219973_at	0.0006	2.1621606
FUT9	214046_at	2.04E-07	4.1300326	DUSP10	221563_at	0.0006	-1.1668847
NAV1	224773_at	2.04E-07	-2.5931966	FBXL19	228277_at	0.000601	-1.5198374
HNRNPA3	211929_at	2.11E-07	1.0837366	DLG1	202516_s_at	0.000601	-1.0150885
ZMAT1	226344_at	2.11E-07	2.8161189	RPL32P3	1558969_a_at	0.000602	1.3437285
WHSC1	209053_s_at	2.11E-07	-1.4544946	ZNF146	1554433_a_at	0.000602	-1.6285649
CD46	211574_s_at	2.14E-07	-1.9345802	CALD1	205525_at	0.000603	1.1177884
FAM175A	226521_s_at	2.15E-07	1.4172923	ADH1B	209612_s_at	0.000604	2.4289053

CCNJ	229091_s_at	2.15E-07	-1.2518907	EPB41L4A-AS1	225698_at	0.000604	1.3032
SLC24A3	57588_at	2.15E-07	2.4295735	MBNL2	205018_s_at	0.000615	-2.1054626
RFNG	212968_at	2.18E-07	1.306514	TAF13	205966_at	0.000615	-1.9589459
CCNB2	202705_at	2.21E-07	-2.4628019	GSAP	213142_x_at	0.000615	1.4327745
KLF3-AS1	219871_at	2.25E-07	3.1121334	DPP6	228546_at	0.000621	2.8846832
FBXL18	227500_at	2.31E-07	-1.979891	GTSE1	211040_x_at	0.000621	-1.1352443
EXOSC8	215136_s_at	2.33E-07	1.0620711	NR3C1	211671_s_at	0.000625	1.3513705
YTHDF2	222430_s_at	2.53E-07	-1.0276185	NIN	234299_s_at	0.000626	-1.1660154
EGLN1	221497_x_at	2.55E-07	-1.2919701	LGALS8	210732_s_at	0.000628	-1.4108535
ZNF550	228099_at	2.56E-07	1.1158189	ITIH4	37201_at	0.000629	1.6026321
ESM1	208394_x_at	2.59E-07	-2.2977161	BICD1	231964_at	0.00063	1.2578788
HSPH1	206976_s_at	2.62E-07	-1.2301866	PALMD	218736_s_at	0.000637	1.5902399
FUBP1	214093_s_at	2.62E-07	1.395237	FOXN3	230790_x_at	0.000641	1.1703325
PAXIP1-AS1	235587_at	2.62E-07	1.2480463	PDK1	206686_at	0.000641	-1.616714
TTC17	224849_at	2.62E-07	1.1651288	TBC1D8B	238067_at	0.000648	2.2742827
MND1	223700_at	2.63E-07	-2.7045173	RFFL	237919_at	0.000648	-1.0659961
P4HB	200656_s_at	2.64E-07	-1.3606885	SFT2D3	229141_at	0.000649	1.0120128
PLSCR4	218901_at	2.64E-07	2.0402885	SLC27A2	205769_at	0.000651	-1.5615582
STMN3	222557_at	2.65E-07	2.0990431	ZNF578	1562245_a_at	0.000653	2.6187098
PRKAR1A	200604_s_at	2.66E-07	-1.7176621	C12orf57	224719_s_at	0.000653	1.3824971
LOC100272217	243785_at	0.00000027	1.8681492	LOC101928954	235826_at	0.000654	1.0114957
PTPRF	200637_s_at	2.73E-07	-1.9799798	CYAT1	1558176_at	0.000655	1.4513937
ZNF33B	1558586_at	2.73E-07	1.4350387	DDX19B	230974_at	0.000655	1.5433964
SIDT2	56256_at	2.74E-07	1.23424	FBXO5	234863_x_at	0.000657	-1.6226512
SMAD9	227719_at	2.76E-07	2.8759943	LOC100130417	1555980_a_at	0.000657	1.983662
EPPK1	232165_at	2.79E-07	-2.729425	ZNF268	238030_at	0.000658	1.0334701
ZNF837	1556822_s_at	2.82E-07	2.9210073	YWHAZ	200640_at	0.00066	-1.3845926

KIAA1210	232332_at	2.82E-07	3.4802189	PLPP7	224506_s_at	0.000662	1.6979672
ARMCX4	207799_x_at	2.85E-07	2.5498927	ID4	229386_at	0.000664	2.0059147
SHCBP1	219493_at	0.00000029	-2.2367212	TICRR	230021_at	0.000668	-1.9222059
ITM2A	202746_at	2.91E-07	2.1549515	CDK10	203469_s_at	0.000669	1.3480959
ZMYM5	235620_x_at	2.93E-07	1.1852588	LYPLAL1	230174_at	0.000669	2.4607851
DTWD1	219291_at	2.93E-07	1.0907148	PRKCQ-AS1	232001_at	0.000669	2.0574208
RBBP6	1552329_at	2.94E-07	1.2484089	PFN1	200634_at	0.000672	-1.1054215
MKLN1	204423_at	2.98E-07	-1.1982429	SLC2A8	239426_at	0.000672	1.2156083
MICU3	238458_at	2.99E-07	3.3505054	GBP1	231578_at	0.000674	1.497975
GATS	1553971_a_at	2.99E-07	1.4870557	ZXDC	218639_s_at	0.000675	1.1819533
TNXB	206093_x_at	2.99E-07	2.5506403	RGS16	209325_s_at	0.000679	-1.5931593
SHC2	213464_at	0.0000003	1.8830371	FKBP5	224856_at	0.000679	-1.1811478
PSMC2	201068_s_at	3.02E-07	-1.0564261	TMX2-CTNND1	1557944_s_at	0.000681	-1.0531239
TPTEP1	243952_at	3.06E-07	2.8242554	GDI2	200008_s_at	0.000685	-1.1332401
IL13RA1	201888_s_at	3.11E-07	-1.4925874	PABPC1L	231838_at	0.000686	1.4528674
WDR27	228326_at	3.14E-07	2.0922109	LOC100505555	1569157_s_at	0.000687	1.3510041
PEG3-AS1	230068_s_at	3.24E-07	3.2647457	ICMT	201609_x_at	0.000687	-1.3153471
L3MBTL1	210306_at	3.24E-07	1.5936778	LOC101928269	209359_x_at	0.000689	-1.5101358
NPHP3	235410_at	3.31E-07	1.8187018	CENPU	218883_s_at	0.000695	-1.0650161
ARF3	200734_s_at	3.31E-07	-1.4215559	RNF207	1555870_at	0.000696	1.1741866
LINC-PINT	228702_at	3.34E-07	2.1794613	HSPA6	213418_at	0.000699	-1.2638335
FAM83D	225687_at	3.34E-07	-2.6373408	TNFSF10	202688_at	0.000699	1.5506387
LINC00667	215283_at	3.35E-07	1.9299746	CEP85	227818_at	0.000699	-1.1396924
GALT	203179_at	3.35E-07	1.1857982	FAM218A	1553710_at	0.0007	2.0487547
SCD	200832_s_at	0.00000035	-2.1723623	SLC7A3	230597_at	0.000703	1.1476635
ARPC2	208679_s_at	3.56E-07	-1.5461602	GSTT2	205439_at	0.000703	2.2901967
CDC6	203967_at	3.56E-07	-1.9585289	BIRC5	202094_at	0.000711	-1.5387223

PATL1	225468_at	3.75E-07	-1.5114189	C9orf72	1553133_at	0.000713	1.8204671
PLA2G6	204691_x_at	3.78E-07	1.2404685	TFCP2L1	219735_s_at	0.000713	-2.0840586
KIAA0101	202503_s_at	3.78E-07	-2.159653	CEACAM6	203757_s_at	0.000713	-2.9301804
CASP9	203984_s_at	3.89E-07	1.1485911	LSM8	228529_at	0.000722	1.1515199
SYAP1	225154_at	3.93E-07	-1.3964524	LINC00526	229829_at	0.000722	1.3341415
TSTA3	201644_at	3.93E-07	-1.2172098	PLN	204939_s_at	0.000722	2.4719517
ISG15	205483_s_at	3.96E-07	-2.4678256	ZSWIM7	228719_at	0.000723	1.5164829
MTHFD2	201761_at	4.05E-07	-1.8101222	CCDC148	231504_at	0.000724	2.0195621
ALOX12-AS1	229833_at	4.05E-07	1.3284454	USP9X	230543_at	0.000725	-1.4493442
BIRC5	202095_s_at	4.06E-07	-2.3573047	HOXD11	214604_at	0.000726	1.2369794
LEPROT	227095_at	4.15E-07	1.2148585	UQCRC2	241755_at	0.000727	1.2291368
FAM76B	226753_at	4.19E-07	1.0191537	ZNF404	239043_at	0.000729	2.4185953
NIPBL	207108_s_at	4.19E-07	-2.0523997	ABCA1	203504_s_at	0.00073	-1.1188535
FBXL4	236994_at	4.19E-07	1.317314	GLIPR1	214085_x_at	0.00073	1.2149673
NEDD4L	212445_s_at	4.19E-07	-1.8582647	TNFAIP8L1	227420_at	0.000734	-1.2759976
KIF11	204444_at	4.23E-07	-2.2948345	ANGPT2	211148_s_at	0.000738	-1.4215716
LOC101928269	209360_s_at	4.24E-07	-2.3450739	SIPA1L2	225056_at	0.000738	-1.2045053
MYO15B	59375_at	4.26E-07	1.6049249	PMP22	210139_s_at	0.000742	1.641613
METTL14	241689_at	4.29E-07	1.1281397	DFNB59	235365_at	0.000743	1.2476882
PRR11	228273_at	4.32E-07	-2.2814136	DOCK5	1570078_a_at	0.000745	2.3279989
BRE-AS1	1568768_s_at	0.00000044	1.9880863	CD58	216942_s_at	0.000745	-1.0146165
FGD6	226799_at	4.42E-07	-1.8095048	SARM1	213257_at	0.000746	1.1172456
LOC105369477	1558512_at	4.43E-07	1.1926303	DGKH	216349_at	0.000746	-1.4357213
SSB	201138_s_at	4.45E-07	-1.1279244	ASF1A	203427_at	0.000749	1.1639186
FNDC5	226096_at	4.53E-07	3.0101699	GPCPD1	230492_s_at	0.00075	-1.1948481
ZNF34	219801_at	4.58E-07	1.2170125	KNL1	1552682_a_at	0.000753	-1.5973333
MIR424	228235_at	0.00000046	2.8566308	KATNAL1	223790_at	0.000753	-1.2884282

CLDN8	214598_at	0.00000046	3.2975393	C4orf48	229860_x_at	0.000754	-1.1924598
SPP1	209875_s_at	4.66E-07	-2.6531999	FCER1G	1554899_s_at	0.000755	-1.9247916
LOC101928728	238094_at	4.74E-07	2.892016	MIR4458	236591_at	0.000762	1.5194209
CIRBP	228519_x_at	4.76E-07	1.7191053	LGR5	210393_at	0.000765	-2.2831052
CDCA8	221520_s_at	4.76E-07	-2.6742182	MATN1-AS1	1557557_at	0.000765	1.7002465
SRRM4	230010_at	4.82E-07	2.4378865	IHH	215420_at	0.000765	1.7450133
XPNPEP1	208453_s_at	4.84E-07	-1.1797209	LINC00515	1556414_at	0.000765	1.0751093
TIAL1	230350_at	4.86E-07	1.0925183	RASSF8	225946_at	0.00077	1.6247273
CREBRF	235556_at	4.87E-07	1.1640758	TFAP2A	204653_at	0.000774	-2.7961473
FAM226B	242292_at	5.05E-07	2.9816948	SRPRB	222532_at	0.000777	-1.1173172
IL11RA	204773_at	0.00000051	2.08005	TMEM220	229693_at	0.000777	1.2183306
CXCL12	209687_at	0.00000051	3.7258807	NTRK3	213960_at	0.000779	2.2989037
SARNP	229069_at	5.15E-07	1.0574405	PLIN5	241368_at	0.000781	1.2349229
TMEM132B	236824_at	5.21E-07	3.0742099	FAM162A	224345_x_at	0.000784	-1.0873471
POC1A	226355_at	5.21E-07	-1.3891426	KLHDC3	208784_s_at	0.000784	-1.0518017
TOP2A	201292_at	5.27E-07	-2.1613931	FBN1	202766_s_at	0.000784	1.6748978
APPBP2	202630_at	5.33E-07	1.1349732	PKDCC	225380_at	0.000785	1.9629071
DIO3OS	223757_at	5.42E-07	3.9164176	HOXD10	238847_at	0.000791	2.1437155
SPC24	235572_at	5.47E-07	-2.5187311	FOXC1	1553613_s_at	0.000792	-2.6085799
CTSF	203657_s_at	5.64E-07	2.3226187	FGF2	204422_s_at	0.000794	1.8176176
KDM6A	203991_s_at	5.66E-07	1.0896603	EIF1B	201738_at	0.000795	1.0062419
STAT1	AFFX-HUMISGF3A/M97935_MA_at	5.66E-07	-2.3142785	ENGASE	220349_s_at	0.000796	1.2123908
TLCD2	241359_at	5.72E-07	-1.4014367	ZNF527	244861_at	0.000797	1.3484272
RCC2	224578_at	5.83E-07	-1.0335737	MIR4296	1561963_at	0.000797	-1.6258315

SGPP2	244780_at	5.85E-07	-2.0790735	SGPP2	238567_at	0.000797	-1.4515197
APEX2	204408_at	5.98E-07	-1.4058216	VWA1	222723_at	0.000798	-1.6251103
TIMELESS	203046_s_at	5.98E-07	-1.1976575	SPX	229778_at	0.000798	2.4999921
TRAF3IP2-AS1	230589_at	5.98E-07	3.2707997	HCN1	1556351_at	0.000798	2.3624713
PDGFD	219304_s_at	5.98E-07	2.0812667	INTS7	222250_s_at	0.000799	-1.0381725
FBXL16	227641_at	0.0000006	1.8951658	MFSD6	225325_at	0.000803	-1.0552571
MKI67	212023_s_at	6.05E-07	-2.4313329	ZNF703	230586_s_at	0.000808	1.874042
CXorf57	219355_at	6.07E-07	3.497661	PDGFRA	215305_at	0.000808	1.9907817
PTPRF	200635_s_at	6.13E-07	-1.2590672	MAGED2	208682_s_at	0.00081	1.13168
PBK	219148_at	6.18E-07	-2.6892589	SQSTM1	213112_s_at	0.000812	-1.5786288
CCNYL1	228810_at	6.21E-07	-1.5645604	EXOC7	212026_s_at	0.000814	1.1959316
CCNJ	219470_x_at	6.22E-07	-1.2388563	ZADH2	243417_at	0.000816	1.2625892
SNX4	205329_s_at	6.36E-07	-1.6393733	PCDH7	228640_at	0.000818	2.2498216
NT5E	227486_at	6.36E-07	3.0847941	ANAPC4	226917_s_at	0.000818	1.3405417
NAV1	227584_at	6.39E-07	-2.0356571	PARTICL	238887_at	0.000828	1.0462209
ZNF302	228392_at	6.44E-07	1.8182432	MAP4	200836_s_at	0.000829	-1.2044778
WARS	200628_s_at	6.44E-07	-2.8750709	TMPRSS2	211689_s_at	0.00083	-1.9621398
OAS1	205552_s_at	6.44E-07	-2.9134128	CYP4F12	206539_s_at	0.000833	1.969024
ID4	226933_s_at	6.52E-07	2.6846752	ZNF781	1552785_at	0.000836	1.6200349
KIAA0368	212427_at	6.57E-07	1.2509242	ZNF311	236550_s_at	0.000837	1.8337739
CDCA3	223307_at	6.57E-07	-2.1406858	PAFAH1B2	210160_at	0.000839	-1.0592036
ACADSB	226030_at	0.00000066	1.1815724	SDF2L1	218681_s_at	0.000839	-1.0584304
ILVBL	210624_s_at	6.62E-07	1.1682571	WIF1	204712_at	0.000839	3.9966016
ARPC1B	201954_at	6.67E-07	-1.4505694	CLU	222043_at	0.000844	1.999945
TMEM132B	236468_at	6.67E-07	2.1699416	MSI2	225238_at	0.000846	-1.4641937
RAB27A	209514_s_at	6.67E-07	-1.3011628	ZNF559	224518_s_at	0.000849	1.1328851
CNRIP1	226751_at	6.67E-07	1.9681235	RPARP-AS1	213964_x_at	0.00085	2.246616

TNXB	216333_x_at	6.67E-07	2.4286828	ADCY10P1	236832_at	0.000859	1.3455398
USP3	226652_at	6.67E-07	1.0257489	RPS14	208645_s_at	0.00086	1.1430792
ZNF564	1553957_at	0.00000067	1.0269919	L3MBTL4	228557_at	0.000861	1.6994896
MKI67	212021_s_at	0.00000067	-1.7505912	TPM4	1559989_at	0.000867	1.5896247
FBXL3	225132_at	6.84E-07	1.2650898	U2AF1L4	226700_at	0.000869	1.3516177
ROBO3	219550_at	6.95E-07	2.8749091	MCCC2	209624_s_at	0.000874	-1.022336
P2RY14	206637_at	6.95E-07	2.57986	LOC100288637	1558750_a_at	0.000875	-1.410923
MIRLET7D	227793_at	6.97E-07	1.6000072	CCDC88C	215343_at	0.000879	-1.1627247
KRBOX1	228721_at	7.03E-07	1.3732522	ZEB1	212764_at	0.000882	1.5027822
LOC100506548	224763_at	0.00000071	1.5520801	PLCL1	205934_at	0.000884	1.7086073
RRN3P1	216908_x_at	0.00000071	2.069062	MRPL42	222466_s_at	0.000887	-1.0865001
EPOR	37986_at	7.12E-07	1.7757132	RGL1	209568_s_at	0.000887	1.1829506
PLAGL1	207002_s_at	7.14E-07	2.5471154	SAMD8	242062_at	0.000891	-1.2320382
C8orf46	229430_at	7.16E-07	2.7758754	GATS	227321_at	0.000891	1.3922456
ALKBH8	235610_at	7.16E-07	1.0092431	REM1	210300_at	0.000893	1.1207879
C12orf29	213701_at	7.16E-07	1.2317885	MRS2	228542_at	0.000895	-1.1623559
PTBP3	207223_s_at	7.17E-07	-1.4806043	SDHAP2	226693_at	0.000895	1.1082662
STX16	221499_s_at	7.22E-07	1.1330448	APLP2	208702_x_at	0.0009	-1.3473997
SYNPO2	227662_at	0.00000077	2.9488088	AKT2	1560689_s_at	0.0009	1.0046404
PLAUR	211924_s_at	7.83E-07	-2.4045843	HHIPL1	1556564_at	0.000905	-1.6744345
VAV3	218807_at	7.86E-07	-2.4032241	HSF4	210977_s_at	0.000907	1.5609382
EWSR1	210011_s_at	7.88E-07	-1.3725106	NSD1	235760_at	0.000909	-1.2576577
SOCS5	209648_x_at	7.91E-07	1.1457188	STK4	205411_at	0.00091	-1.1751375
NIT1	241395_at	7.93E-07	1.9446789	LPP	241879_at	0.000912	-1.2128752
NAV1	224771_at	8.08E-07	-2.26301	WWC2	222738_at	0.00092	1.0614892
EPC1	238633_at	8.08E-07	1.3548179	LINC00476	239799_at	0.000921	1.2755416
PCNP	217816_s_at	8.13E-07	1.3134892	FKBP5	224840_at	0.000922	-1.7787944

ENTPD7	220153_at	8.16E-07	-1.7301937	LINC00959	229323_at	0.000922	1.4606254
PDS5A	212138_at	8.18E-07	-1.010663	CHCHD7	222701_s_at	0.000922	-1.0327097
SMC4	201663_s_at	8.24E-07	-1.6907363	PPP3R1	204507_s_at	0.000923	-1.4733476
SBK1	226549_at	8.24E-07	-1.9820802	SEC61A1	222385_x_at	0.000923	-1.1372228
TPTE2P6	233675_s_at	8.24E-07	3.5127176	FASTKD2	205976_at	0.000924	1.1052756
CHD4	201183_s_at	8.25E-07	-1.3439908	KANK4	229125_at	0.000924	-3.0903696
PSMF1	201053_s_at	8.28E-07	1.2661836	C5orf24	1553106_at	0.000924	-1.9683511
JAZF1	225798_at	8.32E-07	1.8111538	KLF11	218486_at	0.000926	1.0553655
MAP3K1	225927_at	8.37E-07	1.2617688	CCNF	204827_s_at	0.000926	-2.0193205
PALM2-AKAP2	202760_s_at	8.42E-07	-2.0291096	DLEU2	1556821_x_at	0.000928	-1.7652931
TPR	228709_at	8.52E-07	1.1827859	LAD1	216641_s_at	0.000931	-1.3914215
PIAS1	217862_at	8.57E-07	1.0276497	NABP1	219334_s_at	0.000933	1.3686435
LMOD1	203766_s_at	8.64E-07	3.2035074	GPSM2	230002_at	0.000937	-1.4855776
COPA	208684_at	8.65E-07	-1.408208	FCGR1B	214511_x_at	0.000938	-1.5102752
RAD52	205647_at	8.72E-07	1.7935891	TNXB	208609_s_at	0.000946	1.4191116
SLC6A2	210353_s_at	8.72E-07	3.1876222	CD44	1557905_s_at	0.000946	-1.8428075
ACTR2	200727_s_at	8.72E-07	-1.7498681	MRPS18C	220103_s_at	0.00095	1.8798662
FGF7P3	1554741_s_at	8.74E-07	2.0525513	EPRS	200841_s_at	0.000951	-1.5504432
TMEM53	1554077_a_at	8.74E-07	-1.0220454	ENPP1	229088_at	0.000952	2.1090619
DCAF7	221745_at	8.79E-07	-1.0845211	ZNF83	236429_at	0.000954	-1.2508237
MREG	232682_at	9.01E-07	-1.373053	DNAJB6	209015_s_at	0.000955	-1.1691553
SULF1	212354_at	9.02E-07	-2.2899281	LSAMP	228218_at	0.000956	2.1107701
YKT6	217785_s_at	9.05E-07	-2.4447891	CEACAM6	211657_at	0.000958	-2.2630165
CORO2A	205538_at	9.11E-07	-1.2842706	MEOX2	206201_s_at	0.000958	2.5842797
AIFM2	224461_s_at	9.13E-07	-2.4972234	LRRC75B	228064_at	0.000961	1.2969202
KIF23	204709_s_at	9.26E-07	-2.7616153	VWA1	218731_s_at	0.000965	-1.7555111
HNRNPAB	201277_s_at	0.00000093	-1.6826776	FAM92A1	228011_at	0.000965	1.0584587

CLN5	214252_s_at	9.45E-07	1.1691516	ZDHHC2	244779_at	0.000965	2.2630804
EXD2	229131_at	0.00000095	1.0474385	NREP	230424_at	0.000969	1.1640753
HJURP	218726_at	9.76E-07	-2.1333595	WISP1	229802_at	0.000969	1.3547898
UHRF1	225655_at	9.76E-07	-2.0633408	ZNF254	1559449_a_at	0.000977	-1.4764797
LINC00667	228160_at	9.87E-07	2.5800775	TIGD6	220986_s_at	0.000979	1.0879655
MTERF2	225346_at	9.98E-07	1.1007677	ZFP2	207757_at	0.00098	1.7504082
MINCR	235428_at	0.000001	1.4994104	MXRA7	212509_s_at	0.000982	1.1839055
ANGPTL1	231773_at	0.00000101	4.9077031	SNORD116-21	232976_at	0.000985	2.0299548
NACA	222018_at	0.00000101	1.2173778	DYSF	218660_at	0.000985	-1.2331083
RGS20	1569303_s_at	0.00000101	1.9843507	GABRP	205044_at	0.000985	3.2049273
SERP2	239889_at	0.00000101	3.353669	TRAM1L1	244334_at	0.00099	1.7595604
KDSR	229850_at	0.00000101	1.3116808	OTUD5	1555426_a_at	0.00099	-1.6061054
LINC00909	226924_at	0.00000101	1.2493973	TRA2B	210180_s_at	0.000991	1.0230267
SPC25	209891_at	0.00000102	-2.5393918	ATG16L2	229389_at	0.000991	1.379396
LOC101930115	230721_at	0.00000102	1.2032205	LOC401433	1560109_s_at	0.000991	2.6183715
DHFR	202532_s_at	0.00000102	-1.2456511	TCF19	223274_at	0.000994	-1.0280992
SKA2	225686_at	0.00000103	-1.1350612	KLF16	224111_x_at	0.000994	-1.139266
OGT	229787_s_at	0.00000104	2.0357213	SUCLG2-AS1	1564097_at	0.000997	2.0938038
PDK3	206348_s_at	0.00000104	-2.8542809	MIR6872	203071_at	0.000997	1.4747114
TES	202719_s_at	0.00000105	-1.509918	PPIB	200967_at	0.000998	-1.0692712
SRGAP1	233888_s_at	0.00000107	-2.0007186	SMC1A	1555677_s_at	0.000999	-2.7117058
MPZL1	210594_x_at	0.00000109	-2.1346468	SPATA17	1552269_at	0.001	-1.9754607
FAM222B	229418_at	0.00000109	-1.1243549	VEGFA	211527_x_at	0.001	-1.5872716
ZNF311	236551_at	0.0000011	3.0766455	ZNF559-ZNF177	207417_s_at	0.001	1.0234187
KIF4A	218355_at	0.0000011	-1.8640644	DPT	213068_at	0.00101	2.2339763
DPY30	1555522_s_at	0.00000111	-1.1486912	FBXL22	241350_at	0.00101	1.9578982
VILL	209950_s_at	0.00000111	2.6208065	C1orf21	221272_s_at	0.00101	1.5757743

BAIAP2L1	227372_s_at	0.00000112	-1.446139	CDCA2	236957_at	0.00101	-1.8085883
LOC100128108	243957_at	0.00000113	2.2925036	LINC00886	230780_at	0.00101	1.4413195
AGO1	222576_s_at	0.00000114	-1.6251709	DLEU2	1556820_a_at	0.00102	-1.6634181
ZNF300P1	244289_at	0.00000115	2.6909148	RNF139-AS1	227745_at	0.00102	1.1101005
ABCA8	204719_at	0.00000115	3.6602507	LOC102724275	241804_at	0.00102	1.0500831
PSMD11	208777_s_at	0.00000117	-1.2768267	VDAC3	208846_s_at	0.00102	-1.5113626
ACTR2	1554390_s_at	0.00000118	-1.6031535	MUM1	230461_s_at	0.00102	1.8702885
DEPDC1	222958_s_at	0.0000012	-2.58033	OAF	225510_at	0.00103	1.0104335
TK1	202338_at	0.0000012	-1.6289892	C1orf116	228865_at	0.00103	-1.8907771
BCLAF1	229454_at	0.00000121	1.6199282	SFRP2	223122_s_at	0.00103	-3.0834097
UCA1	227919_at	0.00000121	3.1298563	FDXACB1	229989_at	0.00103	1.0902242
PATL1	235235_s_at	0.00000121	-1.7397431	BORCS7	225334_at	0.00103	1.1743052
CASP6	209790_s_at	0.00000121	-1.3015864	SLITRK6	235976_at	0.00103	3.2089595
DNAJC27	227859_at	0.00000122	1.1699588	LINC00869	1563483_at	0.00103	1.0639469
GALNT2	223991_s_at	0.00000122	-1.3471365	COL1A1	202311_s_at	0.00105	-1.1758938
RORB	231040_at	0.00000122	3.5636366	PLEKHB2	201411_s_at	0.00105	-1.3923882
EIF4G1	208625_s_at	0.00000127	-1.2936295	GADD45B	213560_at	0.00105	1.7783293
ZNF207	228157_at	0.00000128	1.6227165	TRNT1	1552625_a_at	0.00105	-1.2118646
PITPNA-AS1	228972_at	0.00000128	1.3889673	E2F1	2028_s_at	0.00105	-1.0189692
SSBP2	238483_at	0.00000128	1.5680635	XPO5	223056_s_at	0.00105	-1.026384
ID3	207826_s_at	0.0000013	3.0579294	MST1L	215563_s_at	0.00105	1.8398043
LOC101930363	214945_at	0.0000013	2.7929978	LOC101927668	237192_at	0.00105	1.6785618
MTCH2	217772_s_at	0.0000013	-1.4680843	PNKD	233177_s_at	0.00105	-1.268677
ERO1A	225750_at	0.0000013	-1.7420012	ADIG	1554128_at	0.00105	-1.9805053
TSHZ3	223393_s_at	0.0000013	2.3076451	INPP4A	208363_s_at	0.00106	-1.473703
GSTM3	235867_at	0.00000131	1.3605068	TMCO3	220240_s_at	0.00106	-1.443688
ZMYM2	226512_at	0.00000132	1.0726933	LIMS4	223800_s_at	0.00106	2.2640999

GNG11	204115_at	0.00000132	1.8287498	CLN8	222874_s_at	0.00106	-1.2746264
EGR1	201693_s_at	0.00000132	1.9869826	SPG20	236600_at	0.00106	1.3504366
PRDM5	235764_at	0.00000135	2.4580022	LOC101930349	225817_at	0.00106	1.220233
GAS7	202191_s_at	0.00000135	2.2316745	RBM19	206019_at	0.00106	1.0989353
STAM2	215044_s_at	0.00000136	-1.0488333	UXT	218495_at	0.00106	1.0147506
TMEM200B	227386_s_at	0.00000136	1.6634983	ASAH1	1555419_a_at	0.00106	-1.5054537
ITCH	209744_x_at	0.0000014	-1.175806	HSPB8	233057_at	0.00106	1.206199
TBC1D32	232038_at	0.00000141	1.4644579	ZNF677	228974_at	0.00106	2.0431402
SEC22A	238078_at	0.00000142	1.0759458	SPTLC3	236201_at	0.00107	1.1178953
CLIC4	221881_s_at	0.00000143	-2.0692753	LINC00899	1556304_s_at	0.00107	1.4880593
GDF7	214238_at	0.00000145	1.8968373	TOPORS-AS1	221979_at	0.00107	1.6032715
LAYN	228080_at	0.00000145	1.3811611	ST13	208667_s_at	0.00107	1.1228979
CELF6	227775_at	0.00000145	1.9004064	VEGFA	212171_x_at	0.00108	-1.231869
NR3C2	205259_at	0.00000148	2.8548977	NABP1	233085_s_at	0.00108	1.2835382
THBS1	235086_at	0.00000149	2.0133386	MIR1178	242872_at	0.00108	-2.139216
NCOA3	211352_s_at	0.0000015	-1.4853114	PAX5	206802_at	0.00108	1.6983536
CBX3	201091_s_at	0.0000015	-1.0360779	FGFR2	203638_s_at	0.00108	1.5803096
ARPC5L	226914_at	0.0000015	-1.4061603	CD1A	210325_at	0.00108	-1.2666834
MIR24-2	1555847_a_at	0.00000151	1.8060561	STAT1	AFFX-HUMISGF3A/M97935_5_at	0.00109	-1.6971608
KNL1	228323_at	0.00000151	-1.7838874	GPLD1	238752_at	0.00109	1.7414437
CIRBP	200810_s_at	0.00000153	1.1380472	NFIA	1557639_at	0.00109	2.155304
SYNCRIP	1555427_s_at	0.00000153	-1.7546787	LINC00240	1568854_at	0.00109	1.3959373
SPICE1	234995_at	0.00000154	1.1522544	PSAT1	220892_s_at	0.0011	-1.1430535
RAB27A	210951_x_at	0.00000155	-1.3693066	SH3BGRL3	221269_s_at	0.0011	-1.3401084
GGCT	215380_s_at	0.00000155	-1.5068959	TMPRSS4	218960_at	0.0011	-2.4450586

LOC100507577	221833_at	0.00000155	1.8623246	ABCC4	1554918_a_at	0.0011	-1.7766159
ZSCAN26	213218_at	0.00000157	1.0912162	CELF5	232416_at	0.0011	1.3364841
RNASEH2C	227543_at	0.00000157	1.2312116	LOC101928230	230386_at	0.0011	1.1693792
NIN	224304_x_at	0.00000157	-1.3312558	TGFB1I1	209651_at	0.00111	1.187116
SP4	236265_at	0.00000158	1.1124	FAM184A	1558523_at	0.00111	2.4124168
DEPDC1	235545_at	0.00000163	-2.2885186	HS6ST1	225263_at	0.00111	-1.1065127
SFRP4	204052_s_at	0.00000163	5.2696289	SENP7	223444_at	0.00111	1.0473914
DEFB124	1568377_x_at	0.00000163	2.6238139	RPS9	214317_x_at	0.00111	1.237232
DICER1-AS1	229227_at	0.00000164	1.4201202	GPHN	220773_s_at	0.00112	-1.5167066
WDR37	242255_at	0.00000166	1.3470798	NHSL1	231034_s_at	0.00113	-1.6397728
PCED1A	225505_s_at	0.00000167	1.4664292	ANKRD2	221232_s_at	0.00113	-1.674426
ZNF334	238566_at	0.00000169	2.9552257	FGFR3	204379_s_at	0.00113	2.6425129
ACLY	201127_s_at	0.00000169	-1.2662354	TPM4	212481_s_at	0.00113	-1.4061963
C6orf120	221787_at	0.00000169	1.1632436	TMEM116	234133_s_at	0.00113	1.6093176
KRT8	209008_x_at	0.00000169	-1.9185302	EML4	223069_s_at	0.00114	-1.0188445
ATF3	202672_s_at	0.0000017	2.435997	DENND1A	219763_at	0.00114	-1.7739974
E2F8	219990_at	0.0000017	-2.1963371	LINC00667	226235_at	0.00114	1.5993901
IDE	217496_s_at	0.0000017	-1.1653917	LOC101928728	241668_s_at	0.00115	1.7714625
ATOH8	228890_at	0.00000172	1.9981563	PRKAB1	201835_s_at	0.00115	-1.6752786
SFRP4	204051_s_at	0.00000174	3.5086004	TTC28-AS1	1555871_at	0.00115	1.3904244
CYR61	210764_s_at	0.00000176	1.8012144	MIRLET7BHG	232480_at	0.00115	1.4735801
CENPE	205046_at	0.00000177	-2.1904418	PITX1	209587_at	0.00115	-2.2792611
GLIS1	244128_x_at	0.00000177	2.2518132	BGN	201261_x_at	0.00115	-1.3418793
HNRNPDL	209068_at	0.00000177	1.5024206	LOC101929373	1553633_s_at	0.00115	1.2802416
ATP7A	205198_s_at	0.00000178	1.0147827	HSPB1	201841_s_at	0.00115	-1.4601641
SGO2	235425_at	0.0000018	-1.980013	HILPDA	218507_at	0.00115	-1.3755026
TTC33	219421_at	0.00000183	1.1995018	RBM20	238763_at	0.00115	2.2537992

VAV3	218806_s_at	0.00000183	-2.7668406	IARS2	217900_at	0.00115	-1.0749641
CORO1C	221676_s_at	0.00000189	-1.6650276	LINC01023	231046_at	0.00116	1.2347953
TSC22D1	215111_s_at	0.00000191	1.2946957	SMS	202043_s_at	0.00116	-1.1008986
NUF2	223381_at	0.00000192	-2.6303031	DLAT	211150_s_at	0.00116	-1.4916711
C16orf89	229177_at	0.00000192	4.0486819	CNTN3	229831_at	0.00117	3.2322682
SOCS2	203373_at	0.00000192	1.8772964	CLCNKB	1554748_at	0.00117	1.7974019
WNT4	208606_s_at	0.00000195	3.2129217	RUNX1T1	228827_at	0.00118	1.8235587
LOC100507577	221834_at	0.00000197	1.6014438	ANP32A	201043_s_at	0.00118	-2.0638899
SLC52A2	222155_s_at	0.00000197	-1.6816566	KBTBD7	223412_at	0.00118	1.1060503
CDC25B	201853_s_at	0.00000198	-1.5057116	SERPINF1	202283_at	0.00119	1.5159237
MIR1247	239727_at	0.00000199	2.3418626	FAM65C	227654_at	0.00119	1.6464557
SRPX	204955_at	0.00000202	2.1938447	EME1	234465_at	0.00119	-1.263526
HEY2	219743_at	0.00000204	2.7807624	PXN-AS1	227332_at	0.00119	1.5666084
PAM	214620_x_at	0.00000205	2.1699965	CPD	201942_s_at	0.00119	-1.3374515
LOC105377371	1559102_at	0.00000205	1.186856	CDH11	236179_at	0.00121	2.1675586
MXRA8	213422_s_at	0.00000205	1.6527386	ZNHIT6	233005_at	0.00121	1.0364885
GNB1	200744_s_at	0.00000205	-1.3441689	STAT1	209969_s_at	0.00121	-1.2604426
CASC7	1561042_at	0.00000205	-1.5754083	LSM4	202737_s_at	0.00122	-1.0140929
CREBZF	225595_at	0.00000205	1.4679193	CD200	209582_s_at	0.00122	1.5572275
ASPM	232238_at	0.00000205	-2.5451912	PGM5-AS1	231024_at	0.00122	1.0682407
SMARCA4	214728_x_at	0.00000205	-1.0904913	KIZ	228290_at	0.00122	2.1593414
CD164	208653_s_at	0.00000206	-1.6210773	ZDHHC17	212982_at	0.00122	1.0212333
OGFOD1	241739_at	0.00000211	1.5842984	AP2S1	211047_x_at	0.00122	-1.0032506
LOC101928307	1558795_at	0.00000211	2.8955722	SLITRK4	230680_at	0.00123	2.6873611
MAMDC2	228885_at	0.00000212	2.5583155	RBM43	228304_at	0.00123	1.047956
MFAP4	212713_at	0.00000215	2.8704689	GIMAP2	232024_at	0.00124	1.6631988
LAMP3	205569_at	0.00000215	-2.6195872	LOC102725108	1559950_at	0.00125	1.6344406

ESD	215095_at	0.00000216	1.7747845	PRND	222106_at	0.00125	1.2910727
LINC00938	242523_at	0.00000217	1.347716	SLC41A2	223798_at	0.00125	-1.357526
LOC153682	232794_at	0.00000217	1.6147746	MYC	202431_s_at	0.00125	1.2742751
TNFSF10	214329_x_at	0.00000217	2.1746628	LOC285147	236166_at	0.00125	1.5279472
SERP2	228044_at	0.00000217	2.4536706	KRT6B	209126_x_at	0.00126	-1.6703421
NR2F2	229092_at	0.00000217	1.4380063	ARHGEF10	216620_s_at	0.00126	1.0520344
ECHDC1	233124_s_at	0.00000217	-1.4312265	LYRM4	222672_at	0.00126	1.0538488
PTBP3	214697_s_at	0.00000219	-1.6658187	ZNF784	228364_at	0.00126	1.5641713
SYNCRIP	209024_s_at	0.00000223	-1.9483042	TUSC3	235801_at	0.00127	1.6139024
LOC102724851	236353_at	0.00000223	2.1511921	NHSL1	234321_x_at	0.00127	-1.0109617
AMOTL2	203002_at	0.00000224	1.3082874	ICAM1	202637_s_at	0.00127	-1.0126301
IHH	229358_at	0.00000232	4.3652536	SCML1	222747_s_at	0.00128	1.9438121
LOC100129518	215223_s_at	0.00000232	-2.3436892	LPCAT2	222833_at	0.00128	1.3567295
DOCK9-AS2	231062_at	0.00000232	1.1817529	PPP1R3E	229001_at	0.00128	1.035053
NDST2	203916_at	0.00000232	1.0758503	PPM1F	203063_at	0.00128	1.025744
LOC101930115	227351_at	0.00000232	1.3798783	SLC16A11	1552761_at	0.00128	1.3387139
KIF2C	209408_at	0.00000234	-1.7336603	LOC100505555	1569156_at	0.00129	1.8529005
SMAP2	225282_at	0.00000234	-1.2201672	ZC3H14	242443_at	0.0013	1.2615254
DOPEY2	205248_at	0.00000234	-1.7021498	LOC102725271	222020_s_at	0.0013	-1.5215168
EFEMP2	206580_s_at	0.00000235	1.5464534	FBXO9	212991_at	0.00131	1.0869725
PLK4	204887_s_at	0.00000237	-1.8788118	MICALL2	1555862_s_at	0.00131	-2.1440301
FAM228B	230435_at	0.00000248	1.3819564	FZD1	204451_at	0.00132	1.0523702
C1orf21	223126_s_at	0.00000248	1.6242532	INTU	228946_at	0.00132	1.1527866
LOC644656	229870_at	0.00000251	1.3167568	PGD	201118_at	0.00133	-1.4860394
IL21R	237753_at	0.00000252	-2.2504164	CEP44	231850_x_at	0.00133	1.1870588
SAR1A	201543_s_at	0.00000252	-1.2653377	RSL24D1	222465_at	0.00134	1.1935772
SLC6A2	217214_s_at	0.00000252	2.6492902	TXNIP	201010_s_at	0.00134	1.2696234

LOC100289230	238456_at	0.00000252	1.4198529	PABPC5	233136_at	0.00134	2.4726876
GARNL3	223604_at	0.00000254	1.6667608	MYO6	210480_s_at	0.00134	-1.1381373
BRWD1	231960_at	0.00000254	1.078079	SLC7A11	217678_at	0.00135	-1.763204
UBE2F	225783_at	0.00000258	-1.0375604	CES4A	228903_at	0.00135	2.6958528
HIST1H2AM	214481_at	0.00000258	-2.263909	SCP2	201339_s_at	0.00136	-1.0750133
VPS36	222478_at	0.00000261	1.0691737	LOC285097	1556474_a_at	0.00136	2.2940264
ARMCX1	218694_at	0.00000262	2.4366433	FAM46B	229518_at	0.00136	-1.4616926
YBX1	208627_s_at	0.00000262	-2.0658629	NR2F1-AS1	1556696_s_at	0.00137	1.9915474
LIPG	219181_at	0.00000267	-2.9182181	LUM	201744_s_at	0.00137	1.9723885
MYO1B	212365_at	0.00000267	-1.2979436	MOB3B	229568_at	0.00137	1.3901198
SNX21	1553960_at	0.00000272	1.1514171	SLC12A2	225835_at	0.00137	-1.4627969
COL3A1	232458_at	0.00000272	2.7864876	TRAPPC10	1555446_s_at	0.00137	-1.2003355
PCF11	203378_at	0.00000272	1.0333731	GCLC	202922_at	0.00138	1.224278
CEBPG	225527_at	0.00000274	-1.1590524	TCF4	213891_s_at	0.00138	1.3689577
TUBB3	202154_x_at	0.00000276	-1.4131818	PGAP1	241801_at	0.00139	1.5712172
BBS9	37547_at	0.00000278	1.7310316	L2HGDH	224459_at	0.00139	-1.8575079
GALNT2	217788_s_at	0.00000279	-1.3820093	S100A9	203535_at	0.00139	-2.4251165
HECTD2	227568_at	0.00000283	1.6247034	EFEMP2	209356_x_at	0.00139	1.2382464
CAMSAP2	212765_at	0.00000284	1.2022452	EIF1	212225_at	0.00139	1.4639615
PAN2	203117_s_at	0.00000285	1.4066524	SLC35E1	222263_at	0.00139	-1.1900263
EIF2AK1	217736_s_at	0.00000286	-1.198917	MYO10	236718_at	0.0014	-1.320491
NAV1	224772_at	0.00000286	-1.3401239	TFPI	214378_at	0.0014	1.7007607
DIDO1	213213_at	0.00000286	1.0773249	CAMK2D	225019_at	0.0014	1.0598398
TRIM45	219923_at	0.00000291	1.3390771	HIPK2	219028_at	0.00141	-1.093338
USP51	237247_at	0.00000293	2.1918744	PTPN12	216915_s_at	0.00141	-1.7260078
OLFM1	205591_at	0.00000293	2.8866989	IGFBP6	203851_at	0.00141	1.2609782
KLF3-AS1	1570414_x_at	0.00000295	2.187243	SNTB1	226438_at	0.00141	-1.5781783

EIF3M	239848_at	0.00000295	1.5168366	LOC440149	236366_at	0.00142	1.4540296
PRKD1	205880_at	0.00000296	2.1468788	SEMA5A	213169_at	0.00142	1.5026742
AMMECR1	226421_at	0.00000297	-1.5734687	CD44	204490_s_at	0.00142	-1.2910899
GATAD1	214718_at	0.000003	1.1897858	RAB3IP	231399_at	0.00143	-1.1580195
CBL	229010_at	0.00000302	-1.5146055	LMNA	1554600_s_at	0.00143	-1.3700289
PCDH10	1556328_at	0.00000302	3.3005866	IDUA	205059_s_at	0.00144	1.3651835
CYP1B1	202437_s_at	0.00000305	2.389872	COL16A1	204345_at	0.00144	1.3514135
CANX	208853_s_at	0.00000306	-1.2810101	ADAMTS13	223844_at	0.00144	1.3259878
NEURL1B	225355_at	0.00000307	-1.3298868	AAGAB	202852_s_at	0.00144	-1.3674114
RIT1	209882_at	0.0000031	-1.7232027	ZNF333	231369_at	0.00145	1.253269
TTK	204822_at	0.00000311	-2.1387359	SEC62	1552789_at	0.00145	2.0544938
ANLN	1552619_a_at	0.00000312	-2.255245	LINC01139	235599_at	0.00145	2.8550316
LRRN4CL	1556427_s_at	0.00000313	1.9844691	GRK6	211543_s_at	0.00145	-1.3549412
PFKP	201037_at	0.00000314	-1.6419927	HPCAL1	205462_s_at	0.00146	-1.6969923
ADAM33	232570_s_at	0.00000314	2.7977815	FLJ34503	1553448_at	0.00146	1.7382378
KIF18B	222039_at	0.00000316	-1.9589964	NPL	223405_at	0.00147	-1.0497053
FXYD1	205384_at	0.00000316	2.1489167	ALG13	205584_at	0.00148	1.2567719
BDH2	235155_at	0.00000319	1.5764759	CCDC158	243875_at	0.00148	1.7267574
STX6	1552618_at	0.00000319	-1.3349396	ERCC4	210158_at	0.00148	-1.7287093
MRPS25	224873_s_at	0.0000032	1.3919763	FASN	212218_s_at	0.00148	-1.0960733
CPED1	228728_at	0.00000321	2.7260378	DNAH3	1560803_at	0.00148	-1.3122139
LARP4	214155_s_at	0.00000327	-1.0868336	LCA5	229953_x_at	0.00149	1.6509154
GALT	242734_x_at	0.00000336	2.1395685	SCARNA17	240830_at	0.00149	1.3228475
PLEKHH2	227148_at	0.00000336	2.454449	CALU	214845_s_at	0.0015	-1.2767236
PMM1	203467_at	0.00000341	1.2692497	ZBTB46	227329_at	0.0015	1.2628227
PIK3R3	211580_s_at	0.00000342	-2.0392759	SPTBN1	200671_s_at	0.0015	-1.7182152
TMEM132C	232313_at	0.00000346	3.5648427	SVIL-AS1	228786_at	0.00151	-1.1289576

NMT2	205006_s_at	0.00000346	1.4999534	RBM48	221595_at	0.00152	1.2733132
PIK3R3	202743_at	0.00000352	-1.5616312	CTSV	210074_at	0.00152	-2.1920367
TP53BP2	203120_at	0.00000353	-1.2901964	RIPPLY3	207267_s_at	0.00152	-1.8600397
ECM1	209365_s_at	0.00000354	1.6846913	EFNB3	205031_at	0.00152	2.1684585
BAX	208478_s_at	0.00000357	-1.6374898	LRP8	205282_at	0.00153	-1.0041389
RDH10	1552378_s_at	0.00000357	-2.5832883	SLC16A10	219915_s_at	0.00153	-1.3879316
LINC00115	220399_at	0.00000357	1.21548	KLF5	209212_s_at	0.00153	-1.2856957
DYNC2LI1	203763_at	0.00000362	1.0796703	KCNE3	222923_s_at	0.00153	-2.269569
LMNA	203411_s_at	0.00000363	-1.3190297	LARP4	1555384_a_at	0.00153	-1.7570589
RELT	227060_at	0.00000367	-1.6464931	CD44	1565868_at	0.00153	-1.4305821
GALNT2	217787_s_at	0.00000368	-1.98399	CCNE1	213523_at	0.00153	-1.6266998
SLC38A1	224579_at	0.00000368	-2.0361148	TRMT13	232489_at	0.00153	1.1942628
SNORD77	224841_x_at	0.00000369	1.2832333	TPT1-AS1	228913_at	0.00154	1.118423
SENP6	214790_at	0.00000369	1.3935563	RPP25	223415_at	0.00155	-1.1042439
PGAP1	213469_at	0.00000369	1.8842204	ZNF626	235687_at	0.00155	2.2276206
YWHAH	201020_at	0.0000037	-1.220011	CSTA	204971_at	0.00156	-1.7326502
UBE2H	221962_s_at	0.00000371	-1.3796398	LAPTM5	201721_s_at	0.00156	-1.48966
SLC25A27	230624_at	0.00000375	2.017373	MME	203435_s_at	0.00156	2.3455961
METTL10	226634_at	0.00000378	1.1199277	ZNF546	240429_at	0.00156	1.2651807
SRRM4	236151_at	0.00000383	2.1952184	STYX	242408_at	0.00156	-1.1368591
RASGRP3	205801_s_at	0.00000387	-2.1048987	HNRNPL	202072_at	0.00156	-1.4892197
UQCC1	229672_at	0.0000039	1.4650743	GLRB	244680_at	0.00157	1.2311756
RUFY2	241996_at	0.0000039	1.7006038	TEPP	240119_at	0.00157	1.5719232
TMEM245	223007_s_at	0.00000398	1.1878389	FAM184A	220150_s_at	0.00157	1.2375955
UBE2H	222420_s_at	0.000004	-1.2654777	YKT6	217784_at	0.00158	-1.5522672
TSPAN17	225235_at	0.00000401	-1.0887898	ADAMTS5	219935_at	0.00159	1.4817311
CDC25C	205167_s_at	0.00000408	-2.4225975	PTGS1	238669_at	0.00159	1.8755478

ATP6V1D	208899_x_at	0.00000411	-1.0751688	AGPAT3	219723_x_at	0.00159	-1.271369
ACLY	210337_s_at	0.00000415	-1.8597098	KNL1	1552680_a_at	0.00159	-1.3959104
EZR	208621_s_at	0.00000416	-2.481467	CXCL10	204533_at	0.0016	-2.1586476
MYNN	241730_at	0.00000418	1.3230657	LINC00939	216209_at	0.0016	2.0560806
PHACTR2	227947_at	0.00000418	1.0685254	NME1	201577_at	0.0016	-1.2010972
RGL3	215054_at	0.00000423	1.8608213	DPP7	238012_at	0.00161	1.1015734
THBS1	239336_at	0.00000423	2.9701564	ZMYND11	1554158_at	0.00163	-1.4908172
TRIM52-AS1	228664_at	0.00000423	1.8332079	EDNRA	204464_s_at	0.00164	1.4426808
LRFN5	230644_at	0.00000427	3.482685	HOXD10	229400_at	0.00164	1.461519
RBM14-RBM4	235511_at	0.00000437	1.3934115	TMEM221	239128_at	0.00164	1.5360335
LIMK1	204357_s_at	0.00000439	-1.6910095	RRP15	214764_at	0.00165	1.3555133
SCAF4	226082_s_at	0.0000044	-1.1910874	CREBZF	202979_s_at	0.00165	1.1354553
KIFC1	209680_s_at	0.00000441	-1.6566587	C19orf33	223631_s_at	0.00165	1.8976711
ZCWPW1	223992_x_at	0.00000445	2.8275559	CRB1	244403_at	0.00165	2.2011562
RAMP1	204916_at	0.00000447	1.3740051	UBE2V2	209096_at	0.00165	-1.0193198
ASPM	239002_at	0.00000448	-2.1281418	CDH2	203440_at	0.00166	2.6342486
PRNP	201300_s_at	0.00000451	2.232479	NRG2	206879_s_at	0.00166	1.3188145
BEX1	218332_at	0.0000046	4.0211601	SALL1	229273_at	0.00166	1.4314234
SNED1	213488_at	0.00000461	1.720236	TNS2	212494_at	0.00166	1.1776663
MAN2C1	203668_at	0.00000463	1.1978006	HBB	217232_x_at	0.00166	1.414345
HNRNPU	200593_s_at	0.00000466	-1.2782298	ITGA9-AS1	1563635_at	0.00166	1.8550008
ADAMTS9-AS2	233113_at	0.00000475	2.6054419	SLAMF7	219159_s_at	0.00167	-2.0860872
COL4A1	211981_at	0.00000479	-2.2015366	DNAJC21	235001_at	0.00168	-1.3018472
KDM6B	1556067_a_at	0.00000482	-1.8688199	NOS1	239132_at	0.00168	1.9703838
ATP8A2	219659_at	0.00000483	2.8081431	FAM69B	226940_at	0.00168	1.3436189
ZNF548	1553719_s_at	0.00000485	1.1965376	ZBED3	235109_at	0.00169	-1.0245026
TRIP13	204033_at	0.00000485	-1.6716227	LOC100129518	221477_s_at	0.00169	-1.4522595

GNG7	228831_s_at	0.00000487	1.3759075	KLF9	230636_s_at	0.00169	1.5730989
MUTYH	207727_s_at	0.00000491	1.2329239	DUSP10	215501_s_at	0.00169	-1.1386497
RBM24	235004_at	0.00000491	2.7921147	BEND6	231175_at	0.00169	2.1041316
SDC1	201286_at	0.00000499	-1.7721965	PRTFDC1	222803_at	0.00169	1.6487373
RPS6KA5	204635_at	0.00000509	1.5620946	RGS16	209324_s_at	0.0017	-1.5213959
PATL1	235234_at	0.0000051	-1.3478745	ZNF441	1553192_at	0.0017	1.7492814
HECTD2	238803_at	0.0000051	1.9028014	OAS2	204972_at	0.0017	-1.7627525
MOB3B	226844_at	0.0000051	1.5051547	CCL3L3	205114_s_at	0.0017	1.3546895
CNN1	203951_at	0.00000513	2.825615	PLA2R1	235746_s_at	0.00171	2.0614092
RAB8B	226633_at	0.00000514	1.193482	CRELD2	218358_at	0.00171	-1.1290509
ZBED8	220770_s_at	0.00000518	1.1323976	SGSM2	217538_at	0.00171	1.1098412
ESCO2	235178_x_at	0.00000519	-2.7293665	ATP6V0D1	212041_at	0.00172	-1.1668011
MAPRE1	200712_s_at	0.00000524	-1.107733	SSPN	204964_s_at	0.00172	2.0625628
LIN7B	241957_x_at	0.00000525	1.1372224	ASAP3	222236_s_at	0.00172	1.632304
WNK1	211993_at	0.00000529	-1.7065996	TNNI3K	241858_at	0.00172	1.1803958
EIF1AX	201018_at	0.00000533	1.6462397	SLC2A1	201250_s_at	0.00173	-1.1920759
SLC6A10PB	215812_s_at	0.00000537	-1.916969	AK9	1552299_at	0.00173	-1.4352645
RPL31	200962_at	0.00000537	1.4550104	MIR5193	1294_at	0.00173	1.0906792
FBXO9	238472_at	0.00000539	1.2095366	OASL	205660_at	0.00173	-1.3714297
LOC145474	230505_at	0.00000542	-1.7050429	LOC283454	229552_at	0.00175	-1.9488226
DNAJB4	203811_s_at	0.00000544	-2.3825855	LPP	202821_s_at	0.00175	-1.0650304
STIL	205339_at	0.00000544	-1.5062223	ERICH1	1556766_at	0.00175	-1.2877771
PDZRN3	212915_at	0.00000544	2.7508932	MEIS3	228327_x_at	0.00176	1.4554056
SNORD77	224741_x_at	0.00000546	1.2553766	ABCF2	207623_at	0.00176	1.8966132
CDC25A	1555772_a_at	0.00000548	-2.3236789	ENC1	201341_at	0.00176	-1.3413619
LMNB1	203276_at	0.00000549	-1.5914527	LOX	204298_s_at	0.00177	-1.8844651
TUBB4B	213726_x_at	0.00000549	-1.3125449	C7orf31	229146_at	0.00177	1.2435099

NUP50	218294_s_at	0.00000554	-1.2551412	RAP1GDS1	217457_s_at	0.00177	-1.0102829
ERO1A	222646_s_at	0.00000556	-1.851471	TMEM27	223784_at	0.00177	1.2560574
MAT2A	200768_s_at	0.00000563	1.0555288	C3orf33	1554176_a_at	0.00177	1.3024923
EFHD2	222483_at	0.00000575	-1.1238565	AP1M2	218261_at	0.00178	-1.6099141
STK4	211085_s_at	0.00000578	-2.2875922	CDCA3	221436_s_at	0.00178	-1.331749
TIPRL	1554351_a_at	0.00000578	-1.232494	ZNF677	1560562_a_at	0.00178	2.0325881
YPEL3	232077_s_at	0.00000578	2.4023256	MUC1	211695_x_at	0.00179	-1.2338373
DTL	218585_s_at	0.00000579	-1.8851042	GAS6	202177_at	0.00179	2.2683336
EML4	223068_at	0.00000579	-1.0099224	P2RY1	207455_at	0.00179	1.2272423
DFFB	206752_s_at	0.00000579	1.1124872	AMN1	226258_at	0.00179	2.0268629
SPAG5	203145_at	0.00000585	-1.3546966	SLC25A46	226831_at	0.00179	1.2371205
ID2	201566_x_at	0.00000589	1.6142621	SLAMF8	219385_at	0.0018	-1.6021639
KPNB1	208974_x_at	0.0000059	-1.071567	GBP5	229625_at	0.0018	-1.5942017
CDC6	203968_s_at	0.00000601	-1.4053794	CAPN13	234709_at	0.0018	-3.3239078
NUMA1	200747_s_at	0.00000603	1.221144	NHSL1	226490_at	0.00181	-1.1467181
C11orf54	223268_at	0.00000603	1.0658687	CHKA	204233_s_at	0.00181	-1.005907
EZH1	32259_at	0.00000606	1.0343987	LOC283713	230783_at	0.00182	1.5565224
IL1RN	212657_s_at	0.00000607	-2.3565736	KCNC4	235467_s_at	0.00182	1.5222232
PCGF5	227935_s_at	0.00000607	-1.2947765	LGALS4	204272_at	0.00183	2.0980635
TSPOAP1	205839_s_at	0.00000607	1.6017225	AAGAB	202851_at	0.00184	-1.5567749
LPCAT2	227889_at	0.00000612	2.0123165	TGIF2-C20orf24	224376_s_at	0.00184	-1.0089867
DCT	205337_at	0.00000613	3.1222345	ADAM22	213411_at	0.00184	1.4562158
MAPK13	210059_s_at	0.00000614	-1.3157303	DACH1	228915_at	0.00185	2.3896906
JUNB	201473_at	0.00000614	1.5693711	ZNF506	1568720_at	0.00185	1.4592303
ELK4	214831_at	0.00000614	-1.6769693	CTC1	235523_at	0.00185	1.6261313
NCBP2	201521_s_at	0.00000614	-1.3761643	CALML4	221879_at	0.00185	-1.416657
GOT1	208813_at	0.0000063	-1.318428	RUNX1T1	205529_s_at	0.00186	1.9279032

TSPYL5	213122_at	0.0000063	2.1535298	ARSD	223695_s_at	0.00186	1.0751253
LYRM9	213195_at	0.00000633	1.2930123	MGAT4A	219797_at	0.00186	-1.4797646
FLT1	226498_at	0.00000634	-2.9513859	IRS1	204686_at	0.00187	1.8216199
AKIRIN1	222458_s_at	0.00000635	-2.1607407	SMIM10L2A	238697_at	0.00188	1.6564365
NNT	202784_s_at	0.00000637	-1.2763616	SETBP1	227478_at	0.00188	1.1492538
C3orf38	1569129_s_at	0.00000642	1.1351124	OIP5	213599_at	0.00188	-1.8638054
MALL	209373_at	0.00000643	-2.8612934	STARD10	238911_at	0.00188	1.2729497
PRICKLE1	230708_at	0.0000065	2.3217008	TTC3P1	1569472_s_at	0.00189	-1.9818985
FOXL2	220102_at	0.00000652	2.1562951	RNF150	236038_at	0.00191	1.9406835
BLM	205733_at	0.00000653	-1.1604692	GBP5	238581_at	0.00191	-2.1196857
ZNF674-AS1	241972_at	0.00000656	1.4357219	CHURC1-FNTB	1773_at	0.00192	-1.0268683
PPIF	201490_s_at	0.00000656	-2.4163682	ACAD9	239976_at	0.00194	1.023278
EGR1	201694_s_at	0.00000659	1.4920009	HFE2	228621_at	0.00194	-1.3643633
ST14	216905_s_at	0.00000659	-1.5716069	DNAJC3	1558080_s_at	0.00194	-1.1979592
ADAM9	1555326_a_at	0.00000669	-2.2444146	UNC5B	213100_at	0.00194	-1.2929341
HMMR	207165_at	0.0000067	-2.2205264	PAK3	229201_at	0.00194	2.2384697
LOC730268	230588_s_at	0.00000677	1.0676801	MAT2A	200769_s_at	0.00195	-1.0914926
PTPRN2	203030_s_at	0.0000068	1.9384014	ARFGEF2	215931_s_at	0.00195	-1.5152157
GINS4	211767_at	0.00000682	-2.2318176	TMEM55A	226338_at	0.00196	1.5414799
C11orf31	1557801_x_at	0.00000686	1.1260242	GNG11	239942_at	0.00196	1.5350439
S100A11	200660_at	0.00000686	-1.6647077	ZC3H12B	229234_at	0.00196	2.0273815
ATG16L2	225883_at	0.00000687	1.7414995	S100A8	202917_s_at	0.00196	-3.1425969
METTL2A	225253_s_at	0.00000693	-1.3236655	THY1	208850_s_at	0.00196	-1.2066299
TBX3	219682_s_at	0.00000699	2.0361342	CCDC171	236945_at	0.00196	1.442989
LOC100506558	202350_s_at	0.00000707	2.5553929	BRCA1	204531_s_at	0.00197	-1.0653412
CENPT	218148_at	0.0000071	2.4584863	WDR91	230888_at	0.00197	1.1023262
ST8SIA1	1569788_at	0.0000071	2.6485999	FOSL2	225262_at	0.00198	-1.1034453

NUP54	218256_s_at	0.0000071	1.0081385	LOC101928238	1561595_x_at	0.00199	1.6747304
MCOLN3	229797_at	0.00000716	2.2644563	LOC100506990	227917_at	0.002	1.1865441
ZNF709	242028_at	0.00000716	1.1593935	UACA	238868_at	0.002	1.1179163
ETV6	205585_at	0.00000716	-1.3195198	RBPMS	207836_s_at	0.002	1.3711721
PRKAG2-AS1	229157_at	0.00000721	2.2649827	KIAA0895L	1558041_a_at	0.00201	1.2691966
PEX1	204873_at	0.00000726	1.1417243	SNORA70	221989_at	0.00202	1.0591163
LEF1-AS1	243363_at	0.00000728	2.4753028	ATF1	222103_at	0.00203	1.0059831
PCCB	212694_s_at	0.00000736	-1.6264765	CCDC190	1554960_at	0.00203	-1.9019714
C4orf46	201812_s_at	0.00000736	1.4740446	NRXN1	228547_at	0.00203	1.6183477
ABI3BP	223395_at	0.00000736	2.6561764	KIAA1644	52837_at	0.00203	1.4522628
TMEM136	238497_at	0.00000742	1.3384283	CCNL2	222999_s_at	0.00203	1.0101251
KCNH8	1552742_at	0.00000742	2.813586	PAPPA	224941_at	0.00204	1.7087822
NUSAP1	218039_at	0.00000744	-1.9023607	RAD1	228535_at	0.00204	1.0094352
C8orf82	226710_at	0.0000075	1.2453485	TUSC1	227388_at	0.00204	1.5768352
ADM	202912_at	0.0000075	-2.0550167	SVIL	202566_s_at	0.00204	-1.069245
CYBB	203922_s_at	0.00000751	-1.7781015	CLK4	241403_at	0.00204	1.1604658
C11orf31	1557799_at	0.00000754	1.0575426	SYT14	1553654_at	0.00205	2.1794583
FAXDC2	48031_r_at	0.00000754	1.5621446	ZNF783	1556224_a_at	0.00206	1.3309202
ADGRB2	204966_at	0.00000754	-2.1637072	UNC80	230220_at	0.00207	1.6203255
NPL	221210_s_at	0.0000076	-2.0824462	F2RL2	230147_at	0.00207	-2.1925188
EPPK1	232164_s_at	0.0000077	-2.4071259	LINS1	1554455_at	0.00208	1.0049454
CTBP1	203392_s_at	0.00000775	-1.0593727	SPHK2	209857_s_at	0.00208	-1.3821588
RAB33B	221014_s_at	0.00000776	1.1658816	RNF180	236644_at	0.00209	1.9127782
DCN	201893_x_at	0.00000786	2.3522127	GPC4	204983_s_at	0.00209	-2.0148317
SCAMP1	212425_at	0.00000786	1.180622	DLG3	207732_s_at	0.00209	-1.4048269
SPSB3	221769_at	0.00000796	1.5584415	DDX6	204909_at	0.00209	-1.0383044
SCARNA2	237137_at	0.00000796	2.3115212	PYROXD2	228384_s_at	0.00209	1.1963777

TBC1D24	227908_at	0.00000799	-1.1196312	RBM15	1555761_x_at	0.0021	-1.1042292
PID1	219093_at	0.0000081	2.8617978	ZNF502	232247_at	0.0021	1.9421793
KPNA3	221503_s_at	0.0000081	-1.1699429	FKBP4	200894_s_at	0.00213	-1.1348213
PSMD1	201198_s_at	0.00000814	-1.5054123	DPT	213071_at	0.00214	1.6107371
FYB	211794_at	0.00000814	-2.4127193	VIPR2	205946_at	0.00214	1.5565354
ENAH	217820_s_at	0.00000817	-1.10767	SUZ12	1566191_at	0.00214	-1.5502589
TUBB4B	208977_x_at	0.00000818	-1.2737686	KLF3	219657_s_at	0.00215	-1.1297182
PHYKPL	226783_at	0.00000819	1.3273068	ADRA2C	206128_at	0.00215	1.4336213
TUBB3	213476_x_at	0.00000826	-1.3281687	UNK	1562434_at	0.00215	1.1368933
LOC283861	1554520_at	0.00000834	1.6588052	ITM2A	202747_s_at	0.00215	1.2683904
MIR29C	228528_at	0.00000841	2.1487018	LOC102724562	213380_x_at	0.00216	1.4431159
CECR2	223729_at	0.00000841	1.6946974	THUMPD3-AS1	1557357_at	0.00216	1.0868655
ATOH8	1558706_a_at	0.00000842	2.2043612	PLAG1	205372_at	0.00217	1.8290614
SLC6A2	217213_at	0.00000842	2.8152851	GLRA3	216021_s_at	0.00217	-1.5136805
PAX2	206228_at	0.00000842	4.5027399	LINC00667	243426_at	0.00217	1.56895
RRN3P1	215211_at	0.00000842	2.9879728	PCDH17	228863_at	0.00217	-1.4936253
LOC728903	1555872_a_at	0.00000861	1.6128485	CXCL8	202859_x_at	0.00218	-2.3645056
PLCD1	205125_at	0.00000866	1.0926229	CD163	203645_s_at	0.00218	-1.5413066
ZFP36L2	201368_at	0.00000866	1.7325318	LOC101927043	1562604_at	0.00218	1.6540622
THBS1	201110_s_at	0.00000866	2.2993184	ZNF471	230578_at	0.00218	1.6868952
FGF7P3	231031_at	0.00000869	2.2972539	SIAH3	1560676_at	0.00218	2.3747504
MRGPRF-AS1	1560496_at	0.00000869	1.6303798	EIF5B	201026_at	0.00218	-1.0247495
ZFP36	201531_at	0.00000869	1.5632653	TGIF1	203313_s_at	0.00219	1.0106078
EZR	217234_s_at	0.00000869	-2.1626566	SEPT7-AS1	230450_at	0.00219	1.0759859
G2E3	223257_at	0.00000878	1.1244605	LINC00957	1564208_x_at	0.00219	1.9561855
SSPN	226932_at	0.0000088	2.3083245	LINC00092	1562733_at	0.0022	1.0743111
CAST	207467_x_at	0.00000884	-1.0560774	SNED1	213493_at	0.0022	1.9241967

KIAA1217	231807_at	0.00000891	1.263647	PRMT2	228725_x_at	0.0022	-1.087297
ZNF540	238454_at	0.00000898	2.5802117	LOC100996419	1559534_at	0.0022	1.305921
KDM6B	1556066_at	0.00000898	-1.9598874	SPIN3	1554099_a_at	0.0022	1.0638711
KIF26A	234307_s_at	0.00000898	2.3190099	FOXN4	241009_at	0.00221	-1.4874046
RBP7	238066_at	0.00000902	2.4302015	KCNS3	205968_at	0.00223	1.2685034
TUBB	209026_x_at	0.00000902	-1.6242824	ATG4C	228190_at	0.00223	1.1621907
HMMR	209709_s_at	0.00000905	-2.1446149	LOC100996255	234423_x_at	0.00223	1.2583961
NUSAP1	219978_s_at	0.00000906	-1.7816157	FLII	212025_s_at	0.00224	-1.5099105
C12orf66	1554067_at	0.00000907	1.2865033	C8orf88	228107_at	0.00225	1.528019
NR3C1	216321_s_at	0.00000908	1.850732	CAMKMT	1554875_at	0.00226	-1.8509751
AASS	214829_at	0.00000915	1.9751884	COX2	1553569_at	0.00226	-1.1036788
LUC7L3	241792_x_at	0.00000924	1.5448562	OSBP2	223432_at	0.00226	-1.2087743
EGR3	206115_at	0.00000924	1.911103	PAMR1	213661_at	0.00226	2.1404279
MIA3	1569057_s_at	0.00000924	-1.3221917	SEH1L	223225_s_at	0.00227	-1.1430804
SAV1	222573_s_at	0.00000926	-1.0769703	KIF26A	232069_at	0.00228	1.6677033
HLF	204755_x_at	0.0000094	2.6642741	SAA2-SAA4	207096_at	0.00228	-1.7793217
PAPPA	228128_x_at	0.00000945	3.1290961	ABCC3	230682_x_at	0.00228	1.9242627
GMDS	204875_s_at	0.00000949	-2.2310061	ZNF710	239700_at	0.00228	-1.3257072
RPP30	1556063_s_at	0.00000949	1.5562874	LRRIQ1	223817_at	0.00228	-1.580525
ABCC5	226363_at	0.00000949	1.2333272	DAB1	228329_at	0.00229	1.6309882
MITF	226066_at	0.00000957	1.468844	LOH12CR2	236311_at	0.0023	1.3710941
HM13	224615_x_at	0.00000957	-1.1479608	TRIM14	203147_s_at	0.0023	-1.2162904
C21orf59	218123_at	0.00000957	-1.1823354	PCDHB7	231738_at	0.0023	1.3908742
NUDT6	230329_s_at	0.00000961	1.0495643	SNORD73A	200099_s_at	0.00231	1.1456793
TSPAN31	203227_s_at	0.00000973	1.5496003	ENPEP	204844_at	0.00231	1.6027551
MUT	202959_at	0.00000974	1.152867	PTPN22	206060_s_at	0.00232	-1.7513103
YIPF5	224953_at	0.00000974	1.4274054	FUT8	1554930_a_at	0.00232	-1.2417839

GLIPR1	226136_at	0.00000976	1.4301994	CHST15	244874_at	0.00232	-1.5896956
WASIR2	1568838_at	0.0000099	2.5863768	SLC26A4	206529_x_at	0.00232	1.6681747
CYB561D2	229636_at	0.0000099	2.2721989	TNFAIP6	206026_s_at	0.00232	-1.372636
MICA	205904_at	0.00000992	1.8654063	ITGA1	214660_at	0.00232	-1.4501694
LETMD1	207170_s_at	0.00000993	1.0133255	PDE5A	227088_at	0.00232	1.6668272
HILPDA	1554452_a_at	0.00000995	-2.0679703	KCNMA1	221584_s_at	0.00232	1.828664
MAN2B1	209166_s_at	0.00000995	-1.0585006	RFX7	218430_s_at	0.00233	-1.0094091
ASPA	206030_at	0.00001	2.7182641	ZNF711	228988_at	0.00233	1.8218376
C21orf2	203994_s_at	0.0000101	2.3519814	IWS1	1562110_at	0.00233	-2.6733307
NDN	209550_at	0.0000102	2.0114087	GANC	235340_at	0.00233	-1.1000477
DEPDC1	220295_x_at	0.0000103	-1.6807101	TRMT1L	220992_s_at	0.00234	1.0428831
BRCC3	216521_s_at	0.0000103	-2.0422063	CELF1	1555467_a_at	0.00234	-1.561232
ALDH18A1	217791_s_at	0.0000103	-1.36089	ZNF439	236562_at	0.00235	1.3566506
TDO2	205943_at	0.0000103	-2.0305697	SFTPD	214199_at	0.00235	1.5406082
CARMN	231987_at	0.0000104	1.3685831	LACTB	1552485_at	0.00236	-1.4648172
STAT1	AFFX-HUMISGF3A/M97935_MB_at	0.0000104	-1.9003361	CDC45	204126_s_at	0.00236	-1.9671108
MPZL1	210087_s_at	0.0000105	-1.8727296	RUNX1T1	205528_s_at	0.00236	1.4368248
EPM2AIP1	236314_at	0.0000105	2.6609557	LOC102724250	230712_at	0.00237	1.1095733
CENPW	226936_at	0.0000105	-1.5312285	ABTB1	229164_s_at	0.00237	1.2394204
NAA15	219158_s_at	0.0000105	-1.266973	SAMHD1	1559883_s_at	0.00237	-2.1235585
SAA2	214456_x_at	0.0000106	-3.74062	CCNG2	211559_s_at	0.00237	-1.2997333
DIAPH3	229097_at	0.0000108	-1.612675	NDUFV2-AS1	242676_at	0.00237	1.1119093
CEP70	238154_at	0.0000108	1.2941651	SPP1	1568574_x_at	0.00238	-2.1907977
TMEM106B	226529_at	0.0000109	1.4089895	ACBD3	202323_s_at	0.00238	-1.1524923

RBM34	214942_at	0.0000109	-1.8642108	ZNF790-AS1	235779_at	0.00239	2.0003384
KDM2A	208987_s_at	0.0000109	-1.215965	GPR22	244493_at	0.00239	2.1824428
BTG2	201236_s_at	0.0000109	1.4558158	KHDC1	230055_at	0.00239	1.7228576
LOC101928269	211180_x_at	0.0000109	-1.7349949	DNAJC5	224613_s_at	0.0024	-1.1920033
LOC101928635	207016_s_at	0.000011	2.9865279	KIAA1755	1561394_s_at	0.0024	2.0434242
MAGI2	209737_at	0.000011	1.5257567	CKMT1A	202712_s_at	0.00241	-1.4038503
NAV1	224774_s_at	0.0000111	-1.7031082	TMEM56	237515_at	0.00242	1.1916045
ZC3H6	227809_at	0.0000111	1.2005286	INPP5D	203332_s_at	0.00242	-1.3467097
PLAA	209533_s_at	0.0000112	-1.0073873	ACCS	228264_at	0.00245	1.2631197
PLOD2	202619_s_at	0.0000112	-1.6318408	PWAR6	226591_at	0.00246	1.5157284
PPM1G	200913_at	0.0000113	-1.1022624	KRT7	1558394_s_at	0.00246	1.7680279
TAGLN2	200916_at	0.0000113	-1.5843532	ACACB	43427_at	0.00246	1.2234284
GPI	208308_s_at	0.0000113	-1.709479	TPM4	1567107_s_at	0.00247	-1.8745187
PPP2R5C	213305_s_at	0.0000113	-1.0064816	S100A2	204268_at	0.00247	-2.1133161
NCAPH	212949_at	0.0000115	-2.2646689	TMEM200A	234994_at	0.00247	1.7898887
RBM7	235045_at	0.0000116	1.0500752	DPY19L2	230158_at	0.00248	2.4873394
CLCC1	1555543_a_at	0.0000116	-2.0782205	PDK4	225207_at	0.00248	1.3074948
PTCH1	209815_at	0.0000116	1.7354258	ZC3H12C	231899_at	0.00249	1.6191048
ANXA2	213503_x_at	0.0000116	-1.3834516	WDFY3-AS2	1562953_s_at	0.00249	1.1847971
MTFR2	228069_at	0.0000117	-1.6889697	BORCS5	227723_at	0.0025	-1.1785591
TXNL1	235561_at	0.0000117	1.7341055	ARL5B	242727_at	0.0025	-1.0258423
NFATC4	236270_at	0.0000117	1.642686	ERI2	240604_at	0.0025	-1.4460708
GULP1	204237_at	0.0000117	1.9429566	CTHRC1	225681_at	0.0025	-1.779066
SLC36A1	213119_at	0.0000118	-1.2092844	KRT25	237905_at	0.00251	-1.1627295
SLC6A2	216610_at	0.0000118	2.3085556	ZNF571-AS1	229328_at	0.00253	2.1943478
BHLHE22	228636_at	0.0000119	-2.6624087	XIST	235446_at	0.00253	1.0864838
RCBTB2	204759_at	0.0000119	1.6199856	BPNT1	1554575_a_at	0.00254	-1.2480713

IGF2R	201393_s_at	0.0000119	-1.5015424	MCCC2	209623_at	0.00255	-1.1081143
EXO1	204603_at	0.0000119	-2.1358165	GDPD3	219722_s_at	0.00255	1.0517605
PKIB	231120_x_at	0.0000119	2.7699082	ALDH1A3	1558649_at	0.00255	1.6824529
NAP1L3	204749_at	0.000012	2.5900712	ERRFI1	224657_at	0.00255	1.0749865
STC2	203439_s_at	0.0000121	-1.886839	RDH5	236291_at	0.00256	1.2471472
ADIRF	203571_s_at	0.0000122	2.3793497	ADAMTSL4	226071_at	0.00257	1.2962326
ZNF93	1569241_a_at	0.0000122	-2.2116556	ANP32A-IT1	220710_at	0.00258	-1.6394304
TPTE2P5	240409_at	0.0000123	2.0927125	IL18	206295_at	0.00258	-1.3377983
ANXA2	201590_x_at	0.0000123	-1.463376	RNF207	242882_at	0.00259	1.5496835
TAF9B	226037_s_at	0.0000123	1.4062711	LOC101928910	1570396_at	0.00259	1.7236031
GART	212379_at	0.0000125	-1.1151944	CXCR2	207008_at	0.00259	-1.7088767
TPTE2P5	236770_at	0.0000125	2.5104571	DDX3X	201211_s_at	0.0026	-1.4832649
ATP8A2	231395_at	0.0000126	2.2849416	DNM3OS	235891_at	0.0026	1.8827344
GRK3	228771_at	0.0000127	1.8111415	MIR100HG	225381_at	0.00261	2.1404723
CDK1	210559_s_at	0.0000128	-1.912812	LOC100505570	242841_at	0.00261	-1.1983992
SNX21	1553961_s_at	0.0000128	1.1102272	EPPIN-WFDC6	206319_s_at	0.00261	1.852821
ID4	209291_at	0.0000129	2.0828074	ANKRD44	226641_at	0.00261	1.0425274
SOX4	201418_s_at	0.0000129	-1.1106455	RNASEH1	241343_at	0.00261	-1.4369349
ZDHHC21	229923_at	0.0000129	1.3270653	RNF145	226077_at	0.00262	-1.1051011
FGD6	219901_at	0.000013	-1.2128	CMYA5	233520_s_at	0.00262	1.170302
RECK	205407_at	0.000013	1.9883307	HIST1H3G	208496_x_at	0.00262	-1.3531034
LOC100996412	230292_at	0.000013	1.7630576	LOC285957	243601_at	0.00263	-1.7279575
ARPC3	208736_at	0.0000132	-1.203294	RPL11	200010_at	0.00264	1.3205733
UBAP1L	232663_s_at	0.0000134	1.4597385	LINC00271	1561106_at	0.00264	1.4251383
FHL1	201540_at	0.0000135	2.2274072	SERP2	239890_s_at	0.00265	1.5504117
EFS	204400_at	0.0000136	2.3303554	FBXO15	231472_at	0.00265	1.6731251
FAM188A	218297_at	0.0000137	1.2564402	LYPD1	212909_at	0.00265	-2.4698682

ASMTL	36553_at	0.0000138	1.0077027	RIF1	241820_at	0.00265	-1.0365827
NCAPG2	219588_s_at	0.0000139	-1.4743182	DCAF12L1	238205_at	0.00266	2.3489133
MOB1A	201298_s_at	0.0000139	-1.3248182	CCDC160	1563022_at	0.00267	-2.2309107
KCNIP4	236783_at	0.0000142	2.4972935	PSMC3	201267_s_at	0.00267	-1.4191082
STAM2	228254_at	0.0000143	1.1592721	SAMHD1	204502_at	0.00268	-1.0568381
HLF	204754_at	0.0000143	1.7620921	LOC101927609	239775_at	0.00268	1.1081472
TRIM45	242056_at	0.0000144	1.2351356	PRICKLE2	225968_at	0.00268	1.1649229
C1orf21	237098_at	0.0000145	1.741171	PPP5C	201979_s_at	0.00268	-1.3497221
MARVELD3	239350_at	0.0000146	-1.8356112	DENND1B	228032_s_at	0.00268	1.0189157
UBE2QL1	226612_at	0.0000146	2.0401581	SPTAN1	214925_s_at	0.00268	1.1917774
TRAM1	201399_s_at	0.0000147	-1.4024187	TAPT1	238798_at	0.00268	1.2661974
PER3	221045_s_at	0.0000147	1.8110883	GNAO1	204763_s_at	0.00269	1.7838464
COL6A6	230867_at	0.0000148	2.917513	METTL9	230395_at	0.00269	-1.2186258
DNAJC11	1555360_a_at	0.0000148	-1.3920726	SNX14	230759_at	0.0027	1.1687871
TTYH2	223741_s_at	0.0000149	1.8885674	GUCA1B	235528_at	0.0027	1.0110134
TRIM23	204732_s_at	0.0000151	1.0868008	DET1	1558348_at	0.00271	1.0478148
AURKA	208079_s_at	0.0000151	-1.797184	EPOR	209963_s_at	0.00271	1.3085516
SBK1	226548_at	0.0000152	-1.7429753	STIP1	213330_s_at	0.00271	-1.0972241
MYLIP	223130_s_at	0.0000152	1.226199	ASXL3	233536_at	0.00271	1.9603312
ZBTB38	225512_at	0.0000152	1.1428633	OSR1	228399_at	0.00271	1.7992812
NR2C1	229956_at	0.0000154	1.2684142	ZNF790-AS1	1558809_s_at	0.00271	1.6047267
RANGAP1	212125_at	0.0000154	-2.0380701	SEC14L1P1	217667_at	0.00271	2.2491558
ANKRD22	238439_at	0.0000154	-2.4206941	GYG2	215695_s_at	0.00271	-1.3606301
CACNA1G	207869_s_at	0.0000155	2.8505649	NUDT12	223535_at	0.00272	1.0959231
MINOS1-NBL1	37005_at	0.0000155	1.5920702	KLK11	205470_s_at	0.00272	1.9485066
BIRC5	210334_x_at	0.0000155	-1.7915321	MYRIP	214156_at	0.00272	1.9328106
PEG3	209243_s_at	0.0000157	3.1454713	ANKRD12	216550_x_at	0.00272	1.2024788

GARS	208693_s_at	0.0000157	-1.2041841	CYP39A1	244407_at	0.00273	1.266631
SEC63	201914_s_at	0.0000157	-1.1140873	ALYREF	226319_s_at	0.00273	-1.385586
SPIRE1	1554807_a_at	0.0000159	-2.839658	STK32B	219686_at	0.00273	2.2859361
MGST2	204168_at	0.0000159	-1.0863181	ACTR2	1558015_s_at	0.00273	-1.0521849
PSMA3-AS1	225724_at	0.0000159	1.0705824	SLAIN2	233874_at	0.00273	1.2881228
HEATR3	219289_at	0.000016	-1.0555097	D2HGDH	228738_at	0.00274	1.1554913
INTS6-AS1	1557118_a_at	0.000016	1.5945735	ZNF614	210599_at	0.00274	1.2460908
CBWD7	229804_x_at	0.0000161	1.0104654	FGF13	205110_s_at	0.00274	1.9271979
NAP1L1	1556121_at	0.0000162	1.0427271	FNDC3A	215910_s_at	0.00274	-1.5280985
PES1	236434_at	0.0000162	1.6968701	LAMA2	216840_s_at	0.00274	2.2453043
LEF1-AS1	243362_s_at	0.0000162	2.9019238	PFDN5	210908_s_at	0.00275	1.0793701
SMAD4	202526_at	0.0000167	1.2119119	SLC16A6	230748_at	0.00275	1.0601179
C4orf3	224604_at	0.0000167	1.6522129	SAFB	201747_s_at	0.00277	-1.7017793
EGR1	227404_s_at	0.0000167	1.7214348	CXCL13	205242_at	0.00277	-2.7897132
SMOC2	223235_s_at	0.0000167	2.5612647	CEP83	239282_at	0.00277	1.2673199
STXBP5	235227_at	0.0000167	1.2887595	HTR7P1	236115_at	0.00278	1.0553015
SLC38A10	230448_at	0.0000168	-1.4576847	SLC30A7	239596_at	0.00278	-1.0003644
SBNO1	218737_at	0.0000168	-1.0735729	GK	215977_x_at	0.0028	-1.3339252
PTGR2	230774_at	0.0000168	1.0247371	HECTD1	241955_at	0.0028	-1.051092
LINC01355	228387_at	0.0000169	1.6663758	RIOK3	202129_s_at	0.0028	-1.4932193
ANXA2	210427_x_at	0.0000169	-1.4648724	POU6F1	229809_at	0.0028	1.3836153
ZAK	225665_at	0.0000169	1.5556229	RDH13	1559190_s_at	0.00281	1.0537176
MTMR10	225810_at	0.0000169	1.0722353	STC2	203438_at	0.00282	-1.461696
BCHE	205433_at	0.000017	3.3908889	TXLNG	219969_at	0.00282	-1.2346
RASGRF2	228109_at	0.000017	1.5249851	SLC25A2	224166_at	0.00282	-1.0039061
AKIRIN1	217893_s_at	0.0000173	-1.5626077	MAGT1	210596_at	0.00283	1.3809787
NAT6	210874_s_at	0.0000173	1.8235278	SGO2	230165_at	0.00284	-1.0319328

SIX4	229796_at	0.0000174	-1.2013069	SNHG18	227655_at	0.00285	1.7231151
COL14A1	228750_at	0.0000175	1.8904345	TTC14	241863_x_at	0.00287	1.3271763
CARMN	227183_at	0.0000177	2.1841813	UBE2S	202779_s_at	0.00287	-1.5648272
TNKS2	222563_s_at	0.0000177	-1.6174056	LSM4	202736_s_at	0.00287	-1.3170669
CAMSAP1	212712_at	0.0000179	-1.1691249	LINC00261	228004_at	0.00287	2.9231292
CLUHP3	219442_at	0.0000179	1.3233186	PBRM1	224152_s_at	0.00289	-1.355116
LOC101927263	236396_at	0.000018	2.3471256	ST7-AS1	1555912_at	0.0029	1.1165025
PTPRN2	203029_s_at	0.000018	2.5744299	LOC283887	1556709_a_at	0.0029	1.3571368
ARPC5	1555797_a_at	0.000018	-1.2413364	C1GALT1	219439_at	0.00291	-1.1418131
PABPC4L	238865_at	0.000018	2.7305164	CD44	217523_at	0.00291	-1.8323111
ITGA2B	206493_at	0.0000182	2.0152703	ACAP1	242081_at	0.00293	1.2618473
MBLAC2	230298_at	0.0000183	1.0405931	PAK1	209615_s_at	0.00293	-1.0457917
FMOD	202709_at	0.0000183	2.0561578	ZNF446	219900_s_at	0.00293	1.0460251
ACSL3	201662_s_at	0.0000185	-1.2720562	LOC100287525	230606_at	0.00294	1.1751867
FLJ35934	1564383_s_at	0.0000185	2.4557002	CD93	202878_s_at	0.00294	-1.0676121
WDR17	1557132_at	0.0000185	2.8490348	MESP2	1556014_at	0.00294	-1.5743496
ARNTL2	220658_s_at	0.0000186	-2.758321	CD86	210895_s_at	0.00294	-1.5712692
ZBTB14	208199_s_at	0.0000187	-1.2272496	GSTA1	203924_at	0.00294	-2.0281285
RASD1	223467_at	0.0000188	2.5299023	FAHD2A	235621_at	0.00294	1.1419826
MYBL2	201710_at	0.0000189	-2.4119507	ST6GAL2	228821_at	0.00295	2.2544306
AQR	212583_at	0.0000189	1.0166972	FANCI	213007_at	0.00295	-1.033183
TMEM19	230676_s_at	0.0000191	-1.2203657	SCD	211162_x_at	0.00298	-1.4609451
MIR6787	202856_s_at	0.0000195	-1.7203838	DLC1	242631_x_at	0.00298	1.5661299
SH3GLB1	210101_x_at	0.0000198	-1.3131151	SOX2	228038_at	0.00298	-2.4687912
LOC102724851	230187_s_at	0.0000199	1.8972468	NFYA	204107_at	0.00299	-1.353905
CKS2	204170_s_at	0.0000199	-1.610388	SATB1	241365_at	0.003	2.4037402
LUC7L3	220044_x_at	0.00002	1.0327802	LOC100130285	1562799_at	0.003	1.4835179

IRGQ	1555833_a_at	0.00002	-1.154617	EPHB6	204718_at	0.003	1.1326084
MSX2	210319_x_at	0.00002	3.465834	HSP90B1	216449_x_at	0.00301	-1.7304703
FZD10-AS1	239569_at	0.0000201	2.4952241	HYMAI	215513_at	0.00301	1.4373964
GAPDH	AFFX-HUMGAPDH/M33197_M_at	0.0000201	-2.0139288	CYP1B1	202436_s_at	0.00302	1.4743416
KRTDAP	230835_at	0.0000201	2.3959017	BSG	208677_s_at	0.00306	-1.0183154
RPAP3	1557984_s_at	0.0000204	-2.2151163	CYP2C8	208147_s_at	0.00307	1.9068341
GNB5	204000_at	0.0000204	1.4757697	LOC105376805	229874_x_at	0.00308	1.0038204
PSENEN	218302_at	0.0000207	-1.5617022	FAM126B	231874_at	0.00309	-1.1735782
COPA	214336_s_at	0.0000207	-1.0789092	SNHG22	240006_at	0.00309	1.055489
MAGEH1	218573_at	0.0000208	1.8804597	DTNBP1	223446_s_at	0.00309	-1.9462095
UBR5-AS1	1555888_at	0.0000209	1.0529041	BROX	239078_at	0.00309	-1.5823814
AURKA	204092_s_at	0.000021	-1.8177467	TRDMT1	243504_at	0.00309	1.1461207
ANKRD46	212731_at	0.0000212	1.0576542	STEAP2	225871_at	0.00312	1.3774294
DHFR	202533_s_at	0.0000212	-2.0699777	ASAH2B	231791_at	0.00314	-1.6102555
SLF2	235590_at	0.0000212	1.16832	TMEM91	229957_at	0.00314	1.1956014
COL27A1	225293_at	0.0000212	2.4394389	MAPK1	208351_s_at	0.00314	-1.5981608
DTWD1	229984_at	0.0000213	1.130246	GALE	1557651_x_at	0.00318	-1.0251583
DHFR	202534_x_at	0.0000214	-1.0525313	CACYBP	210691_s_at	0.00318	-1.1772332
ATP1A1-AS1	236623_at	0.0000215	1.766053	MAPK1	1552264_a_at	0.00318	-1.0952476
UBASH3B	238587_at	0.0000217	-2.0180691	NMNAT3	228090_at	0.00318	1.1026915
DTWD1	236649_at	0.0000217	1.4628889	IGK	215176_x_at	0.00318	-3.10082
LOC101928487	231312_at	0.0000218	1.0482418	P4HB	200654_at	0.00319	-1.2684878
KAT2B	203845_at	0.0000219	1.3633314	KMT2C	232940_s_at	0.0032	-1.0545099
LOC101930476	227893_at	0.0000219	1.1434353	LOC100506472	235827_at	0.0032	1.280047
NSMCE4A	228506_at	0.000022	1.6826637	FAM223A	1552607_at	0.0032	1.0908357

MAGED2	213627_at	0.0000221	1.0703883	GAL3ST1	205670_at	0.0032	1.8003538
CHEK1	205394_at	0.0000221	-1.9339347	TUBA1B	201090_x_at	0.00321	-1.0395078
ENC1	201340_s_at	0.0000223	-1.9356663	GZMB	210164_at	0.00321	-1.6960232
PNISR	212176_at	0.0000223	1.0139455	SYNGR1	213854_at	0.00322	1.4262979
NQO1	201468_s_at	0.0000224	-2.5891837	SF1	210172_at	0.00322	1.2520255
MEX3A	236885_at	0.0000225	-1.84104	SCN5A	207413_s_at	0.00322	1.375854
ESPL1	204817_at	0.0000226	-1.6885839	SLC2A5	204430_s_at	0.00323	-1.0399951
ESPL1	38158_at	0.0000226	-1.839604	PPP1R3E	227409_at	0.00323	1.3279559
TTC39A	210652_s_at	0.0000227	-1.5972347	SCGB3A1	230378_at	0.00324	3.2563386
LIFR	225575_at	0.0000227	1.5155878	FOXP2	235201_at	0.00325	2.4588006
PAGE4	205564_at	0.0000227	2.4468305	RAB21	241263_at	0.00325	-1.4142558
IGDCC3	230960_at	0.0000227	1.8267369	FBXL7	213249_at	0.00325	1.3836318
ELOF1	225156_at	0.0000227	-1.3903086	ADPRHL1	238054_at	0.00325	1.4330157
FAM208A	209285_s_at	0.0000228	1.0633917	ITGA10	206766_at	0.00325	1.0598018
LINC00936	238893_at	0.0000229	1.1799697	GPER1	210640_s_at	0.00325	1.7789887
CENPN	228559_at	0.0000229	-1.220544	CEP128	232635_at	0.00325	-1.2468865
PRC1	218009_s_at	0.0000229	-1.7136991	DCT	205338_s_at	0.00325	1.6934952
FLT1	222033_s_at	0.000023	-1.1818429	TSEN54	241402_at	0.00326	1.454659
NDNF	219747_at	0.000023	2.223707	CD47	211075_s_at	0.00326	-1.5344157
ELF5	220625_s_at	0.000023	-3.8339234	ZEB2	235593_at	0.00326	1.5574957
THRA	35846_at	0.0000231	1.1028309	MCM10	220651_s_at	0.00327	-2.1615938
C1orf168	238625_at	0.0000231	2.851266	ENO1	217294_s_at	0.00327	-2.0803061
SLC6A8	210854_x_at	0.0000232	-1.7945022	THNSL1	222931_s_at	0.00328	1.1831576
LOC100506718	240259_at	0.0000233	2.7216478	PECAM1	208982_at	0.00329	-1.1402965
PAK3	214078_at	0.0000233	3.1123662	TGM3	206004_at	0.00329	-1.4646279
PDCD5	227751_at	0.0000235	1.1278461	RNF187	230662_at	0.00329	-1.3953182
LOC100506691	241386_at	0.0000235	2.0548032	SNORD68	229590_at	0.0033	1.2019642

GMFB	202543_s_at	0.0000236	-1.1560945	EMB	226789_at	0.0033	-1.3626591
FGD6	1555137_a_at	0.0000236	-2.4226753	CKAP2L	229610_at	0.0033	-1.4217898
USP53	230083_at	0.0000236	1.3104588	TMOD2	226186_at	0.00332	1.452755
ARF3	200011_s_at	0.0000236	-1.2695269	BGN	213905_x_at	0.00332	-1.321922
BAIAP2L1	222675_s_at	0.0000238	-1.5877529	EZR	208622_s_at	0.00333	-1.2011118
RPL15	240806_at	0.0000238	1.6426138	C6orf132	238028_at	0.00335	-1.1134311
PDIA4	208658_at	0.0000239	-1.064225	REM2	235699_at	0.00335	1.0979754
FOXO3B	204132_s_at	0.0000239	-1.1600167	SAMSN1	220330_s_at	0.00335	-1.284089
FAXDC2	220751_s_at	0.0000239	2.5772268	GSTM1	204550_x_at	0.00336	1.1798636
CDC16	209658_at	0.0000239	1.0180401	TMEM65	238045_at	0.00336	-1.0189905
SLC1A1	213664_at	0.000024	1.8764869	LRP6	205606_at	0.00336	-1.2996701
UBE3C	1554794_a_at	0.000024	-1.7417775	SFPQ	201586_s_at	0.00336	-1.0803637
SH2B1	40149_at	0.000024	1.1926479	DLEU2	242854_x_at	0.00336	-1.6515238
NUDT11	219855_at	0.0000241	2.1546717	NRG2	242303_at	0.00336	1.4190599
RPS6KA6	228503_at	0.0000243	2.6635786	F12	205774_at	0.00337	-1.6358883
ENOSF1	213645_at	0.0000244	1.7629959	ALYREF	226320_at	0.00339	-1.6349726
HIPK2	225368_at	0.0000244	-1.3687863	GABPB1-AS1	235274_at	0.0034	1.4960345
C1QTNF7	239349_at	0.0000244	2.2595676	CRK	202226_s_at	0.0034	-1.3664525
OTUD1	226140_s_at	0.0000244	1.6578938	NEK10	237227_at	0.0034	-1.7213762
CD24	208650_s_at	0.0000245	-1.6259431	STK3	211078_s_at	0.00341	-1.952475
TMEM167A	226276_at	0.0000246	-1.375806	GPATCH2	239768_x_at	0.00341	-1.1531022
TLE2	40837_at	0.0000246	1.7372602	CRIPT	239049_at	0.00341	1.2300888
COMMD6	225312_at	0.0000247	1.3502139	SLC16A1	202234_s_at	0.00341	1.6899159
NMT2	205005_s_at	0.0000248	1.3686153	SYNPO	202796_at	0.00342	-1.2397218
DST	204455_at	0.0000251	-2.0674566	WAS	38964_r_at	0.00343	-1.015943
ATAD2	222740_at	0.0000252	-1.8366783	FBXO32	225328_at	0.00343	1.4486627
RARA-AS1	228037_at	0.0000254	1.1438341	SAMD4A	212845_at	0.00343	1.0534425

F10	205620_at	0.0000255	1.9402632	UBE2Q2L	244498_x_at	0.00343	1.0069903
MYO15B	219173_at	0.0000255	2.6695121	CHGB	204260_at	0.00344	1.5823443
METTL3	213653_at	0.0000255	1.1068613	GABRB3	227830_at	0.00344	1.3911817
PTBP3	214698_at	0.0000256	-1.1155577	NGFR	205858_at	0.00345	-1.5878869
TUBB	211714_x_at	0.0000256	-1.5679477	PDAP1	202290_at	0.00346	-1.072815
RGS7BP	237719_x_at	0.0000257	3.111372	UBE4B	215533_s_at	0.00347	-1.1772976
ZNF37BP	215358_x_at	0.0000257	1.4896196	PPID	204186_s_at	0.00348	-1.1627775
AZGP1	209309_at	0.0000258	2.4375053	PDE6B	1557777_at	0.00349	1.4698674
HNRNPA3	211931_s_at	0.0000258	-1.0234421	CHST6	223786_at	0.0035	-2.5930866
PGM5-AS1	230595_at	0.0000259	2.752814	CCNJL	219227_at	0.0035	-1.1069045
THAP2	230380_at	0.0000259	1.0142046	LRRN1	226884_at	0.0035	2.9080023
SYNDIG1	219310_at	0.000026	2.0321434	SCOC-AS1	1556736_at	0.0035	1.5455968
SMIM10L2B	227909_at	0.000026	1.958602	LOC101928269	210365_at	0.00351	-1.2765071
CPD	201943_s_at	0.0000261	-1.4507275	SCARA3	223842_s_at	0.00352	1.3480782
SCML1	218793_s_at	0.0000262	2.2388702	LOC105372404	1562825_at	0.00353	2.0410036
ZNF148	239024_at	0.0000263	1.1064624	LOC100507334	229096_at	0.00353	2.277324
SEPP1	201427_s_at	0.0000264	2.0526299	SNRPB	213175_s_at	0.00354	-1.1559949
MSANTD2	221208_s_at	0.0000266	1.4996571	CHDH	231994_at	0.00356	1.204994
ZDHHC20	243786_at	0.0000267	-1.0820438	RFWD3	218564_at	0.00356	-1.0939378
GAPDH	217398_x_at	0.0000269	-1.8185698	LOC100130476	243871_at	0.00356	1.6240441
PLAGL1	209318_x_at	0.000027	1.9345808	CD24	208651_x_at	0.00356	-1.0022562
GPR137B	204137_at	0.000027	1.5437166	NABP1	222872_x_at	0.00356	1.2244288
CRIPT	239050_s_at	0.000027	1.1140019	ZNF493	211064_at	0.00358	1.121301
LOC145783	221213_s_at	0.0000272	1.2356751	IBA57-AS1	238188_at	0.00358	1.2854632
RBM15	1555760_a_at	0.0000273	-1.0396924	VNN3	220528_at	0.00358	-2.317492
COCH	205229_s_at	0.0000277	-2.7521809	LEF1	221558_s_at	0.00358	1.6123048
KIAA1107	1558293_at	0.0000277	1.3111851	ZMYND11	1554159_a_at	0.00358	-1.6021065

PEX13	1556009_at	0.0000277	-1.3728181	GNRH1	207987_s_at	0.00359	1.2844694
TAGLN2	210978_s_at	0.0000278	-1.5721441	HNRNPUL2-BSCL2	66053_at	0.00361	-1.2630743
RFX5	202964_s_at	0.0000278	-1.1861587	DYNC1I1	205348_s_at	0.00361	2.1552293
MCM10	223570_at	0.0000278	-1.8897244	GALNT18	233188_at	0.00361	-1.2280855
HLCS	207833_s_at	0.0000278	-2.411612	HHIP-AS1	236632_at	0.00361	1.2239137
PUS10	229362_at	0.0000278	1.0532063	FBXO21	212229_s_at	0.00362	1.0173776
KIF21A	231875_at	0.0000279	-1.9779643	PAM	202336_s_at	0.00362	1.0401196
USP13	227788_at	0.000028	-1.498076	PAPSS2	203060_s_at	0.00364	-1.6310159
GPSM2	233058_at	0.000028	-1.6188689	PKM	201251_at	0.00367	-1.2073618
IRS4	1560652_at	0.0000281	2.215776	SNORD54	216247_at	0.00367	1.0799739
KLHL28	228328_at	0.0000281	1.1902484	DEFB124	1568375_at	0.00367	1.380129
PRPF40B	226966_at	0.0000282	1.2166808	PCGF3	230408_at	0.00368	1.1772362
TRO	211700_s_at	0.0000282	3.1454568	HNRNPDL	1554678_s_at	0.00368	-1.0243348
GLIDR	1555874_x_at	0.0000282	1.3249619	PDCD4-AS1	222307_at	0.00369	1.1612142
NIPAL2	227001_at	0.0000284	1.3688175	PITRM1	239378_at	0.00369	-1.1560162
HAPLN1	230895_at	0.0000286	-3.0706991	LAMP2	200821_at	0.00371	1.0751256
ERCC1	228131_at	0.0000286	1.335522	AFF4	1555436_a_at	0.00372	-1.0587891
BBS10	219487_at	0.0000286	1.4699497	TRNP1	227862_at	0.00373	-1.4084167
DACT2	1569648_at	0.0000286	2.1400755	NOTCH2NL	214722_at	0.00374	1.1103725
IMPACT	218637_at	0.0000287	1.1815712	API5	214959_s_at	0.00374	-1.0794691
CDKN3	209714_s_at	0.0000287	-1.6859705	LILRB2	207697_x_at	0.00375	-1.366586
GDAP1	226269_at	0.0000288	1.8076771	CHMP4B	225119_at	0.00375	-1.1410277
KLHL24	221985_at	0.0000288	-1.2794057	ZSWIM8-AS1	214867_at	0.00376	1.2341691
DEPDC1B	226980_at	0.0000288	-2.3436488	ASPRV1	235514_at	0.00376	1.0364556
PRPF40A	226687_at	0.0000289	1.1330086	UGGT1	222568_at	0.00377	-1.4841849
NDE1	227249_at	0.0000294	-1.3273259	LDLRAD4	207996_s_at	0.0038	1.1687849

EEF1A1	227708_at	0.0000294	1.5665997	SNX21	226595_at	0.00383	1.2574131
RANBP9	202583_s_at	0.0000296	-1.0058258	PDK4	1562321_at	0.00383	2.2143864
TTL	224896_s_at	0.0000299	-1.1300498	PRKCB	227824_at	0.00383	-1.667176
NDFIP1	222423_at	0.0000302	1.0689569	MYH3	205940_at	0.00383	1.442255
DCAF8	202250_s_at	0.0000303	1.0422931	HPGD	203913_s_at	0.00383	2.8817129
CENPI	214804_at	0.0000304	-1.2093285	METTL21A	235177_at	0.00383	-1.0029227
KLF4	221841_s_at	0.0000304	1.8354744	C10orf25	1552422_at	0.00383	1.2758197
DCN	211896_s_at	0.0000306	2.2900897	TBC1D8	241017_at	0.00384	1.0174022
CASC7	213310_at	0.0000307	-1.0315778	FCGR3B	204007_at	0.00388	-1.1052786
BEX2	224367_at	0.0000307	2.6221111	LOC101926934	241256_at	0.00391	1.4224136
MMP9	203936_s_at	0.0000307	-2.124327	TMEM50B	231696_x_at	0.00391	1.594819
LYN	210754_s_at	0.0000308	-1.1984533	DISC1FP1	1557845_at	0.00392	1.6029597
ECT2	234992_x_at	0.000031	-2.6319429	TMC5	240303_at	0.00396	-2.2640075
CMKLR1	210659_at	0.0000311	-1.3635671	COCH	1554242_a_at	0.00396	-2.0294995
ST14	202005_at	0.0000315	-1.4680999	HLA-F-AS1	222279_at	0.00396	1.2316989
PTGFR	207177_at	0.0000316	2.2805387	ARHGEF28	232994_s_at	0.00396	1.0538081
C16orf59	219556_at	0.0000316	-1.5498926	BRAP	209922_at	0.00398	-1.4906549
FANCI	213008_at	0.0000316	-1.5958709	ARSG	230856_at	0.00399	1.3129604
TIA1	201449_at	0.0000317	1.0807835	S100P	204351_at	0.00399	-2.7332056
TIPARP	212665_at	0.0000317	1.0979799	SAMD11	1560477_a_at	0.00399	1.3375538
ECT2	219787_s_at	0.0000319	-1.5288164	CCT2	201946_s_at	0.004	-1.1319657
PPP5D1	242253_at	0.000032	1.1397584	TXNIP	201009_s_at	0.00401	1.1003545
MIF	217871_s_at	0.000032	-1.2662759	STC1	204597_x_at	0.00401	-1.1621604
MICAL2	212472_at	0.0000321	-1.1897879	EDNRB	204273_at	0.00402	1.9650272
SKIL	206675_s_at	0.0000322	-1.9183583	PCDH17	227289_at	0.00403	-1.253868
RAD51L3-RFFL	1552651_a_at	0.0000323	-1.6007035	KIZ	233241_at	0.00404	1.7079879
NECTIN4	223540_at	0.0000324	-1.8049778	ZDHHC2	222731_at	0.00406	1.6712994

FBN1	235318_at	0.0000339	2.0283722	EPDR1	223253_at	0.00406	1.3532419
GTSE1	204318_s_at	0.0000339	-1.75684	C5	205500_at	0.00408	1.0076294
CCL20	205476_at	0.0000346	-4.1609235	PAX6	205646_s_at	0.00408	-1.9613487
F11R	221664_s_at	0.0000348	-1.1799746	MINOS1	228096_at	0.00408	1.1639277
CTSS	202902_s_at	0.000035	-1.787045	RBBP9	232751_at	0.00409	-1.5999939
RPS6KA6	228502_at	0.0000351	3.0186002	CLEC4M	210481_s_at	0.00409	1.3866219
ROR1	232060_at	0.0000351	2.0395547	PSMA3	201532_at	0.0041	-1.2248397
UBE2D2	201343_at	0.0000354	-1.1635771	ZNF806	236328_at	0.0041	1.8030828
PAPOLA	228569_at	0.0000355	1.1257122	SRPRA	200917_s_at	0.00412	-1.4027097
KLF16	226328_at	0.0000359	-1.9605889	IGHV4-31	217236_x_at	0.00418	-1.6127239
MRGPRF	227727_at	0.0000362	2.0736857	XRCC4	205071_x_at	0.00421	-1.8092751
ZNF214	243456_at	0.0000364	2.4026175	DOCK5	230263_s_at	0.00422	1.5504845
GDE1	202593_s_at	0.0000364	-2.4079547	KIZ	228291_s_at	0.00422	1.2579919
TTC14	225180_at	0.0000365	1.7321011	RARG	204188_s_at	0.00422	-1.2022136
CDK1	203213_at	0.0000365	-1.9981102	DNAJC12	223722_at	0.00423	1.5623395
BMPR1A	242731_x_at	0.0000366	1.2592383	ZNF880	235913_at	0.00424	1.3195129
MAD2L1	236312_at	0.0000366	-1.232749	LTBP1	202728_s_at	0.00425	-1.5350589
GNG7	206896_s_at	0.000037	2.0389134	FAM134B	218532_s_at	0.00425	1.2575273
ACTA2	243140_at	0.000037	2.9120782	PRELP	204223_at	0.00426	1.6672791
TRMU	204317_at	0.000037	-1.7601938	ZFP92	233116_at	0.00427	1.174601
PAN3-AS1	243092_at	0.0000374	1.609376	ABCC3	208161_s_at	0.00428	2.1591037
LARP4	238959_at	0.0000375	-1.7454205	ESCO2	235588_at	0.00428	-1.0745987
SRI	208920_at	0.0000381	1.0926191	ANGPT2	236034_at	0.00428	-1.2396557
F5	204714_s_at	0.0000384	-2.2460164	PRICKLE1	226065_at	0.00428	1.5124898
GRAMD1C	219313_at	0.0000384	1.662279	KNDC1	230359_at	0.00428	1.3678226
INVS	235031_at	0.0000384	1.1859327	ACSL5	222592_s_at	0.00428	2.2358244
CDCP1	218451_at	0.0000386	-1.0196302	ST3GAL1	225034_at	0.0043	-1.2282165

LMLN	1553284_s_at	0.0000392	-1.5584824	ANAPC4	232524_x_at	0.00432	1.3766203
ZNF230	1557322_at	0.0000393	1.0737039	GATM	203178_at	0.00432	1.7584496
INTS6-AS1	1557117_at	0.0000394	2.0591208	TJP3	213412_at	0.00434	-1.5616174
SHMT2	214095_at	0.0000394	-1.0480714	HSPD1	200806_s_at	0.00435	-1.3391602
MIR3658	209825_s_at	0.0000396	-1.4351492	APBB2	213419_at	0.00435	-1.0269951
MUM1L1	229160_at	0.0000401	2.6559628	UPP1	203234_at	0.00435	-1.0389033
MIR6516	221621_at	0.0000401	1.1174034	C3orf18	219114_at	0.00435	1.1226651
SLC16A10	222939_s_at	0.0000401	-3.2345319	NUMA1	235539_at	0.00437	1.1642337
SLC6A8	213843_x_at	0.0000404	-1.9563499	EIF4E3	225939_at	0.00438	1.3673013
TLCD1	227804_at	0.0000408	-1.100647	NOP14-AS1	214685_at	0.00438	1.6016889
PLAGL1	207943_x_at	0.0000411	1.7739844	CCDC57	227783_at	0.0044	1.5135877
LOXL2	202998_s_at	0.0000412	-1.3120642	RASEF	235144_at	0.00441	-1.2324706
CENPO	226118_at	0.0000413	-1.1176572	DYNC1H1	241084_x_at	0.00441	-1.498225
KIAA1549	206044_s_at	0.0000413	-1.0627715	TYMS	202589_at	0.00442	-1.1776866
SSBP2	238861_at	0.0000416	1.7632007	TFRC	207332_s_at	0.00442	-1.255642
COBLL1	203642_s_at	0.0000417	-1.9456799	SLC25A35	236064_at	0.00442	1.9598181
LOC285812	230179_at	0.000042	-1.7833106	TMEM161B	238783_at	0.00442	1.1637027
LOC100507468	239113_at	0.0000425	1.9101374	CENPN	219555_s_at	0.00444	-1.3008695
RUVBL1	201614_s_at	0.000043	-1.9837398	SIX1	205817_at	0.00445	-1.75097
REV1	222629_at	0.0000435	1.0390688	STXBP2	1570420_at	0.00446	-1.3021568
CDYL	203099_s_at	0.0000437	-1.7940063	LOC645513	239556_at	0.00449	1.0059753
TGFBR3	226625_at	0.0000439	1.9515018	PCP2	244751_at	0.00449	1.8326254
ATAD2	235266_at	0.0000442	-1.9774876	DCAKD	221224_s_at	0.0045	-1.2450535
GHITM	1554510_s_at	0.0000442	-1.0259107	CTSC	201487_at	0.00453	-1.2129219
DSP	200606_at	0.0000449	-1.4511864	ERO1B	231944_at	0.00455	1.8478177
CEP89	1554513_s_at	0.0000449	-2.2464501	HEATR3	1554478_a_at	0.00455	-1.1785374
WDR48	221735_at	0.0000451	1.0659788	TUBA1B	212639_x_at	0.00455	-1.0093434

TMEM243	204215_at	0.0000452	1.2114492	JMJD1C-AS1	1552665_at	0.00456	1.0200141
TMC5	222904_s_at	0.0000453	-2.1219359	ADGRF1	236489_at	0.00456	-2.0648116
HES6	226446_at	0.0000455	-2.445444	SYTL4	227703_s_at	0.00457	1.2523707
C10orf12	226892_at	0.0000455	-1.0375232	FOXA1	237086_at	0.00458	-2.9485773
DHCR24	200862_at	0.0000455	-1.7006338	DLX6-AS1	230882_at	0.00458	2.2484004
ME3	204663_at	0.0000455	1.6175163	SRSF3	235324_at	0.00458	1.0316245
ZEB1-AS1	229090_at	0.0000456	1.0106676	HM13	1558561_at	0.00458	-1.0634269
SOX7	228698_at	0.0000458	1.8011277	RBPMS	209488_s_at	0.00459	1.1864352
LZTFL1	218437_s_at	0.0000461	1.195212	MED27	215371_at	0.00459	-1.3625891
NAALAD2	1554507_at	0.0000461	3.3706088	LOC101930404	221974_at	0.0046	1.1667337
LYN	202626_s_at	0.0000462	-1.1026973	LPAR6	218589_at	0.00461	1.3895848
TRIP11	230211_at	0.0000463	1.8511538	RPL10L	217559_at	0.00462	1.3116496
ENAH	1553672_at	0.0000466	-1.341536	ITGA2B	206494_s_at	0.00463	1.5183634
ELP2	230178_s_at	0.0000469	1.1989121	CAMTA1	213268_at	0.00464	1.7059164
ZFP36L2	201369_s_at	0.000047	1.6353969	CNTN1	227202_at	0.00464	2.5273136
ZWINT	204026_s_at	0.0000471	-1.6345209	LUM	229554_at	0.00465	1.5473477
PODN	233148_at	0.0000472	1.5987199	LOC441242	236929_at	0.00465	1.4663041
SLC17A5	221041_s_at	0.0000474	-1.2517341	CHEK1	229423_at	0.00466	1.3612732
SLC25A36	223698_at	0.0000475	1.4313675	P2RX7	230741_at	0.00466	1.3384316
CEP290	205250_s_at	0.0000475	1.1468375	RAPGEF1	225738_at	0.00466	-1.114131
DAGLB	225832_s_at	0.0000477	-1.5042846	RHBDL2	1554897_s_at	0.00466	-1.5797255
HEXDC	1555866_a_at	0.0000479	1.0145364	LOC101928068	1557674_s_at	0.00467	1.4775812
ATL2	1553603_s_at	0.0000481	-1.1052148	HIPK1	1552516_a_at	0.00468	-1.6084772
RHOJ	235489_at	0.0000488	1.9618753	LINC00304	1564334_at	0.00471	1.5793427
TLE4	204872_at	0.0000488	2.033777	ZNF667	236635_at	0.00472	2.0263134
STRN3	215505_s_at	0.0000492	-1.8983328	PSMA7	201114_x_at	0.00473	-1.0613405
VPS53	1559108_at	0.0000493	-2.2181453	IRS2	209184_s_at	0.00474	1.2224206

PRDX1	208680_at	0.0000493	-1.4200924	FBXL17	238174_at	0.00474	1.0053336
ZDHHC12	226088_at	0.0000498	-1.8519593	ATP1A1	220948_s_at	0.00474	-1.0214121
PAM	212958_x_at	0.0000499	1.6077399	RXFP1	238206_at	0.00474	3.1805411
CACFD1	219223_at	0.0000499	-1.2808188	MBNL2	203640_at	0.00478	1.0083459
PPIB	200968_s_at	0.0000504	-1.4158541	BHMT2	242169_at	0.00479	1.6723754
DUSP1	201044_x_at	0.0000508	1.4597959	PIP5K1A	211205_x_at	0.00479	-1.3958639
ZNF273	215239_x_at	0.0000511	1.0488042	ETS1	224833_at	0.00479	-1.0803442
ARGLU1	218067_s_at	0.0000512	1.0876105	NEIL3	219502_at	0.0048	-1.3321721
ANGPT2	205572_at	0.0000513	-2.1661749	MARCKSL1	200644_at	0.0048	-1.1002304
A2M-AS1	1564139_at	0.0000513	1.1046165	ANKRD20A5P	1559170_at	0.0048	1.4793089
PPFIA1	210236_at	0.0000514	-1.1672837	STS	203768_s_at	0.00481	-1.1818682
KIAA0101	211713_x_at	0.0000516	-1.5698567	ESRRG	209966_x_at	0.00483	-1.2238774
HNMT	204112_s_at	0.0000516	1.7792718	PRCD	230015_at	0.00483	1.2451277
SLC25A16	214140_at	0.0000517	-1.1789165	NACAD	213630_at	0.00483	1.5116582
POLQ	219510_at	0.0000518	-1.7838692	RAP1A	1555340_x_at	0.00485	-1.3614912
DCN	242605_at	0.0000521	1.9369588	SDHC	210131_x_at	0.00486	-1.1084049
EFS	210880_s_at	0.0000522	2.0035376	SHROOM4	232404_at	0.00486	1.5614392
ETS1	1555355_a_at	0.0000528	-2.2498241	PFKFB4	228499_at	0.00487	-1.0077377
ZXDA	243521_at	0.0000531	1.0064421	EPPK1	208156_x_at	0.00487	-1.1028281
ESYT2	224699_s_at	0.0000533	-1.0922848	LOC101930114	237154_at	0.0049	1.4310813
ANXA3	209369_at	0.0000533	1.4063126	ITGB2	202803_s_at	0.0049	-1.0000458
OTUD6B-AS1	226341_at	0.0000535	1.1769274	RPS27L	238935_at	0.0049	1.1186354
WBP2NL	1552514_at	0.0000536	2.764068	SIRPB1	1554624_a_at	0.00491	-1.6325036
JUN	201464_x_at	0.0000536	1.0874239	KAZALD1	217511_at	0.00491	1.6862106
SURF1	217646_at	0.0000536	1.1772367	NLGN1	231361_at	0.00491	2.1036942
ULBP2	238542_at	0.0000536	-2.2848782	RBPMS	209487_at	0.00494	1.0506766
PAPLN	226435_at	0.0000541	2.0077047	HGF	210755_at	0.00494	1.1752776

GHR	205498_at	0.0000541	2.4555647	GJA4	204904_at	0.00495	1.7253122
ECHDC1	223088_x_at	0.0000542	-1.1471144	SPIRE1	1559517_a_at	0.00497	-1.5792453
TRMT1L	233750_s_at	0.0000547	-1.332679	CELF2	1554569_a_at	0.00499	-1.135912
KIF2A	203087_s_at	0.0000547	-1.1419764	IGF2BP3	203820_s_at	0.00499	-2.2053477
NAP1L2	219368_at	0.0000547	2.0234997	RANBP2	201711_x_at	0.00499	-1.0499405
CYB5B	238554_at	0.0000547	1.0148993	NR4A3	216979_at	0.005	1.2279491
USP51	229278_at	0.0000547	1.974773	PPIEL	222054_at	0.005	1.5272499
RSBN1	222790_s_at	0.0000548	1.0459289	ST8SIA4	242943_at	0.005	-1.6579217
CLDN7	202790_at	0.0000555	-1.7816806	PTGIS	208131_s_at	0.00501	1.829255
PDXK	202671_s_at	0.0000561	-1.1947737	SIM2	206558_at	0.00502	-1.3468187
LOC100507283	1563776_at	0.0000561	1.983012	RASSF8-AS1	242358_at	0.00504	1.4673466
TCP11L1	205796_at	0.0000563	-1.6586499	SNORA11E	221261_x_at	0.00505	1.8214053
NQO1	201467_s_at	0.0000567	-2.7789275	EIF1	228967_at	0.00505	1.0390872
PCDHB15	231789_at	0.0000568	2.3304255	PRDM6	236577_at	0.00506	1.5177718
LPCAT2	239598_s_at	0.000057	1.1041775	HMGA1	206074_s_at	0.00506	-1.2488649
RNASE4	213397_x_at	0.000057	2.0982928	MTFR2	234944_s_at	0.00507	-1.0820548
ARL8B	222442_s_at	0.0000573	-1.1346499	HIPK3	207764_s_at	0.00508	-1.5749964
FUT9	207696_at	0.0000574	2.3981872	MDFIC	211675_s_at	0.00508	1.3579059
DCHS1	222101_s_at	0.0000578	1.744811	THEM4	229253_at	0.0051	1.0356317
LOC101928560	244618_at	0.0000579	1.2138602	KALRN	205635_at	0.00511	-1.1312616
DLAT	212568_s_at	0.0000579	-1.0967335	PRDM1	228964_at	0.00511	1.1782496
RBM26	226316_at	0.000058	1.1656327	LPP	1558469_at	0.00511	-1.2704014
CHRDL1	209763_at	0.0000582	2.8446859	SGCE	204688_at	0.00512	1.3495007
CD200	209583_s_at	0.0000583	1.9431636	SNX13	215820_x_at	0.00513	-1.2230306
PIGL	239943_x_at	0.0000584	1.7820575	RAB28	234628_at	0.00514	-1.1404827
MAD1L1	204857_at	0.0000586	-1.922992	DPP10-AS1	236351_at	0.00514	2.3548972
LOC101930404	241834_at	0.0000593	1.9539799	CCDC24	228994_at	0.00515	1.4221144

APOE	203381_s_at	0.0000593	-2.203665	EMP2	225079_at	0.00516	-1.0236818
MED30	227786_at	0.0000601	1.236651	CENPJ	234023_s_at	0.00519	-1.6519015
PDK1	226452_at	0.0000605	-1.5223422	TNFRSF21	214581_x_at	0.0052	-1.4647588
LOC101929500	202552_s_at	0.0000605	1.331778	HELLS	220085_at	0.00522	-1.2452275
ICAM1	202638_s_at	0.0000605	-1.2358515	NUDT2	218609_s_at	0.00523	1.3510153
FAM98A	212333_at	0.0000606	-1.0514115	LOC101927137	214796_at	0.00526	1.2552236
GLI1	206646_at	0.0000609	1.9955343	SFRP1	202037_s_at	0.00526	1.7870561
AURKB	209464_at	0.0000612	-1.343611	SORBS2	225728_at	0.00527	1.4990434
GOLGA8N	213737_x_at	0.0000612	1.5732378	CACNB2	207776_s_at	0.00527	1.1623347
CARMN	1558828_s_at	0.0000613	3.0521484	NOL12	222057_at	0.00528	1.0183365
GAPDH	212581_x_at	0.0000615	-1.4107399	SLAMF8	219386_s_at	0.00528	-1.4615787
ABCF2	207622_s_at	0.0000621	-2.0147199	HAUS7	207891_s_at	0.00529	-1.0783388
RRAGD	221524_s_at	0.0000621	-2.2121742	DOCK5	230207_s_at	0.0053	1.4910207
LPAR1	204036_at	0.0000622	1.5206929	SP2-AS1	238953_at	0.0053	1.0596289
KIAA1328	232358_at	0.0000622	1.2224902	MGARP	223734_at	0.0053	1.1782379
TPST2	204079_at	0.0000622	1.7780996	CLEC2B	209732_at	0.0053	1.2103034
RFXAP	229431_at	0.0000624	1.047389	LOC101926921	240873_x_at	0.0053	-1.5704454
GKAP1	234192_s_at	0.0000627	1.0711915	LRFN4	219491_at	0.00531	-1.6674091
G3BP2	206383_s_at	0.0000628	-1.0102891	AKAP10	205045_at	0.00533	-1.4271224
ZEB2	228333_at	0.0000628	1.2918082	COX10-AS1	230451_at	0.00534	1.6399252
RASSF5	223322_at	0.000063	-1.0115523	STON2	235852_at	0.00534	-1.6017056
SGPL1	212322_at	0.000063	-1.0421492	VEGFC	209946_at	0.00534	1.0337252
SIDT2	218765_at	0.0000632	1.0109533	ELFN2	1559072_a_at	0.00535	-1.6643655
ACSL5	218322_s_at	0.0000636	2.6629904	SNTB1	214708_at	0.00535	-1.5876993
N6AMT1	234676_s_at	0.0000637	1.6531352	EXOC6B	215417_at	0.00535	1.0388812
LOC101930324	202395_at	0.0000637	-1.1765947	ST3GAL1	225033_at	0.00536	-1.1858246
ARF4	201096_s_at	0.000064	-1.3490705	NRK	227971_at	0.00538	1.5172263

CDC7	204510_at	0.0000641	-1.3486295	EMID1	1564251_at	0.00539	-1.4747568
WDFY3-AS2	238081_at	0.0000641	1.6776827	THAP2	223588_at	0.0054	1.4792921
PRCC	208938_at	0.0000641	-1.1197926	NENF	214125_s_at	0.0054	1.2369261
GAPDH	AFFX-HUMGAPDH/M33197_3_at	0.0000644	-1.1615546	LMF1	227984_at	0.00541	1.4418663
ORC6	219105_x_at	0.0000644	-1.2632666	ERFE	229622_at	0.00541	-1.7463723
PPP2R3A	207749_s_at	0.0000647	-1.2422205	RGN	210751_s_at	0.00542	1.3701791
WAPL	1554441_a_at	0.0000648	-1.0975329	CDC42EP5	227850_x_at	0.00544	1.1359441
ZNF83	221645_s_at	0.0000651	1.5067498	ASXL3	214162_at	0.00547	1.7883571
CENPF	207331_at	0.0000658	-2.2365709	GJB2	223278_at	0.00547	-1.9123699
HIST1H3F	206110_at	0.0000664	-1.9024805	PRRG3	220433_at	0.00547	1.3243467
BCAP29	225677_at	0.0000667	1.0455109	LINC00926	230245_s_at	0.00548	1.4131096
CTLA4	236341_at	0.0000673	-2.9337744	SMYD5	209516_at	0.00549	1.0220249
WNT2	205648_at	0.0000677	1.5869844	RHOV	241990_at	0.0055	-1.5974726
LOC100507557	241745_at	0.0000679	1.6641228	PELI2	219132_at	0.00551	1.6907202
ENPP2	209392_at	0.0000681	2.2965789	CLMP	226834_at	0.00551	1.70022
CKMT2-AS1	235443_at	0.0000687	1.124552	LOC102723897	225035_x_at	0.00553	1.0479552
KLHL20	204176_at	0.0000688	1.0264457	LOC102724814	1557263_s_at	0.00556	1.6512311
CAPN3	210944_s_at	0.0000694	1.7515459	IGFBP7	213910_at	0.00557	1.420933
KIAA1429	238818_at	0.0000694	1.0251714	ESPN	233979_s_at	0.00557	-1.6692496
PARD3-AS1	230545_at	0.0000695	1.964505	FCN3	205866_at	0.00557	-1.2051432
TRIM52-AS1	1557507_at	0.0000698	2.0546159	BTNL9	241496_at	0.00557	-1.4308628
TMEM35B	213698_at	0.0000698	1.0161611	WTAP	227621_at	0.00559	1.158301
PLA2G4C	209785_s_at	0.00007	1.6942562	PLP2	201136_at	0.00563	-1.3472035
SLC25A21-AS1	230307_at	0.0000701	2.0259656	SPPL3	1566897_at	0.00564	-1.5310004
SSPN	204963_at	0.0000704	2.2297736	IL1R1	202948_at	0.00564	1.0337113

CIRBP	230142_s_at	0.0000705	1.4441111	CCDC85A	235228_at	0.00566	2.1733405
GBAT2	230787_at	0.0000707	-1.1434474	LRRC20	218550_s_at	0.00566	-1.2446367
CORO1C	222409_at	0.0000711	-1.332665	LOC101927305	237771_s_at	0.00566	1.6850235
IRS1	242979_at	0.0000712	1.6910717	APOE	212884_x_at	0.00567	-1.0460384
ZNF638	1554248_at	0.0000713	1.0014416	KANSL1-AS1	230388_s_at	0.00567	1.066197
GPAT4	224776_at	0.0000716	-1.060132	SEMA5B	223610_at	0.00567	1.4199825
SSBP2	203787_at	0.0000719	1.2450962	LOC101927841	239823_at	0.00569	1.8487269
SERHL2	214243_s_at	0.0000727	1.9700557	CARS2	237555_at	0.00569	-1.7908832
PARPBP	227928_at	0.0000727	-2.0438847	HIC1	230218_at	0.00569	1.2187534
SEC24A	212902_at	0.0000736	-1.0685412	MESP1	224476_s_at	0.0057	-1.5757232
AGFG1	226561_at	0.0000738	1.2115492	RHOJ	243481_at	0.0057	1.5394556
CTSK	202450_s_at	0.0000738	1.3580756	OTUD4	203479_s_at	0.0057	-1.4243379
PTPN11	209896_s_at	0.0000739	-1.6674803	POLQ	207746_at	0.00571	-1.2654006
KIF18A	221258_s_at	0.000074	-1.2008429	SAA2-SAA4	208607_s_at	0.00572	-2.2338106
SIX4	231797_at	0.000074	-2.1237245	CDC27	217878_s_at	0.00572	-1.2987626
LRRCC1	231872_at	0.000074	1.0648676	SERPINB5	204855_at	0.00574	-2.3388084
LOC100506125	231232_at	0.0000747	2.1313002	IGFBP7-AS1	1557395_at	0.00574	1.7549836
LIFR	225571_at	0.0000748	1.4828416	UBASH3B	228353_x_at	0.00575	-1.4303293
RNF180	242033_at	0.000075	2.0978184	STAT5B	1555086_at	0.00575	1.5809795
TAOK1	227454_at	0.0000754	-1.2241462	CNIH4	222721_at	0.00577	1.6366261
DHFR	48808_at	0.0000754	-1.3559506	SEC14L2	232894_at	0.00578	-1.3609993
FOXA1	204667_at	0.0000756	-2.7717744	COL9A2	213622_at	0.00578	1.0067801
PHYKPL	226519_s_at	0.0000759	1.0204224	NR4A2	204621_s_at	0.0058	1.1908301
SULF1	212344_at	0.0000765	-1.9358668	SOAT1	221561_at	0.0058	-1.0314443
LOC101928068	235883_at	0.0000767	1.4986153	LRRC14	203495_at	0.0058	1.1054564
ARGLU1	228477_at	0.0000767	1.9052822	FAM50B	205775_at	0.00581	1.1167262
SFPQ	201585_s_at	0.000077	-1.2221861	FCHSD1	226698_at	0.00582	1.3670101

PDK3	221957_at	0.0000772	-1.0314005	ALDOA	238996_x_at	0.00582	-1.71899
SCYL2	221220_s_at	0.0000773	-1.6460403	NUB1	1559732_at	0.00582	1.7286039
CCNB1	214710_s_at	0.0000773	-1.9502089	BAZ2A	215437_x_at	0.00583	-1.3889658
TOX2	228737_at	0.0000773	-1.8052008	RSPO1	241450_at	0.00583	1.5507986
ACOX2	205364_at	0.0000774	1.9322474	PRRG3	229118_at	0.00584	1.4911814
TMEM170B	235798_at	0.0000778	1.625975	ATP1B1	201242_s_at	0.00584	-1.1287498
KLHL31	239398_at	0.0000778	1.9744463	DAGLB	225833_at	0.00585	-1.1216914
LETM2	1557415_s_at	0.0000782	1.2741842	LOC100507855	230630_at	0.00586	-1.2996372
MMP12	204580_at	0.0000785	-3.0437056	SOCS4	1552792_at	0.00586	-1.2326068
FAM69B	229002_at	0.0000795	1.6323567	LOC100506476	243680_at	0.00588	2.0313779
NARF	237299_at	0.0000795	-1.1415952	NIPSNAP1	201708_s_at	0.00591	-1.2346178
STAT3	208991_at	0.0000796	-1.1496263	PTPN11	209895_at	0.00594	-1.0758546
PRTG	229073_at	0.0000797	1.7807318	TMEM206	222752_s_at	0.00594	-1.0978277
LOC100129518	216841_s_at	0.0000798	-1.7318486	HUNK	1555935_s_at	0.00595	-1.7627501
RBM5	209936_at	0.0000798	2.1140275	GLB1L3	1569886_a_at	0.00598	2.3718504
SDCCAG3	230058_at	0.00008	1.1212087	ITCH	236235_at	0.00598	1.2461353
CBFA2T3	208056_s_at	0.0000802	1.4004939	ANXA1	201012_at	0.00599	-1.4513993
EBLN3P	225635_s_at	0.0000805	1.0441689	LOC105377458	1557762_at	0.00599	2.8769255
HLX	214438_at	0.0000809	-1.9393477	LINC00965	222184_at	0.006	1.4520906
WAC	230154_at	0.0000811	1.0318678	CXXC4	220277_at	0.00601	1.4207501
GULP1	204235_s_at	0.0000817	1.6749757	NAV2	222599_s_at	0.00602	-1.0192495
ZAK	225662_at	0.000082	1.083619	CYP1B1	202435_s_at	0.00603	1.2719688
MKI67	212020_s_at	0.0000829	-1.3858773	SELL	204563_at	0.00604	-1.2811612
MROH1	230361_at	0.0000829	-1.3410546	RCAN3	219864_s_at	0.00606	-1.6677179
BCL2L11	222343_at	0.0000832	-1.1280137	KCTD15	222664_at	0.00606	1.085924
WBSCR22	207628_s_at	0.0000834	-1.1156433	PLEKHH2	239568_at	0.00606	2.1971919
MAPK1	212271_at	0.000084	-1.0699896	GSR	205770_at	0.00606	-1.1072733

TNPO1	1556116_s_at	0.0000844	1.9834952	NT5E	1553995_a_at	0.00607	1.6055483
GK	207387_s_at	0.0000854	-1.5643473	LEFTY1	206012_at	0.00608	1.9200684
FANCB	1553244_at	0.0000855	-1.9028529	PBDC1	224177_s_at	0.00609	-1.0540366
FAM13A-AS1	243020_at	0.000086	1.1892153	C1orf56	230468_s_at	0.00609	1.5353968
COL28A1	239921_at	0.0000861	1.9822611	DPYD	204646_at	0.0061	1.6167906
SRGAP1	227484_at	0.0000871	-1.4687902	PCBP3	205663_at	0.00611	1.2787296
COL4A1	211980_at	0.0000871	-1.4335666	FAM111B	1557129_a_at	0.00615	-1.5654637
SLC22A23	223194_s_at	0.0000876	-1.2089488	GJA5	226701_at	0.00615	-1.3627927
NPL	240440_at	0.0000876	-1.915796	SLITRK6	232481_s_at	0.00616	2.5917964
ZNF22	218005_at	0.0000878	1.0420559	EDNRB	204271_s_at	0.00616	1.1344996
ABHD17C	225436_at	0.000088	-1.1053983	LOC102723845	235898_at	0.00616	1.1035147
CD44	229221_at	0.000088	-1.8179594	HK3	205936_s_at	0.00616	-1.6810017
ASAP1	224790_at	0.0000894	-1.2348329	LINC00550	1557717_at	0.00616	-1.7282119
CPT1A	203634_s_at	0.0000898	-2.0306833	SHANK2	213307_at	0.00617	1.3294155
CDCA5	224753_at	0.0000903	-1.6752967	CYP46A1	220331_at	0.00617	1.7209416
WDR61	215156_at	0.0000903	1.1549958	GZF1	234055_s_at	0.00618	1.1444683
JAZF1-AS1	1559650_at	0.000091	2.3596645	LUC7L3	242389_at	0.00618	1.0931783
KISS1R	242517_at	0.0000925	-2.2284833	TTN-AS1	1556043_a_at	0.00618	1.5101129
DEPDC1	232278_s_at	0.0000926	-2.7682302	GAMT	1552474_a_at	0.00621	1.266249
MEIS3P1	214077_x_at	0.0000928	1.0865795	LOC100996693	205265_s_at	0.00622	1.2679707
ABI2	211793_s_at	0.0000932	-1.5127086	ATP11B	212536_at	0.00622	1.1529264
RGL3	1556354_s_at	0.0000933	2.327749	COL4A2	211966_at	0.00622	-1.4753746
JAM2	229127_at	0.0000933	2.0115303	PSEN1	207782_s_at	0.00623	-1.3395535
KLF10	202393_s_at	0.0000936	1.0303085	JAM2	219213_at	0.00623	1.4467058
SSR1	200890_s_at	0.0000939	-1.1394279	KIAA2026	238490_at	0.00623	-1.2247216
LOC101928605	1555890_at	0.0000939	1.4693986	MAL	204777_s_at	0.00623	-2.3688536
PGGHG	219359_at	0.0000943	2.390188	NEBL	203961_at	0.00627	1.9726813

ANTXR2	228573_at	0.0000944	1.5326847	STAM2	208194_s_at	0.00628	-1.681354
NR4A1	211143_x_at	0.0000952	1.8165661	CALML4	64408_s_at	0.00629	-1.1123808
CCNB1	228729_at	0.0000964	-1.8962513	SEL1L3	212314_at	0.0063	-1.2359633
CDK1	203214_x_at	0.0000967	-1.6811587	EGR2	205249_at	0.00631	1.3251732
ALPPL2	210431_at	0.0000968	-3.0152098	FES	205418_at	0.00631	1.5245663
XPO5	223055_s_at	0.0000974	-1.2932953	PRRC2C	211947_s_at	0.00631	-1.334374
ZNF213-AS1	222170_at	0.0000977	1.7968664	GINS2	221521_s_at	0.00632	-1.260076
CD24	209772_s_at	0.000098	-1.5462668	GUSBP5	240063_at	0.00634	1.0725591
SETD6	219751_at	0.0000988	1.1933862	PCDH10	1552925_at	0.00634	1.5626054
MROH7	214147_at	0.0000989	2.3411322	EN2	207060_at	0.00635	1.7108509
QSOX1	201482_at	0.000099	-1.3496285	FAM110B	228790_at	0.00636	1.5920433
OMD	205907_s_at	0.000099	2.5406599	FCRL3	231093_at	0.00636	-1.1072255
METTL7A	207761_s_at	0.0000995	1.6687603	FBXO17	220233_at	0.00637	2.4506291
HERPUD1	217168_s_at	0.000101	1.1404536	PLK4	204886_at	0.00638	-1.1442673
SPATA20	218164_at	0.000101	1.3153169	ECHDC3	219298_at	0.00638	1.2078371
AIM1L	220289_s_at	0.000102	-2.6868444	PHTF1	215285_s_at	0.00639	-1.1525845
FMO5	205776_at	0.000102	1.8484002	CYS1	228739_at	0.00639	2.0128399
MYH10	213067_at	0.000102	-1.1897378	ARNTL2	224204_x_at	0.00639	-1.5300925
LMNB2	216952_s_at	0.000105	-1.320276	LAMP5	219463_at	0.00639	2.6636617
DHRS4-AS1	227446_s_at	0.000105	1.1104805	B3GAT1	219521_at	0.0064	1.6344817
CDC42	208727_s_at	0.000106	-1.0868329	CCKBR	210381_s_at	0.0064	-1.1293744
WT1-AS	206954_at	0.000106	2.3767618	CDC20B	1553655_at	0.00642	-3.0444837
ZCWPW1	220618_s_at	0.000107	1.9908497	INPP4B	205376_at	0.00643	-1.5043396
HIST1H2BC	214455_at	0.000107	-2.0672896	LOC100506302	243224_at	0.00643	1.0040452
LRRK2	229584_at	0.000107	2.4175255	SERPINB9	209722_s_at	0.00643	-1.5292268
CDK10	203468_at	0.000108	1.3650479	PAGR1	218300_at	0.00643	1.2657769
SCD	200831_s_at	0.000109	-1.4245965	AUTS2	243364_at	0.00643	1.6754127

SLC1A5	208916_at	0.000109	-1.2214431	LOC441179	239665_at	0.00645	2.0728622
KIAA0922	209760_at	0.000109	-1.0245835	SHISA9	1556619_at	0.00645	-1.565149
HAPLN1	230204_at	0.000109	-1.5307243	CCDC110	230900_at	0.00645	1.6399108
SRC	213324_at	0.000109	-1.7149821	LOC100505564	238376_at	0.00646	1.0955463
NT5E	203939_at	0.00011	2.4531659	C1orf43	1555226_s_at	0.00646	-1.5772761
LOC100288911	236656_s_at	0.00011	1.8407795	FGFBP3	238453_at	0.00647	1.2376613
CACNA2D1	227623_at	0.00011	2.0004141	ZNF135	206142_at	0.00647	2.0400816
PLA2G6	210647_x_at	0.000112	1.0064498	CCDC30	221171_at	0.00647	-1.0941565
ANXA2P2	208816_x_at	0.000112	-1.2272709	SPINK5	205185_at	0.00649	-2.3669984
ZNF485	1552427_at	0.000113	1.1037839	LINC00908	1558850_s_at	0.0065	1.5182511
BCAP31	200837_at	0.000113	-1.2030761	PAX6	235795_at	0.0065	-2.7411473
MARCKS	201670_s_at	0.000113	-1.5407523	L3MBTL1	206822_s_at	0.00652	1.2034641
CXCL9	203915_at	0.000113	-2.9427274	CSRNP3	235355_at	0.00653	1.6919568
UBE2T	223229_at	0.000114	-1.8108854	NRF1	211280_s_at	0.00653	-1.1775485
LOC101928291	232758_s_at	0.000114	1.2268497	SLC13A3	230687_at	0.00655	-1.4582813
SLC2A1	201249_at	0.000115	-1.9923634	AGER	217046_s_at	0.00656	1.2291891
APLN	244166_at	0.000115	2.094942	LOC101927668	237193_s_at	0.00656	1.5860076
SGSM1	230287_at	0.000115	2.399607	GUSBP1	235888_at	0.00658	1.2181729
HFM1	241469_at	0.000115	2.9138792	CMBL	227522_at	0.0066	1.6025035
GJA9-MYCBP	203360_s_at	0.000115	-1.1983626	FAM155A	230869_at	0.00661	2.2678749
NAV2	222598_s_at	0.000116	-1.5825863	ALDOA	214687_x_at	0.00661	-1.314932
ANKRD29	229308_at	0.000117	2.3029884	GRPEL1	212432_at	0.00662	-1.0167366
LINC00472	235771_at	0.000117	1.889468	LOC286272	232182_at	0.00662	1.4206869
CAST	208908_s_at	0.000117	-1.207323	RAB3IP	238853_at	0.00664	-1.0927189
PPP2CA	238719_at	0.000117	1.0151307	ATP2A1-AS1	230696_at	0.00664	-1.4980255
KANSL1L	230561_s_at	0.000118	1.1810303	NTRK3	228849_at	0.00664	1.336098
MTMR9LP	228593_at	0.000118	1.4256029	ATAD2	218782_s_at	0.00667	-1.4013331

FAM198B	223204_at	0.000118	2.2471862	SRGN	1554676_at	0.0067	-1.4343331
CNKSR2	229116_at	0.000118	2.7131631	CCL18	32128_at	0.0067	-1.907402
EMILIN3	228307_at	0.000119	1.7230342	HPGD	211549_s_at	0.00671	1.9287214
ARID4B	214943_s_at	0.000119	-1.7320829	IL20RA	219115_s_at	0.00671	1.8857149
FBXO11	219208_at	0.00012	-1.3734357	ATAD2B	232908_at	0.00672	-1.223975
HNMT	211732_x_at	0.000122	1.8516633	ZNF606	229707_at	0.00672	1.0148404
DCBLD1	226609_at	0.000122	1.124381	FPGT-TNNI3K	220415_at	0.00674	1.6389002
LDB2	206481_s_at	0.000124	1.4446806	MIR6787	202855_s_at	0.00677	-1.681018
MAGI2	207702_s_at	0.000125	2.6843335	ELK4	205994_at	0.00677	-1.0104597
GLUL	242281_at	0.000125	-1.2430055	NT5DC1	241962_at	0.00678	1.0414992
EPOR	209962_at	0.000125	1.9091172	FGF9	239178_at	0.00678	2.4249906
RBM47	222496_s_at	0.000126	-1.2437014	LIX1	230865_at	0.00679	2.680912
OLA1	224525_s_at	0.000126	1.2093667	SAE1	1555618_s_at	0.00681	-1.1520579
PHF21B	1562309_s_at	0.000127	2.9221322	HPGD	203914_x_at	0.00685	2.5068303
SLC7A6	203580_s_at	0.000127	-1.2845893	MTMR1	214975_s_at	0.00685	-1.6260375
AHSA2	212980_at	0.000127	1.6592509	ZNF689	227445_at	0.00687	1.1774447
NQO1	210519_s_at	0.000128	-2.3600707	AP1AR	242753_x_at	0.00692	-1.5821994
RBM4	227979_at	0.000128	1.0125141	NSUN6	222128_at	0.00692	1.1355277
DDIT4	202887_s_at	0.000129	-1.9449077	PINLYP	213556_at	0.00694	1.5034559
SLC12A2	204404_at	0.000129	-1.3152403	MGC16275	1558166_at	0.00694	1.217462
LINC00921	243547_at	0.000129	1.8455817	CEACAM21	214907_at	0.00695	1.580738
SNHG19	228049_x_at	0.00013	1.2316317	FRG1KP	235535_x_at	0.00698	1.2032925
GPM6A	209469_at	0.00013	3.1093783	CXCL14	222484_s_at	0.00699	-3.0667841
EPHA4	228948_at	0.00013	-3.0160592	IGLJ3	217148_x_at	0.007	-1.3049466
HPN	204934_s_at	0.00013	1.508671	FAM64A	221591_s_at	0.00704	-1.2254649
LINC00663	1555363_s_at	0.00013	1.4213045	COBLL1	229598_at	0.00704	-1.6012161
LRRC14	32062_at	0.00013	1.2496971	DCN	240556_at	0.00707	1.3815239

STRIP2	1555292_at	0.000131	-2.5316324	CASK	207620_s_at	0.00707	-1.3444423
PPM1A	227728_at	0.000131	1.313768	PCBP1-AS1	1562481_at	0.00708	1.2055138
ARFGEF3	242098_at	0.000131	-2.0717742	EIF3H	230570_at	0.0071	1.2349107
EFEMP1	201843_s_at	0.000131	2.586125	GATA2	209710_at	0.0071	1.3517566
KPNA1	202059_s_at	0.000132	-1.2067613	PAXBP1-AS1	239407_at	0.00713	1.3477739
LOC102724156	230991_at	0.000132	1.5745536	PSAT1	223062_s_at	0.00714	-1.315212
TK1	1554408_a_at	0.000132	-1.3379992	AMDHD1	229596_at	0.00714	1.6668983
MS4A4A	219607_s_at	0.000133	-2.0625612	MYO15B	227743_at	0.00715	1.0967488
RAD51-AS1	1560081_at	0.000133	1.2010938	RGL3	1556355_x_at	0.00716	1.1094348
CCBE1	242324_x_at	0.000133	2.1374223	GNAQ	211426_x_at	0.00716	-1.0822827
MIB2	226644_at	0.000133	1.3358496	ABCA1	216066_at	0.00716	-1.5418041
DEGS1	207431_s_at	0.000134	-1.3381686	TMED5	202195_s_at	0.00719	-1.0152255
FSD1L	223985_at	0.000135	-2.3100231	RSF1	223818_s_at	0.00722	-1.1719703
EIF3A	200596_s_at	0.000136	-1.0001421	RPS27A	200017_at	0.00723	1.0902964
LOC101060604	1555216_a_at	0.000136	1.9595171	LRP5L	214873_at	0.00723	1.020773
RBFOX3	235468_at	0.000136	2.200567	LOC158434	1563827_at	0.00725	2.1818853
CD82	203904_x_at	0.000137	-2.0123418	LACTB	1552486_s_at	0.00726	-1.1104467
CEBPG	204203_at	0.000138	-1.2789355	MIATNB	232340_at	0.00726	1.0852414
PYGB	201481_s_at	0.000139	-1.7738815	FOXA2	210103_s_at	0.00727	2.0791126
RARRES2	209496_at	0.00014	2.3275842	CLDN1	218182_s_at	0.00729	-1.6352285
OAZ3	222075_s_at	0.00014	-1.8369832	MIB2	241541_at	0.00729	1.1126796
FANCA	203805_s_at	0.00014	-1.4882161	PDGFC	222719_s_at	0.00729	-1.5673391
BEND7	227341_at	0.00014	2.4865204	ITPR1-AS1	239764_at	0.00732	1.3959904
WDR86	243526_at	0.000141	2.4003777	RHEBL1	1570253_a_at	0.00733	-1.2335996
GLT8D2	221447_s_at	0.000141	1.4932043	CSAD	221139_s_at	0.00734	1.1123793
EFEMP1	201842_s_at	0.000142	2.3804313	TMPO	209754_s_at	0.00734	-1.3607181
ABCA17P	231164_at	0.000142	2.419169	ST6GALNAC5	230482_at	0.00736	-1.6704886

TCEAL1	204045_at	0.000144	1.1756624	NT5M	219708_at	0.00737	1.5158605
FGF14-AS2	230351_at	0.000144	2.0908846	DMBX1	234776_at	0.00738	-1.8317231
MIR6883	36829_at	0.000145	1.0011874	GALM	235256_s_at	0.0074	-1.0364537
SIX1	228347_at	0.000145	-3.2799825	NXPE3	243606_at	0.0074	1.0811916
COX10-AS1	1557192_at	0.000146	1.1109196	LOC101929713	1560373_a_at	0.00742	1.3880009
ZNF599	228093_at	0.000146	1.08725	ZSCAN16-AS1	1557062_at	0.00742	1.2282519
CCHCR1	42361_g_at	0.000146	1.2142645	CDCA7	230060_at	0.00742	1.2631715
ENO1-AS1	237028_at	0.000146	1.605782	PLEKHF2	222699_s_at	0.00742	-1.1516398
MBIP	218411_s_at	0.000147	1.1244049	HMGCR	202539_s_at	0.00744	-1.2002195
UBE2H	217799_x_at	0.000147	-1.609752	ABCC8	210246_s_at	0.00745	1.7491897
SMARCA4	208793_x_at	0.000147	-1.6597495	FKBP7	231130_at	0.00746	1.1542892
TANK	210458_s_at	0.000148	-1.2368101	GNAS-AS1	232881_at	0.00746	1.8099768
UHMK1	235003_at	0.000148	-1.542871	ZNF300	228144_at	0.00747	2.0366127
MTAP	216685_s_at	0.000148	-1.682274	KCNT2	244455_at	0.00748	1.8518493
DYX1C1-CCPG1	214152_at	0.000148	1.0189746	CPLX1	223500_at	0.00748	1.1143908
CPEB3	239765_at	0.000149	1.2816121	GLT1D1	229770_at	0.00748	1.4525878
TTC5	243007_at	0.00015	1.0340317	TNFAIP6	206025_s_at	0.0075	-1.4497943
CCNA2	203418_at	0.00015	-1.7330107	TPK1	223686_at	0.00752	-1.6001779
ZNF780B	242969_at	0.00015	1.0222329	MYO3B	1555257_a_at	0.00752	-1.6151398
LOC100506718	204358_s_at	0.00015	1.3221675	SAA3P	222226_at	0.00752	-1.163155
NFYC	211797_s_at	0.00015	-1.814768	MYO1B	212364_at	0.00756	-1.0317181
MFI2-AS1	239521_at	0.000151	1.7802749	CMAHP	229604_at	0.00758	1.4971605
KLF2	219371_s_at	0.000151	1.3889779	ANGPT1	205609_at	0.00759	1.4206028
PPP1R14C	226907_at	0.000152	-2.5404601	AVPR1A	238835_at	0.00763	1.9039103
SMARCA2	217707_x_at	0.000154	1.1584361	TROVE2	207520_at	0.00764	-1.0253626
LOC105371352	237015_at	0.000154	2.2904137	C1orf64	229381_at	0.00766	1.5691837
SLC24A3	219090_at	0.000154	2.7018909	EGLN1	224314_s_at	0.00767	-1.3264595

ANGPTL1	224339_s_at	0.000155	2.7699277	CALD1	235834_at	0.00768	1.1425981
FAM162B	228875_at	0.000155	1.5314413	CENPV	226610_at	0.00771	2.2537528
LOC101927365	238069_at	0.000156	1.0704552	SOCS6	214462_at	0.00776	-1.1462038
UBE2I	208760_at	0.000157	1.0471131	LOC100507855	204348_s_at	0.00777	-1.2790487
PAPPA	224940_s_at	0.000157	2.086362	TSHZ1	223282_at	0.00778	1.0701609
MUM1	229033_s_at	0.000157	1.6031012	SLC16A1	209900_s_at	0.00779	1.5136629
SREK1	238781_at	0.000158	1.068814	MYNN	237510_at	0.00782	1.0429164
TMEM132A	218834_s_at	0.000158	-1.0902141	CD28	206545_at	0.00784	-1.2545261
SREK1	235611_at	0.000158	1.2539589	TRIM66	237679_at	0.00792	-1.1280432
TGFBR3	204731_at	0.000158	1.6700824	TDRP	230903_s_at	0.00793	1.6849116
ARL10	228843_at	0.00016	1.5777999	LOC100129098	1569361_a_at	0.00794	1.1948287
PEX13	205246_at	0.00016	-1.706525	LMO7DN	239553_at	0.00796	1.5716894
HMGB3	225601_at	0.00016	-1.4728652	PAIP2B	221868_at	0.00796	1.0672234
MUM1	223347_at	0.00016	1.1021038	LOC101930370	1562053_at	0.00796	1.5022489
G3BP2	208840_s_at	0.00016	-1.2736549	NLGN1	205893_at	0.00797	1.7216195
DHTKD1	209916_at	0.000162	-1.0461144	DMD	203881_s_at	0.008	1.3486662
VEGFA	210513_s_at	0.000162	-1.4599507	SPON1	213993_at	0.008	2.8323435
VDR	204254_s_at	0.000162	-1.825056	CLIP1	1558924_s_at	0.00801	-1.6439846
LENG8	224673_at	0.000164	1.0731794	MEDAG	227058_at	0.00803	-1.1165611
ZNF709	1553247_a_at	0.000164	1.3486312	COBL	213050_at	0.00805	1.2774115
TBC1D16	228488_at	0.000164	-1.088348	SETD2	220946_s_at	0.00806	-1.0684988
SSBP2	1558604_a_at	0.000165	1.5641156	HIST3H3	208572_at	0.00808	1.4780031
NR4A2	204622_x_at	0.000165	1.7813902	COL5A1	203325_s_at	0.00808	1.2675879
CCT5	208696_at	0.000165	-1.6039065	GAMT	205354_at	0.00809	1.604962
CCDC88C	227228_s_at	0.000167	-1.2336666	PRR5L	219383_at	0.00811	-1.1809588
GABBR1	238569_at	0.000168	1.586793	SNORA11E	223313_s_at	0.00812	1.9466486
RC3H2	220202_s_at	0.000168	-1.0277651	ZMYM2	241800_x_at	0.00812	-1.1535008

NAV3	204823_at	0.000169	2.9428641	ATL2	237968_at	0.00812	-1.5748357
GABRB3	229724_at	0.00017	2.1356185	NPRL2	210373_at	0.00812	1.2602504
IQGAP3	229538_s_at	0.00017	-1.9002112	HOXC8	221350_at	0.00814	-1.3357107
LOC101926913	215627_at	0.00017	1.7648432	ADAMTSL3	213974_at	0.00814	1.3851447
DCUN1D5	239425_at	0.00017	1.488651	GNPDA2	227022_at	0.00818	1.001007
MIR6883	202861_at	0.000171	1.6582339	CR1L	239205_s_at	0.00819	-1.0422879
TTL	224908_s_at	0.000171	-1.2458801	PHF1	202928_s_at	0.00819	1.1166225
PPID	228469_at	0.000171	1.136452	ASAP3	219103_at	0.00819	1.1033008
ALDH6A1	221589_s_at	0.000172	1.371179	SPATA6L	237363_at	0.0082	1.6080329
ENO2	201313_at	0.000172	-1.7755999	FRA10AC1	244767_at	0.0082	1.1267994
KCNJ15	210119_at	0.000174	-2.9816856	CTSZ	210042_s_at	0.00821	-1.2185271
AIFM1	205512_s_at	0.000174	-1.4169744	OBSCN	216085_at	0.00822	1.3288484
IDNK	239086_at	0.000177	1.2071677	PRRX1	226695_at	0.00824	1.6118026
ATP1A2	203296_s_at	0.000178	3.1359676	FIGN	242828_at	0.00825	-1.4597792
GAS7	202192_s_at	0.000178	1.1488134	CHAF1A	203976_s_at	0.00825	-1.3707858
LOC283177	1557207_s_at	0.000178	1.9888734	COX2	1553570_x_at	0.00825	-1.0016006
ARRDC4	225283_at	0.000178	1.4515193	PAICS	201014_s_at	0.00831	-1.0780413
SYNCRIP	209025_s_at	0.000178	-1.1216211	BCL2	203684_s_at	0.00833	1.5985663
HLF	204753_s_at	0.000178	2.503838	SEMA3E	206941_x_at	0.00833	2.5599832
GNL3L	205010_at	0.000178	-1.0369929	CH17-408M7.1	1556235_at	0.00836	1.6470243
LOC101927752	227092_at	0.000179	1.6393314	ARPC4-TTLL3	210129_s_at	0.00837	1.301662
IGF2BP3	203819_s_at	0.000181	-3.0647852	FLNB	208613_s_at	0.00839	-1.0267008
HCG4	206685_at	0.000182	2.4758143	ITGBL1	214927_at	0.0084	1.7661861
OAS3	218400_at	0.000183	-2.0119399	PGK1	200737_at	0.00841	-1.4077952
RGS7	206290_s_at	0.000183	2.729509	FAM86B3P	234049_at	0.00841	1.7420882
NCF2	209949_at	0.000183	-1.5743681	LOC728613	229283_at	0.00841	1.0161267
C8orf88	1557961_s_at	0.000183	2.3053354	CPAMD8	227721_at	0.00842	1.0610946

SAR1A	210790_s_at	0.000183	-1.5189881	GLIPR1	226142_at	0.00843	1.0892025
ADAMTS9-AS2	1562295_at	0.000185	2.0652816	RMI2	226456_at	0.00845	-1.1647647
UGGT1	222569_at	0.000185	-1.0331263	FBLN1	202994_s_at	0.00845	1.9592943
SMARCC1	201075_s_at	0.000185	-1.0080214	CUX2	1566528_at	0.00847	1.7971828
LSM5	202903_at	0.000187	1.1275121	ZBTB20	213158_at	0.00848	1.0117427
CPQ	203501_at	0.000188	1.5288342	PDE7B	230109_at	0.00851	1.0572456
RAD51	205024_s_at	0.000189	-1.4324842	ZNF385B	229019_at	0.00852	2.4697348
PRKAG2-AS1	229156_s_at	0.000189	2.3282549	PDK3	230085_at	0.00852	-1.2787673
PGM5	226303_at	0.00019	2.1073155	PTPRG	227126_at	0.00854	1.0443873
XRN1	1555785_a_at	0.00019	-1.2834424	PPP2R2B	213849_s_at	0.00856	1.0380378
ID4	209293_x_at	0.00019	1.4618801	ACACB	49452_at	0.00858	1.1537556
ZBTB47	226500_at	0.00019	1.1917839	ZNF826P	1569191_at	0.00859	1.713585
IL4R	203233_at	0.00019	-1.1198051	OBSL1	227573_s_at	0.00861	1.4375444
SYTL3	238423_at	0.00019	-1.2272647	TGFBRAP1	225653_at	0.00861	-1.0906879
ACTR2	200729_s_at	0.00019	-1.0882002	SPTLC2	216202_s_at	0.00863	-1.3646196
GLYCTK-AS1	242692_at	0.000191	1.6171634	HNMT	204111_at	0.00864	1.4899679
GTSE1	215942_s_at	0.000192	-1.3620131	SAMD3	236782_at	0.00866	1.0550018
CA2	209301_at	0.000192	-2.3438932	CCBE1	243805_at	0.00866	1.8785459
ATP8A1	213106_at	0.000193	1.3023809	TMCO3	220241_at	0.00866	-1.1794082
XYLT1	213725_x_at	0.000193	1.7443498	CR1L	239206_at	0.00866	-1.8002508
RDH10	227467_at	0.000194	-1.8822845	LOC101928424	239241_at	0.0087	1.122347
CTSC	225646_at	0.000194	-1.2651087	WT1	206067_s_at	0.00871	2.1178057
CLEC11A	211709_s_at	0.000195	1.2371998	CYP2A7P1	216340_s_at	0.00872	-1.304998
RDH10	226021_at	0.000197	-1.7577685	PHLDA1	225842_at	0.00872	-1.5343285
TRPM3	239291_at	0.000198	2.742534	ZNF114	1552946_at	0.00872	1.6527075
SGTB	228745_at	0.000199	1.1137847	PHOSPHO2-KLHL23	237756_at	0.00872	1.3545332

CACNA1G	210380_s_at	0.0002	1.8037255	GPR83	222953_at	0.00877	-2.0425233
PCMT1	210156_s_at	0.0002	-1.1404712	GATAD2B	238076_at	0.00878	1.0098923
CDC14A	205288_at	0.0002	1.2136173	LRCH3	240926_at	0.0088	-1.0609797
THAP6	230169_at	0.0002	1.1513303	RIMS3	210991_s_at	0.00887	-1.3270339
DPH7	225586_at	0.0002	1.1404184	GAREM1	237262_at	0.00888	-1.0633556
MFI2-AS1	243629_x_at	0.0002	1.4538247	NFATC2	226991_at	0.00889	-1.2771671
NSUN5P2	213460_x_at	0.0002	1.2279302	TDRD6	232692_at	0.00889	1.3966766
FBRS	238771_at	0.000201	1.4038962	HSD17B11	217989_at	0.0089	1.0376908
EPHX2	209368_at	0.000203	2.0054528	PLEKHA6	205093_at	0.00894	-1.7034409
MAGI2-AS3	229480_at	0.000203	1.9947533	CCNE2	211814_s_at	0.00894	-1.325014
GALK2	205219_s_at	0.000203	-1.430814	NSUN7	238983_at	0.00895	1.2280393
SLITRK6	232176_at	0.000203	2.7180709	HSD17B12	1559518_at	0.00897	1.0026351
ZSCAN18	217593_at	0.000204	1.6424713	ACKR3	1559114_a_at	0.00898	1.6604662
TOLLIP-AS1	1555865_at	0.000204	1.1525987	PLA2G4B	219095_at	0.00899	1.0877816
SGPP2	226560_at	0.000204	-1.3946766	STON2	242583_at	0.009	-1.2176023
RUSC2	213066_at	0.000205	1.6937336	HBB	211696_x_at	0.00902	1.3434605
RNF146	221430_s_at	0.000205	-1.1866747	MASP1	235770_at	0.00907	1.2282002
ACP1	201629_s_at	0.000205	-2.5045589	TC2N	1553132_a_at	0.00907	-1.2921008
COL6A1	212091_s_at	0.000205	2.4805204	EXOC3	228847_at	0.00908	1.1296351
EEF2KMT	222013_x_at	0.000205	1.355866	PGAP1	220576_at	0.00909	1.5918746
LOC100272217	243784_s_at	0.000206	1.6335635	ZNF442	208081_s_at	0.00909	1.1904897
ITGB1	1553530_a_at	0.000206	-2.0348862	SLC22A3	205421_at	0.0091	2.1538641
TMEM120B	219154_at	0.000206	1.4587105	RBPMS-AS1	240929_at	0.00914	1.0866538
ARHGEF28	1554003_at	0.000207	1.6286813	FOXP2	243278_at	0.00914	2.3239992
PRDM11	229687_s_at	0.00021	1.3222789	F2R	203989_x_at	0.00914	-1.0083722
WDR20	227541_at	0.00021	-1.0018538	BHLHA15	235965_at	0.00914	-1.2089831
LOC202025	236079_at	0.00021	1.5308485	PLEKHM1	216200_at	0.00919	-1.4515225

CLASP2	1555469_a_at	0.000211	-1.9269151	S100A10	200872_at	0.00919	-1.2297072
SUDS3	233841_s_at	0.000211	-1.108205	GMDS-AS1	232335_at	0.00919	1.1469779
MAGEL2	219894_at	0.000212	2.5097173	YIF1B	231211_s_at	0.00921	1.5979666
RMND5A	212479_s_at	0.000212	-1.2375173	RAPH1	225189_s_at	0.00923	-1.015441
SOD3	205236_x_at	0.000212	1.3886477	TMEM37	1554485_s_at	0.00925	-2.161207
KCNJ2	206765_at	0.000212	-1.9393293	ZFAND4	244206_at	0.00925	-1.5634915
COL27A1	225292_at	0.000213	1.4166745	MAP1B	212233_at	0.00926	-1.3000736
UNKL	229908_s_at	0.000213	1.0054936	CDH3	203256_at	0.00929	-1.4898197
YBX1	208628_s_at	0.000214	-1.1600666	PRPF31	202407_s_at	0.00931	-1.7844319
WIPF1	202665_s_at	0.000214	-1.0104968	AICDA	219841_at	0.00935	-1.5759388
GK5	241384_x_at	0.000217	1.3478608	TUSC2	203272_s_at	0.00935	-1.2717404
MRPL42	217919_s_at	0.000218	-1.1367658	EIF2S3	205321_at	0.00938	-1.1316446
RGS5	209071_s_at	0.00022	-1.7703969	GATA6	210002_at	0.00942	1.7522814
ADAMDEC1	206134_at	0.00022	-2.0937188	MYO6	203215_s_at	0.00942	-1.0025296
ETV7	221680_s_at	0.00022	-1.5284014	ZNF667-AS1	244741_s_at	0.00948	1.6376559
SH3GLB1	209091_s_at	0.00022	-1.2035705	TRIP11	235973_at	0.00948	1.4708356
ETAA1	219216_at	0.00022	1.0185227	TRAF3IP2	215411_s_at	0.0095	1.290254
CCDC6	225010_at	0.000221	-1.2364975	MPDU1	209208_at	0.0095	-1.5561363
PAPPA	201981_at	0.000222	1.8650164	AKAP12	231067_s_at	0.0095	2.1873413
DDX19A	202578_s_at	0.000222	-1.1418582	ZNF853	232884_s_at	0.00951	1.090604
CD44	212014_x_at	0.000223	-1.3963221	GNAL	213924_at	0.00952	1.4739581
ROCK2	202762_at	0.000223	-1.0143534	LMO4	229537_at	0.00955	1.2297105
PGK1	217356_s_at	0.000224	-2.2740191	DOK3	223553_s_at	0.00957	-1.0565249
NEBL-AS1	239894_at	0.000224	2.0348841	RBM33	1554096_a_at	0.00957	-1.3834564
ZNF502	229532_at	0.000224	1.4597906	SATB1	203408_s_at	0.00958	1.769446
BLCAP	201032_at	0.000224	-1.0356325	FAM229A	1556560_a_at	0.00958	1.1304127
YWHAZ	200638_s_at	0.000224	-1.6346675	NEDD1	1552417_a_at	0.00961	-1.7522975

HAPLN1	205523_at	0.000225	-2.4545043	MGC10814	224134_at	0.00962	-1.2154782
TTN	242771_at	0.000225	2.1093569	ZBTB46	227358_at	0.00963	1.3490784
CA3	204865_at	0.000226	2.1558567	SMURF1	212668_at	0.00968	-1.2594849
DNM3OS	232090_at	0.000226	1.8983137	CLN6	1567080_s_at	0.00969	-1.2434602
GSTM2	204418_x_at	0.000226	1.3361887	DBT	211196_at	0.00971	-1.3782813
PRR5L	1555486_a_at	0.000226	-1.3002877	LOC105377832	1556147_at	0.00972	1.3186805
FUBP1	214094_at	0.000226	1.7295563	PKMYT1	204267_x_at	0.00973	-1.0360148
TSHZ3	223392_s_at	0.000227	1.3818532	LILRA5	1555634_a_at	0.00973	-1.0474653
TCEA3	226388_at	0.000227	2.3182827	SCAND2P	230612_at	0.00975	1.1546841
HNRNPC	200751_s_at	0.000227	-1.1941713	LOC101927272	232384_s_at	0.00976	1.6034066
RAD23B	201222_s_at	0.000227	-1.3021817	LOC101928797	1570206_at	0.00977	1.9113092
PIF1	228252_at	0.00023	-1.5801615	CDHR5	220075_s_at	0.00979	1.0626402
NR1D1	204760_s_at	0.00023	1.554972	HS2ST1	230465_at	0.00981	-1.2439863
GABRB3	227690_at	0.000231	2.3315254	TMEM47	209656_s_at	0.00982	1.3328022
AVPR1A	242680_at	0.000232	2.1718051	ZFP36L2	227681_at	0.00983	1.3481743
CEP68	239442_at	0.000232	1.3997518	PHYHD1	226846_at	0.00983	1.225267
GAPDH	213453_x_at	0.000232	-1.5162296	CYAT1	215121_x_at	0.00983	-2.3768071
ASF1B	218115_at	0.000233	-1.1537001	DAPP1	222859_s_at	0.00985	-1.2438896
SLC7A5	201195_s_at	0.000233	-1.4969876	SAPCD2	225777_at	0.00986	-1.0646929
WARS	200629_at	0.000234	-1.2143228	COBLL1	211032_at	0.00986	-1.9755655
APLP2	214875_x_at	0.000235	-1.7075642	INPP5F	230363_s_at	0.00988	-1.1558415
TYMP	204858_s_at	0.000235	-1.1970911	HABP4	209819_at	0.0099	1.3155864
LOC283588	1557113_at	0.000237	1.6511492	LOC728705	230579_at	0.00993	1.131173
GFPT1	202722_s_at	0.000238	-1.084938	ANKH	220076_at	0.00994	1.2782472
TBRG1	226318_at	0.000238	1.0886437	ADAMTS17	1552727_s_at	0.00998	1.3872718
MON2	212755_at	0.000238	1.2800817	SCARA5	229839_at	0.00998	1.6151124
MOB1A	201299_s_at	0.000238	-1.4588487	PRSS12	213802_at	0.00998	2.0260279

MSX2	205555_s_at	0.000239	2.8952697
UCKL1	218533_s_at	0.000239	1.1842752
FEN1	204768_s_at	0.000239	-1.004431

TFDP1	204147_s_at	0.00998	-1.1361632
RBFADN	1558628_at	0.00999	1.5800599
